# Ca(OH)_2_-Catalyzed Condensation of Aldehydes with Methyl ketones in Dilute Aqueous Ethanol: A Comprehensive Access to α,β-Unsaturated Ketones

**DOI:** 10.1038/srep30432

**Published:** 2016-07-22

**Authors:** Lei Yu, Mengting Han, Jie Luan, Lin Xu, Yuanhua Ding, Qing Xu

**Affiliations:** 1 Jiangsu Co-innovation Center for Prevention and Control of Important Animal Infectious Diseases and Zoonoses, School of Chemistry and Chemical Engineering, Yangzhou University, Yangzhou, Jiangsu 225002 China; 2 Jiangsu Yangnong Chemical Group Co. Ltd., Yangzhou, Jiangsu, 225009, China

## Abstract

Cheap, abundant but seldom-employed Ca(OH)_2_ was found to be an excellent low-loading (5–10 mol%) catalyst for Claisen-Schmidt condensation of aldehydes with methyl ketones under mild conditions. It was interesting that dilute aqueous ethanol (20 v/v%) was unexpectedly discovered to be the optimal solvent. The reaction was scalable at least to 100 mmol and calcium could be precipitated by CO_2_ and removed by filtration. Evaporation of solvent directly afforded the product in the excellent 96% yield with high purity, as confirmed by its ^1^H NMR spectrum.

α,β-Unsaturated ketones, including dimethylidene acetone derivatives, are not only important building blocks in organic synthesis, but also key chemicals in many fields including perfumery, biochemistry, agriculture, food chemistry, polymer and material science, and others^1^,^2^,^3^,^4^. Therefore, the synthesis of these compounds is of great importance in both academic and industrial circles. Among reported works, Claisen-Schmidt condensation appears to be the most practical method to prepare α,β-unsaturated ketones owing to its directness, clean procedures and accessible starting materials. Despite being discovered over 100 years ago, the enthusiasm for Claisen-Schmidt condensations never reduces and in recent years, a series of novel catalysts have been developed for this reaction, such as solid bases^5^,^6^, nano catalysts^7^,^8^, ionic liquid catalysts^9^, fluorous based catalysts^10^,^11^, metal-organic frame works (MOFs)^12^ and organocatalysts^13^,^14^. Nevertheless, cheap and abundant NaOH would be expected to be the most common catalyst for the reaction due to its availability in laboratory, and indeed this method is still widely employed up to the present^15^,^16^,^17^. But reactions performed in strong alkaline conditions are corrosive to equipment and generate unmanageable and corrosive solid waste. These drawbacks have limited the large-scale application of NaOH. Moreover, methods for the synthesis of dimethylidene acetone derivatives, especially for those dissymmetrically substituted compounds, have not been well documented yet. Thus, developing novel alternative synthetic methodologies with broad scope using mild and common base catalysts is not only desirable but timely for the field.

Calcium hydroxide is also a readily accessible base and compared with NaOH, it is much cheaper and less alkaline. Moreover, Ca(OH)_2_ is easily neutralized and precipitated by CO_2_, which is beneficial from the point of industrial use. However, despite several well-known applications in industrial production, examples of the employment of Ca(OH)_2_ as a base catalyst in organic synthesis are rare^18^. As part of our continuing cooperative research projects with industrial partners to develop green synthetic methodologies^19^,^20^,^21^,^22^,^23^,^24^,^25^,^26^,^27^,^28^, we reported an organoselenium-catalyzed green oxidation of α,β-unsaturated ketones to prepare vinyl esters, which serve as versatile copolymers in material science^24^. To facilitate industrial application, a green and practical synthesis of α,β-unsaturated ketones (the starting material for vinyl ester synthesis) was desired. To that end, we investigated the Ca(OH)_2_-catalyzed Claisen-Schmidt condensations to prepare α,β-unsaturated ketones. During this work, dilute aqueous ethanol was unexpectedly found to be the optimal solvent and calcium could be precipitated by CO_2_ and removed by filtration to afford high purity products after solvent evaporation. The method allows comprehensive access to versatile α,β-unsaturated ketones, including the challenging dissymmetrically substituted dimethylidene acetone derivatives. Herein, we wish to report our findings.

## Results

**Figure 1:**
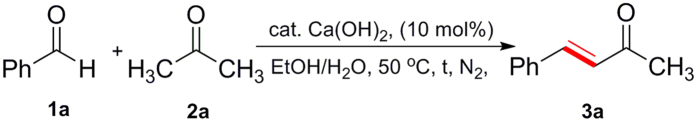
Condensation of 1a with 2a.

We initially chose the Ca(OH)_2_-catalyzed Claisen-Schmidt condensation of benzaldehyde **1a** with acetone **2a** as the model reaction to find optimal conditions (Fig. 1). After heating **1a**, **2a** and 10 mol% of Ca(OH)_2_ in EtOH at 50 °C for 20 h, the product benzylideneacetone **3a** could be isolated in 68% yield (Table 1, entry 1). During the reaction process, we observed Ca(OH)_2_ precipitation at the bottom of the tube, which implied the low efficiency of alkali utilization. Therefore, water was then added to increase the Ca(OH)_2_ solubility. When the reaction was performed in EtOH/H_2_O (80:20), it was significantly accelerated and finished in 16 h, giving **3a** in 69% yield (entry 2). The reaction was further accelerated and the product yields were enhanced greatly by increasing the proportional of water in the solvent (entries 3–4). Surprisingly, EtOH/H_2_O (20:80) as solvent gave the highest product yield in 85% (entry 4). Increased ratios of water in the solvent only resulted in reduced product yield and extended reaction times (entry 5), possibly due to the reduced substrate dissolution that inhibited the reaction. When the reactions were taken in highly diluted aqueous EtOH (entry 6) or pure water (entry 7), no product **3a** was observed. It is notable that the combination of EtOH with water played a key role in this reaction. A series of parallel reactions showed that the effect of EtOH/H_2_O was not only solvent for both organic substrates and inorganic base, but it also activated the Ca(OH)_2_. Experiments performed in acetone or acetone/EtOH resulted in very low product yields despite the reaction temperature. For details, please see the Supplementary Information.

**Figure 2:**
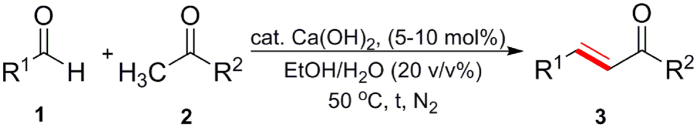
Substrate extension of the Ca(OH)_2_-catalyzed Claisen-Schmidt condensation.

With the optimized conditions in hand, a series of aldehydes **1** and ketones **2** were then employed to examine the scope of the reaction (Fig. 2). Results in Table 2 clearly show that the electron-enriched aldehydes had reduced reactivities for this reaction, which resulted in both extended reaction times and decreased product yields (Table 2, entries 2–5 *vs.* 1). For 4-methoxybenzaldehyde **1e**, the reaction should be carried out at room temperature with excess acetone, otherwise the dialkylated product (1*E*,4*E*)-1,5-bis(4-methoxyphenyl)penta-1,4-dien-3-one **4c** was obtained instead of the desired (*E*)-4-(4-methoxyphenyl)but-3-en-2-one **3e** (Table 2, entry 5). The electron-deficient aldehydes obviously had higher reactivities and their reactions were accelerated, but resulted in reduced product yields due to the generation of a series of unidentified byproducts (Table 2, entries 6–11). The reactions of electron-deficient aldehydes could be improved using milder conditions. For example, treating 2-chlorobenzaldehyde **1h** with acetone under the standard reaction conditions (50 °C) afforded the product **3h** in only 40% yield, but the yield could be improved of room temperature (ca. 25 °C), affording **3h** in 52% yield (Table 2, entry 8). Similarly, for 4-(trifluoromethyl)benzaldehyde **1j**, reaction with acetone under standard conditions gave **3j** in very low yield, but was also improved to 72% at room temperature (Table 2, entry 10). The reaction of 4-nitrobenzaldehyde **1k** with acetone led to poor product yield, but this was improved at room temperature (Table 2, entry 11). Bulky aldehyde **1l** was also tested, giving the desired product **3l** in moderate yields (Table 2, entry 12). We were also interested in the synthesis of heterocycle containing α,β-unsaturated ketones because of their bioactivities and potential applications in medicinal chemistry. The reaction of picolinaldehyde **1m** with acetone was tested, but gave **3m** in very low yield. Fortunately, the reaction could be improved to give **3m** in moderate yield under milder conditions using excess acetone (Table 2, entries 13). Interestingly, the reaction of thiophene-2-carbaldehyde **1n** with acetone afforded **3n** quickly in the excellent 90% yield under the standard conditions (Table 2, entry 14). The α,β-unsaturated aldehyde **1o** was also good substrate for the reaction, giving **3o** in 91% yield (Table 2, entry 15). The reaction of aliphatic aldehyde gave the product in low yield (Table 2, entry 16).

Besides acetone, other methyl ketones could also be employed. The reaction of acetophenone **2b** with benzaldehyde **1a** led to **3p** in 71% in 18 h (Table 2, entry 17). But the electron-riched substrate **2c** obviously had lower reactivity and the reaction did not complete even after 48 h (Table 2, entry 18). Reaction of the electron-deficient substrate **2d** with **1a** led to their product **3r** in 68% yield in 40 h, with a series of unidentified by-products observed by TLC (Table 2, entry 19). Reactions of the alkyl methyl ketones **2e** and **2f** with **1a** afforded the corresponding products **3s** and **3t** in moderate yields (Table 2, entries 20–21). A more detailed substrate expansion table was also given in the Supplementary Information.

**Figure 3:**
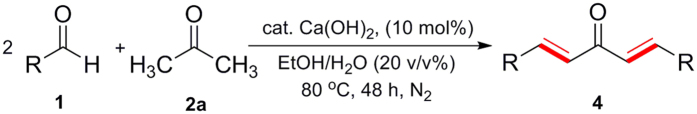
Synthesis of symmetrically substituted dimethylidene acetone derivatives.

The synthesis of the dimethylidene acetone derivatives was our next concern because of the great application potential of these bioactive compounds (Fig. 3). Fortunately, during the previous optimization study, we serendipitously found that the symmetrically substituted dibenzylidene acetone **4a** could be easily synthesized in good yield from **1a** and **2a** at 80 °C (Table 3, entry 1). As shown in Table 3, other symmetrically substituted dimethylidene acetone derivatives could be smoothly synthesized in this way. Obviously, the electron-enriched aldehydes **1b** and **1e** had poor reactivity for the reaction, giving **4b** and **4c** in only 31–39% yields (Table 3, entries 2–3). The electron-deficient aldehydes **1f** and **1j** were much more activated (Table 3, entries 4–5), and the reaction of **1j** with acetone even led to **4e** in excellent 92% yield (Table 3, entry 5). Heterocycle-substituted aldehydes were also suitable substrates for the reaction, giving corresponding products in moderate to good yields (Table 3, entries 6–7).

**Figure 4:**
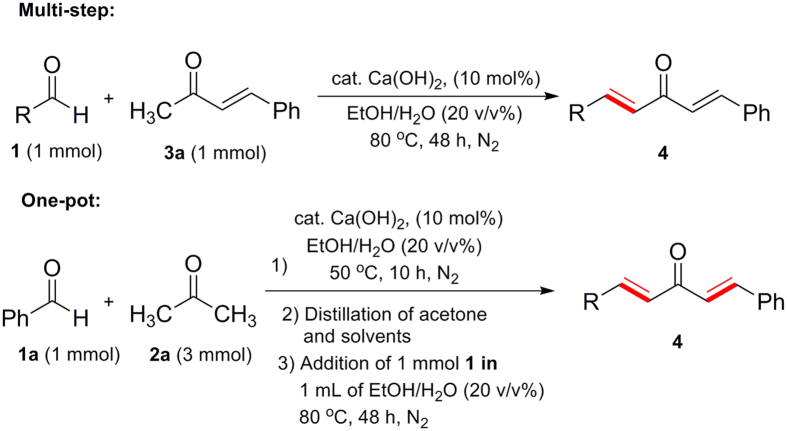
Synthesis of dissymmetrically substituted dimethylidene acetone derivatives.

We also tried to synthesize the dissymmetrically substituted dimethylidene acetone derivatives using this Ca(OH)_2_-catalyzed methodology (Fig. 4). Initially, the reaction of aldehyde **1a** with a stoichiometric amount of **3a** led to **4a** in 82% yield (Table 4, entry 1). This two-step protocol was then employed to synthesize other dissymmetrically substituted dimethylidene acetone derivatives. Treating aldehydes **1b**-**q** with **3a** at 80 °C in the presence of Ca(OH)_2_ catalyst afforded the corresponding products **4h**-**4n** smoothly (Table 4). The electron-deficient aldehydes led to higher product yield than the electron-riched aldehydes (Table 4, entries 4–5 *vs.* 2–3). Heterocycle-contained aldehydes **1n** and **1p** were also fit for the reaction (Table 4, entries 6–7), but the alkyl substrate **1q** resulted in poor product yield (Table 4, entry 8).

The synthetic efficiency could be improved using a one-pot strategy. Although the product yields of the one-pot synthesis were reduced in some cases (Table 4, entries 1,3–4, 6–8), considering of the loss of the starting materials in **3a** preparation step (Table 2, entry 1, 85% yield), their total yields were higher than that of the multi-step methods (Table 4, entries 1–8).

The role of Ca(OH)_2_ in the reaction was investigated through a series of control experiments. Using 20 mol% of NaOH as base afforded **3a** in only 47% yield (Table 5, entry 1). But with the addition of 10 mol% of the neutral CaCl_2_, the yield of **3a** could be largely enhanced to 78% (Table 5, entry 5). Similar phenomena were also observed in reactions using organic bases (Table 5, entries 3 vs 4). LiOH, an alkali weaker than NaOH, but with a “hard” alkali metal, led to a significantly elevated **3a** yield (Table 5, entries 5 vs 1). These experimental results suggested that the “hard” Ca^2+^ is the key factor for the excellent catalytic performance.

**Figure 5:**
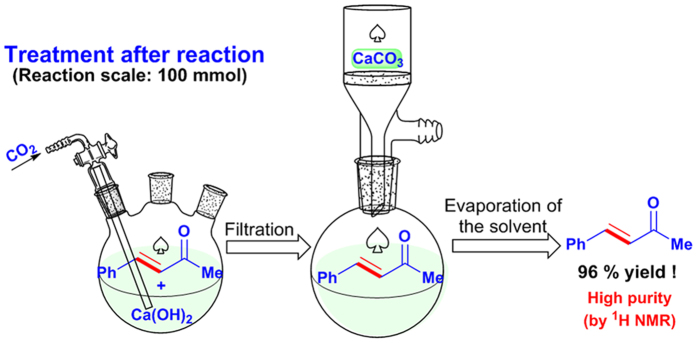
The simple separation procedure for the product.

**Figure 6:**
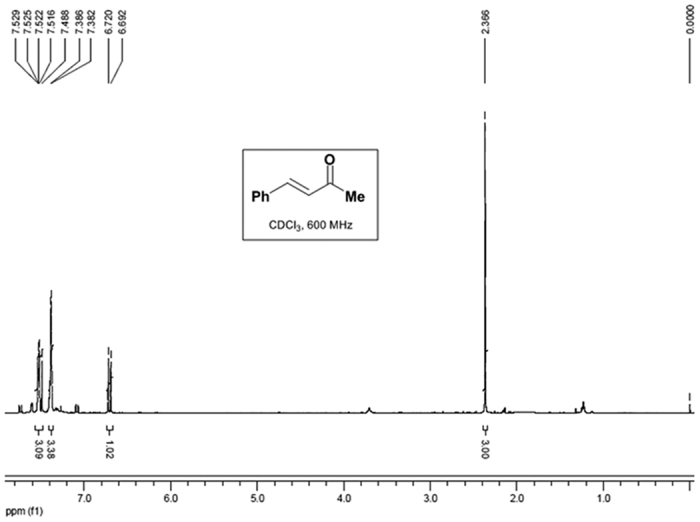
^1^H NMR spectrum of the product **3a** after the evaporation of solvent.

Finally, to examine the practicability of the method, a 100 mmol scale reaction of **1a** with **2a** was performed. After the reaction, calcium was precipitated by CO_2_ and removed through filtration. Evaporation of the solvent directly afforded **3a** in 96% yield with high purity (Fig. 5), as confirmed by its ^1^H NMR spectrum (Fig. 6).

## Conclusion

In conclusion, we have developed a practical synthesis of α,β-unsaturated ketones, including the symmetrically or dissymmetrically substituted dimethylidene acetone derivatives, which are promising compounds for medicinal chemistry. The method employed very low loading (5–10 mol%) Ca(OH)_2_ catalyst, which could be removed by CO_2_. The reactions were performed in cheap and benign dilute aqueous ethanol (20 v/v%). This work shows that Ca(OH)_2_, the abundant but seldom employed base, might find further application in organic synthesis.

## Methods

### General Considerations

Aldehydes were purchased from the reagent merchant. The liquid aldehydes were distilled under vacuum before use, while the solid aldehydes were recrystallized in EtOH-H_2_O under N_2_ before use. Ethanol was analytical pure (AR) and directly used without any special treatment. All reactions were carried out in N_2_ and monitored by TLC. Melting points were measured by WRS-2A digital instrument. IR spectra were measured on Bruker Tensor 27 Infrared spectrometer. ^1^H and ^13^C NMR spectra were recorded on a Bruker Avance 600/400 instrument (600 or 400 MHz for ^1^H and 150 MHz for ^13^C NMR spectroscopy) using CDCl_3_ as the solvent and Me_4_Si as the internal standard. Chemical shifts for ^1^H and ^13^C NMR were referred to internal Me_4_Si (0 ppm) and *J*-values were shown in Hz. Mass spectra were measured on a Shimadzu GCMS-QP2010 Ultra spectrometer (EI).

### Typical procedure for the synthesis of 3

0.1 mmol of Ca(OH)_2_ (7.4 mg) was first added into a reaction tube, which was then charged with N_2_. A solution of 1 mmol of aldehyde **1** and 3 mmol of methyl ketone **2** in EtOH/H_2_O (1 mL, 20 v/v%) was then injected into the reaction tube. The mixture was heat at 50 °C under N_2_ protection and the reaction was monitored by TLC. When the reaction terminated, the solvent was evaporated under vacuum and the residue was purified by preparative TLC (eluent: petroleum ether/EtOAc, 2: 1 for **3m**, 15: 1 for rest compounds).

### Typical procedure for the synthesis of symmetrically substituted dimethylidene acetone derivatives 4

0.1 mmol of Ca(OH)_2_ (7.4 mg) was first added to a reaction tube, which was then charged with N_2_. A solution of 2 mmol of aldehyde **1** and 1 mmol of acetone **2a** in EtOH/H_2_O (1 mL, 20 v/v%) was then injected into the reaction tube, which was then sealed under N_2_ and heat at 80 °C for 48 h. The reaction mixture was isolated by preparative TLC (eluent: petroleum ether/EtOAc, 15: 1).

### Typical procedure for the synthesis of dissymmetrically substituted dimethylidene acetone derivatives 4 (multi-step)

0.1 mmol of Ca(OH)_2_ (7.4 mg) and 1 mmol of **3a** were added into a reaction tube, which was then charged with N_2_. A solution of 1 mmol of aldehyde **1** in EtOH/H_2_O (1 mL, 20 v/v%) was then injected into the reaction tube. The mixture was heat at 80 °C under N_2_ for 48 h and then isolated by preparative TLC (eluent: petroleum ether/EtOAc, 15: 1).

### Typical procedure for the synthesis of dissymmetrically substituted dimethylidene acetone derivatives 4 (one-pot)

0.1 mmol of Ca(OH)_2_ (7.4 mg) was first added into a 10 mL round bottom flask, which was then charged with N_2_. A solution of 1 mmol of aldehyde **1** and 3 mmol of methyl ketone **2** in EtOH/H_2_O (1 mL, 20 v/v%) was then injected into the reaction tube. The mixture was heat at 50 °C under N_2_ protection. After 10 h, the solvent was evaporated under vacuum and another solution of 1 mmol of aldehyde **1** in EtOH/H_2_O (1 mL, 20 v/v%) was then injected. The mixture was heat at 80 °C under N_2_ for 48 h and isolated by preparative TLC (eluent: petroleum ether/EtOAc, 15:1).

### Procedure for the large-scale reaction

To a 250 mL three-neck flask, 10 mmol of Ca(OH)_2_ (0.74 g) was added. The flask was then charged with N_2_. A solution of 100 mmol of benzaldehyde **1a** and 300 mmol of acetone **2a** in 100 mL EtOH/H_2_O (20 v/v%) was then injected. The mixture was stirred at 50 °C under N_2_ protection for 10 h and then cooled to room temperature. CO_2_ was then charged into the liquid and the pH was controlled to 7.0 (monitored by a pH meter). The precipitated CaCO_3_ was removed by filtration and the filtrate was collected. After the evaporation of the solvent, 14.0 g of the product **3a** was obtained in the excellent 96% yield. The product was directly sent to ^1^H NMR analysis without any further purification and the results in Fig. 2 confirmed its high purity.

## Additional Information

**How to cite this article**: Yu, L. *et al*. Ca(OH)_2_-Catalyzed Condensation of Aldehydes with Methyl ketones in Dilute Aqueous Ethanol: A Comprehensive Access to a,β-Unsaturated Ketones. *Sci. Rep.* **6**, 30432; doi: 10.1038/srep30432 (2016).

## Supplementary Material

Supplementary Information

## Figures and Tables

**Table 1:** Optimization of the reaction conditions^a^.

Entry	EtOH/H_2_O^b^	t/h	3a/%^c^
1	100:0	20	68
2	80:20	16	69
3	50:50	14	84
4	20:80	10	85
5	10:90	24	79
6	5:95	36	0
7	0:100	36	0

^a^Reaction conditions: 1 mmol **1a**, 3 mmol **2a**, 0.1 mmol Ca(OH)_2_ and 1 mL of solvent were employed.

^b^Volume ratio of EtOH with water.

^c^Isolated yields of **3a** based on **1a**.

**Table 2:** Substrate extension of the Ca(OH)_2_-catalyzed Claisen-Schmidt condensation^a^.

Entry	1: R^1^; 2: R^2^	3: t/h^b^, yield/%^c^
1	**1a**: Ph; **2a**: Me	**3a**: 10 h, 85
2	**1b**: 4-MeC_6_H_4_; **2a**: Me	**3b**: 36 h, 83
3	**1c**: 3-MeC_6_H_4_; **2a**: Me	**3c**: 24 h, 67
4	**1d**: 2-MeC_6_H_4_; **2a**: Me	**3d**: 28 h, 60
5	**1e**: 4-MeOC_6_H_4_; **2a**: Me	**3e**: 48 h, 61d,^e^
6	**1f**: 4-FC_6_H_4_; **2a**: Me	**3f**: 9h, 78
7	**1g**: 4-ClC_6_H_4_; **2a**: Me	**3g**: 10 h, 72
8	**1h**: 2-ClC_6_H_4_; **2a**: Me	**3h**: 8 h, 52^d^
9	**1i**: 4-BrC_6_H_4_; **2a**: Me	**3i**: 10 h, 71
10	**1j**: 4-CF_3_C_6_H_4_; **2a**: Me	**3j**: 24 h, 72^d^
11	**1k**:4-NO_2_C_6_H_4_; **2a**: Me	**3k**: 8 h, 50^d^
12	**1l**:1-C_10_H_7_; **2a**: Me	**3l**: 36 h, 58
13	**1m**: 2-C_5_H_4_N-; **2a**: Me	**3m**: 24 h, 55d,f,^g^
14	**1n**: 2-C_4_H_3_S-; **2a**: Me	**3n**: 10 h, 90
15	**1o**: *E*-PhCH = CH-; **2a**: Me	**3o**: 30 h, 91
16	**1p**: *c*-C_6_H_11_; **2a**: Me	**3p**: 48 h, 30h,^i^
17	**1a**: Ph; **2b**: Ph	**3q**: 18 h, 71
18	**1a**: Ph; **2c**: 4-MeC_6_H_4_	**3r**: 48 h, 61^h^
19	**1a**: Ph; **2d**: 4-ClC_6_H_4_	**3s**: 40 h, 68
20	**1a**: Ph; **2e**: *n*-Bu	**3t**: 48 h, 54h,^i^
21	**1a**: Ph; **2f**: *i*-Pr	**3u**: 48 h, 60^h^

^a^Reaction conditions: without special instructions, 1 mmol of **1**, 3 mmol of **2** and 0.1 mmol Ca(OH)_2_ were heat in 1 mL of EtOH/H_2_O (20 v/v%) at 50 °C.

^b^Reactions monitored by TLC (eluent: petroleum ether/EtOAc 9:1).

^c^Isolated yields based on **1**.

^d^Reaction performed at room temperature (ca. 25 °C).

^e^10 mmol of acetone was employed.

^f^Ca(OH)_2_ loading was reduced to 5 mol%.

^g^1 mL of acetone was employed.

^h^Reaction uncompleted.

^i^Reaction performed at 120 °C in a pressure tube.

**Table 3:** Synthesis of symmetrically substituted dimethylidene acetone derivatives^a^.

Entry	1: R	4: yield/%^b^
1	**1a**: Ph	**4a**: 84
2	**1b**: 4-MeC_6_H_4_	**4b**: 39
3	**1e**: 4-MeOC_6_H_4_	**4c**: 31
4	**1f**: 4-FC_6_H_4_	**4d**: 78
5	**1j**: 4-CF_3_C_6_H_4_	**4e**: 92
6	**1n**: 2-C_4_H_3_S-	**4f**: 62
7	**1p**: 2-C_4_H_3_O-	**4g**: 80

^a^Reaction conditions: 2 mmol **1**, 1 mmol **2** and 0.1 mmol Ca(OH)_2_ were heat in 1 mL of EtOH/H_2_O (20 v/v%) at 80 °C.

^b^Isolated yields based on **2a**.

**Table 4:** Synthesis of dissymmetrically substituted dimethylidene acetone derivatives^a^.

Entry	1: R	4: yield/%^b^	
		Multi-step^c^	One-pot
1	**1a**: Ph	**4a**: 82 (70)	**4a**: 71
2	**1b**: 4-MeC_6_H_4_	**4h**: 62 (53)	**4h**: 68
3	**1e**: 4-MeOC_6_H_4_	**4i**: 52 (44)	**4i**: 46
4	**1f**: 4-FC_6_H_4_	**4j**: 81 (69)	**4j**: 75
5	**1j**: 4-CF_3_C_6_H_4_	**4k**: 90 (77)	**4k**: 92
6	**1n**: 2-C_4_H_3_S-	**4l**: 68 (58)	**4l**: 61
7	**1p**: 2-C_4_H_3_O-	**4m**: 72 (61)	**4m**: 80
8	**1q**: *c*-C_6_H_11_-	**4n**: 24 (20)	**4n**: 21

^a^Reactions were performed in 1 mL of EtOH/H_2_O (20 v/v%) catalysed by 0.1 mmol of Ca(OH)_2_.

^b^Isolated yields. ^c^Total yields from 1a and 2a in parentheses (×85%).

**Table 5:** Control experiments^a^.

Entry	Cat. (mol%)	3a yield/%^b^
1	NaOH (20)	47
2	NaOH (20) + CaCl_2_ (10)	78
3	Et_3_N (20)	35
4	Et_3_N (20) + CaCl_2_ (10)	53
5	LiOH (20)	71

^a^1 mmol **1a**, 3 mmol **2a**, and 1 mL of solvent were employed.

^b^Molar ration based on **1a** in parentheses.

^c^Isolated yields based on **1a**.
