# Supplementary material for: Ca(OH)2-Catalyzed Condensation of Aldehydes with Methyl ketones in Dilute Aqueous Ethanol: A Comprehensive Access to α,β-Unsaturated Ketones
Source: Sci Rep. 2016 Jul 22;6:30432. doi: 10.1038/srep30432 (PMC4957220; doi:10.1038/srep30432)
Supplement: Supplementary Information [file srep30432-s1.pdf]

Ca(OH)$_2$-catalyzed condensation of aldehydes with methyl ketones in dilute aqueous ethanol: A comprehensive access to α,β-unsaturated ketones.

Lei Yu,*[1] Mengting Han,[1] Jie Luan,[1] Lin Xu,[1,2] Yuanhua Ding[1] and Qing Xu[1]

[1] Jiangsu Co-innovation Center for Prevention and Control of Important Animal Infectious Diseases and Zoonoses, Jiangsu Key Laboratory of Zoonosis, School of Chemistry and Chemical Engineering, Yangzhou University, Yangzhou, Jiangsu 225002, China. Email: yulei@yzu.edu.cn; Fax: (+)-86-514-87975244; Tel: (+)-86-136-65295901.

[2] Jiangsu Yangnong Chemical Group Co. Ltd., Yangzhou, Jiangsu, 225009, China.

Table of Contents

Detailed Condition Optimizations……………………………………………………S2
Detailed Application Scope Examinations……………………………………...S3
NMR Spectra of the products…………………………………………….……..S5

Detailed Condition Optimizations

Table S1. Detailed Condition Optimization Table.[a]

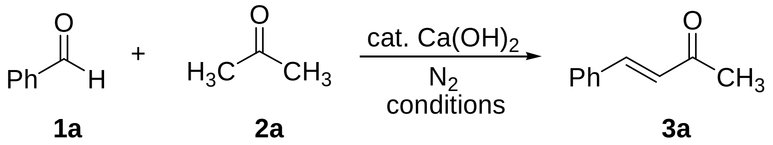

run	Solvent (mL)	cat./%[b]	*T*/°C	t/h[c]	**3a** yield/%[d]
1	EtOH (1)	10	30	36	59
2	EtOH (1)	20	30	28	52
3	EtOH (1)	10	50	20	68
4	Acetone (1)	10	30	36	NR[e]
5	Acetone (1)	20	30	36	NR[e]
6	Acetone (1)	10	50	36	NR[e]
7	Acetone (1)	20	50	36	NR[e]
8	Acetone (1)	20	80	36[f]	58
9	Acetone (1)+EtOH (0.02)	20	50	36[f]	10
10	Acetone (1)+EtOH (0.1)	20	50	36[f]	31
11	Acetone (1)+EtOH (0.2)	20	50	36[f]	42
12	H_2O (1)	10	50	36	NR[e]
13	H_2O (0.95)+EtOH (0.05)	10	50	36	NR[e]
14	H_2O (0.9)+EtOH (0.1)	10	50	24	79
15	H_2O (0.8)+EtOH (0.2)	10	50	10	85
16	H_2O (0.5)+EtOH (0.5)	10	50	14	84
17	H_2O (0.2)+EtOH (0.8)	10	50	16	69

[a] 1 mmol of **1a** and 3 mmol of **2a** were employed. [b] Cat. loading based on **1a**. [c] Reactions monitored by TLC. [d] Isolated yields based on **1a**. [e] No **3a** detected. [f] Reaction not completed.

Detailed Application Scope Examinations

Table S2 Substrate extension of the $Ca(OH)_2$-catalyzed Claisen-Schmidt condensation[a]

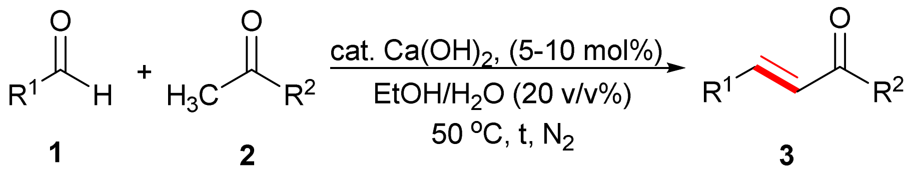

Entry	**1**: R^1; **2**: R^2	**3**: t/h,[b] yield/%[c]
1	**1a**: Ph; **2a**: Me	**3a**: 10 h, 85
2	**1b**: 4-MeC_6H_4; **2a**: Me	**3b**: 36 h, 83
3	**1c**: 3-MeC_6H_4; **2a**: Me	**3c**: 24 h, 67
4	**1d**: 2-MeC_6H_4; **2a**: Me	**3d**: 28 h, 60
5	**1e**: 4-$MeOC_6H_4$; **2a**: Me	**3e**: 48 h, 61[a,e]
6	**1f**: 4-FC_6H_4; **2a**: Me	**3f**: 9h, 78
7	**1g**: 4-ClC_6H_4; **2a**: Me	**3g**: 10 h, 72
8	**1h**: 2-ClC_6H_4; **2a**: Me	**3h**: 6 h, 40
9		**3h**: 8 h, **52**[a]
10	**1i**: 4-BrC_6H_4; **2a**: Me	**3i**: 10 h, 71
11	**1j**: 4-$CF_3C_6H_4$; **2a**: Me	**3j**: 4 h, 25
12		**3j**: 24 h, **72**[a]
13	**1k**:4-$NO_2C_6H_4$; **2a**: Me	**3k**: 6 h, 38
14		**3k**: 8 h, **50**[a]
15	**1l**:1-$C_{10}H_7$; **2a**: Me	**3l**: 36 h, 58
16	**1m**: 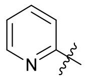; **2a**: Me	**3m**: 8 h, 19
17		**3m**: 16 h, 20[d]
18		**3m**: 20 h, 21[d,f]
19		**3m**: 24 h, **55**[d,f,g]
20	**1n**: 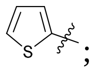; **2a**: Me	**3n:** 10 h, 90
21	**1o**: 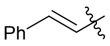 ; **2a**: Me	**3o**: 30 h, 91
22	**1p**: 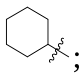; **2a**: Me	**3p**: 48 h, 30[i]
23	**1a**: Ph; **2b**: Ph	**3q**: 18 h, 71
24	**1a**: Ph; **2c**: 4-MeC_6H_4	**3r**: 48 h, 61[h]
25	**1a**: Ph; **2d**: 4-ClC_6H_4	**3s**: 40 h, 68
26	**1a**: Ph; **2e**: *n*-Bu	**3t**: 48 h, 54[h,ı]
27	**1a**: Ph; **2f**: *i*-Pr	**3u**: 48 h, 60[h]

[a] Without special instructions, 1 mmol 1, 3 mmol 2 and 0.1 mmol $Ca(OH)_2$ were heat in 1 mL of EtOH/H_2O (20 v/v%) at 50 °C. [b] Reactions monitored by TLC (eluent :

petroleum ether/EtOAc 9:1). [c] Isolated yields based on 1. [d] Reaction performed at room temperature (*ca.*25 ^{o}C). [e] 10 mmol of acetone was employed. [f] $Ca(OH)_2$ loading was reduced to 5 mol %. [g] 1 mL of acetone was employed. [h] Reaction uncompleted. [i] Reaction performed at 120 ^{o}C.

NMR Spectra of the products

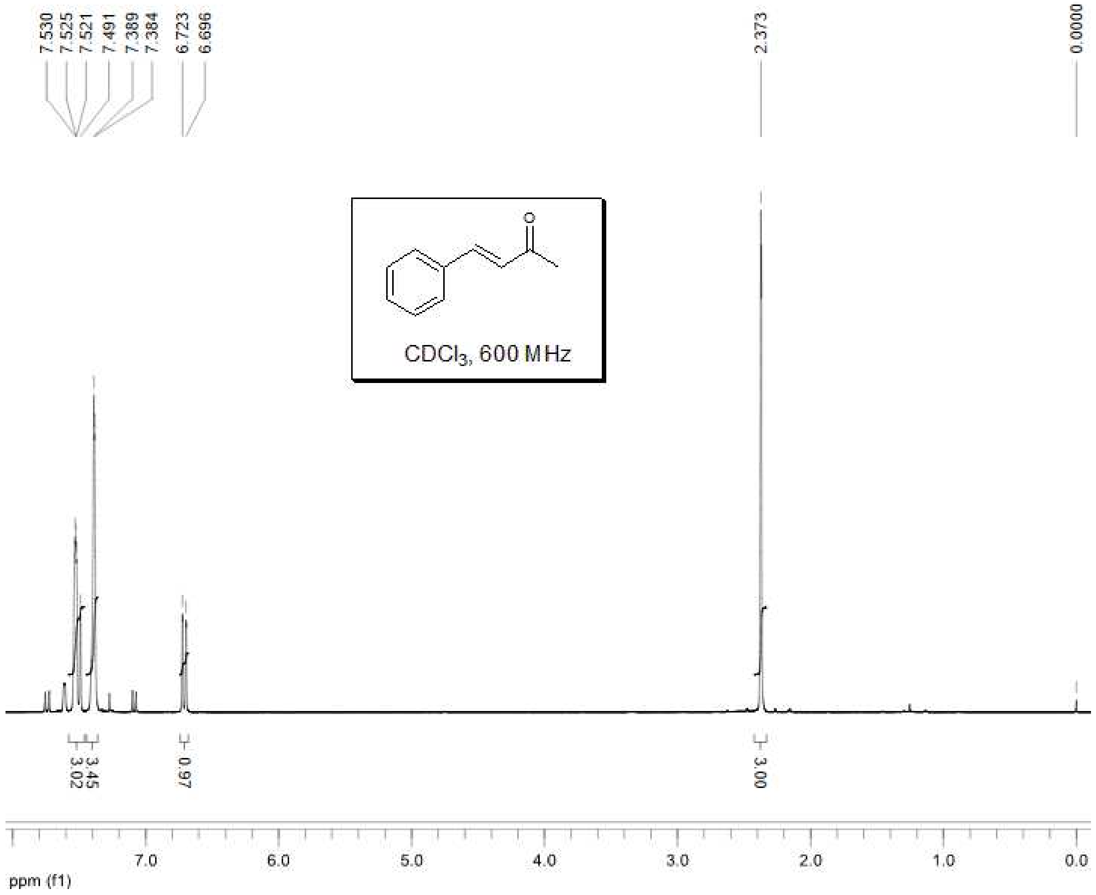

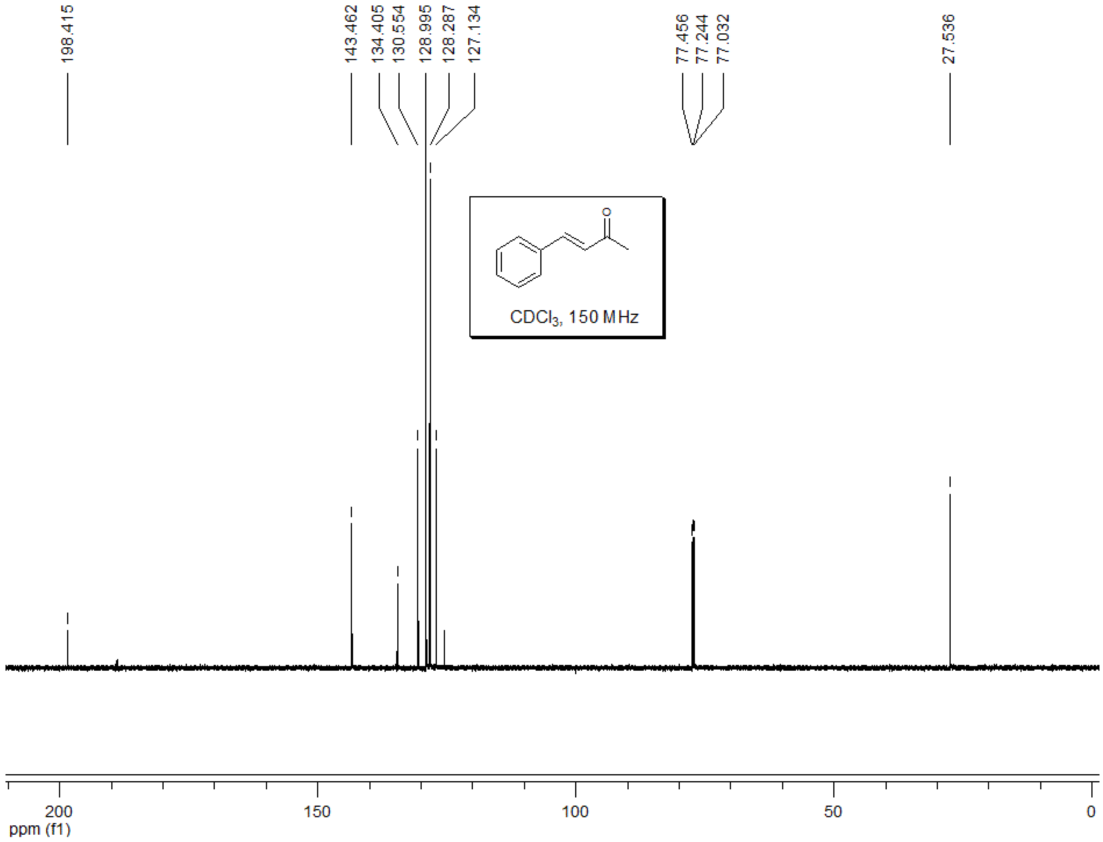

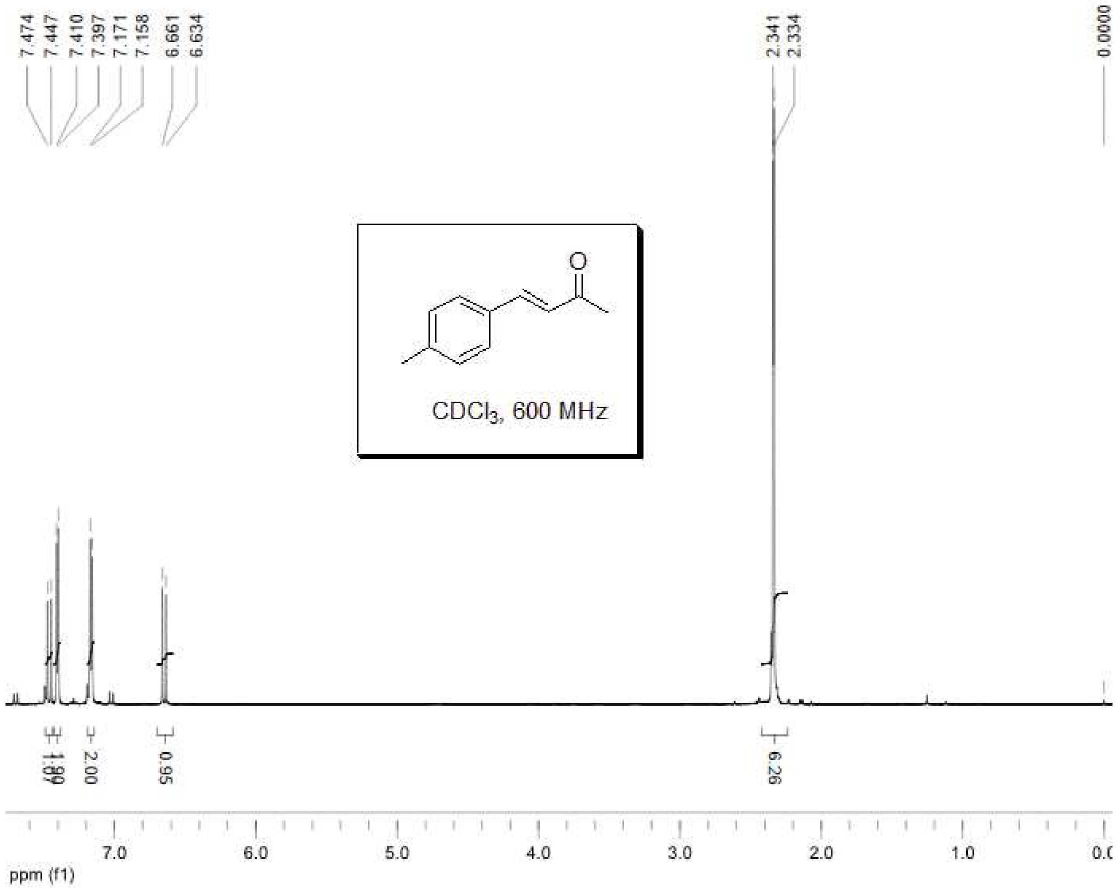

7.474
7.447
7.410
7.397
7.171
7.158
6.661
6.634
2.341
2.334
0.0000
CDCl3, 600 MHz
2.00
0.95
6.26
7.0
6.0
5.0
4.0
3.0
2.0
1.0
ppm (f1)

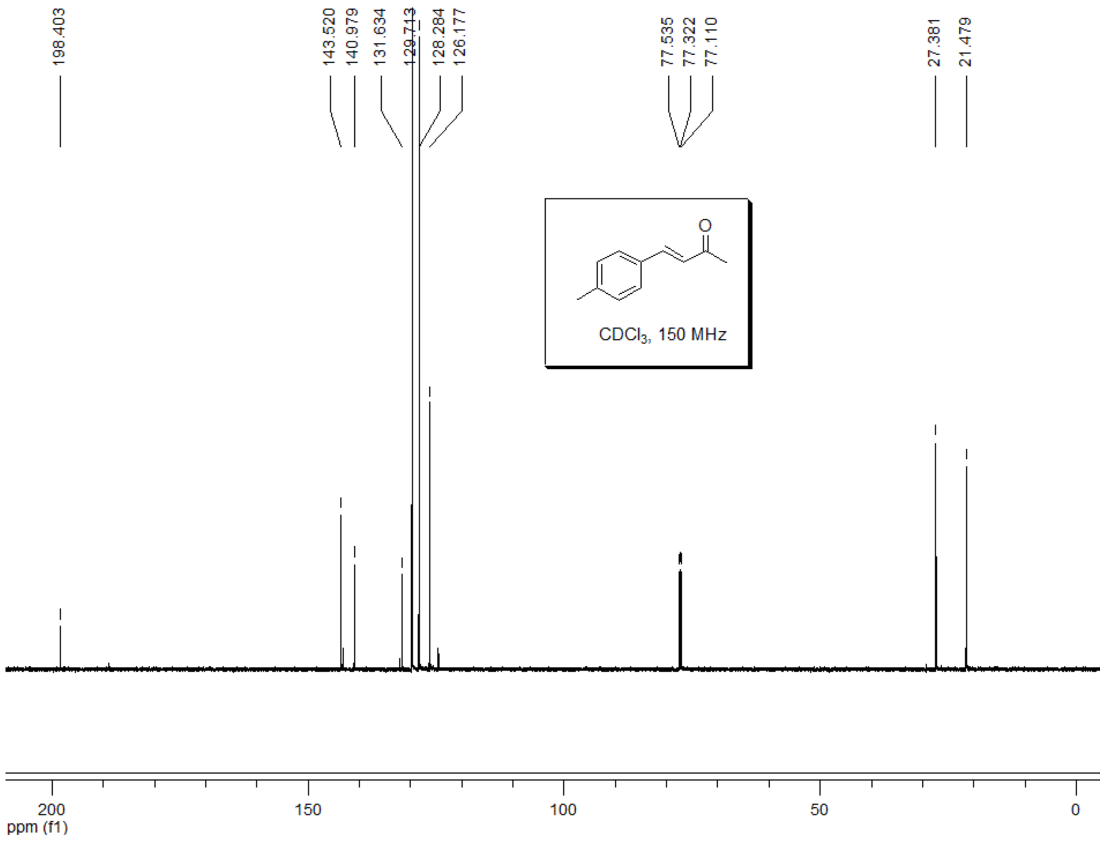

198.403
143.520
140.979
131.634
129.713
128.284
126.177
77.535
77.322
77.110
27.381
21.479
CDCl3, 150 MHz
200
150
100
50
0
ppm (f1)

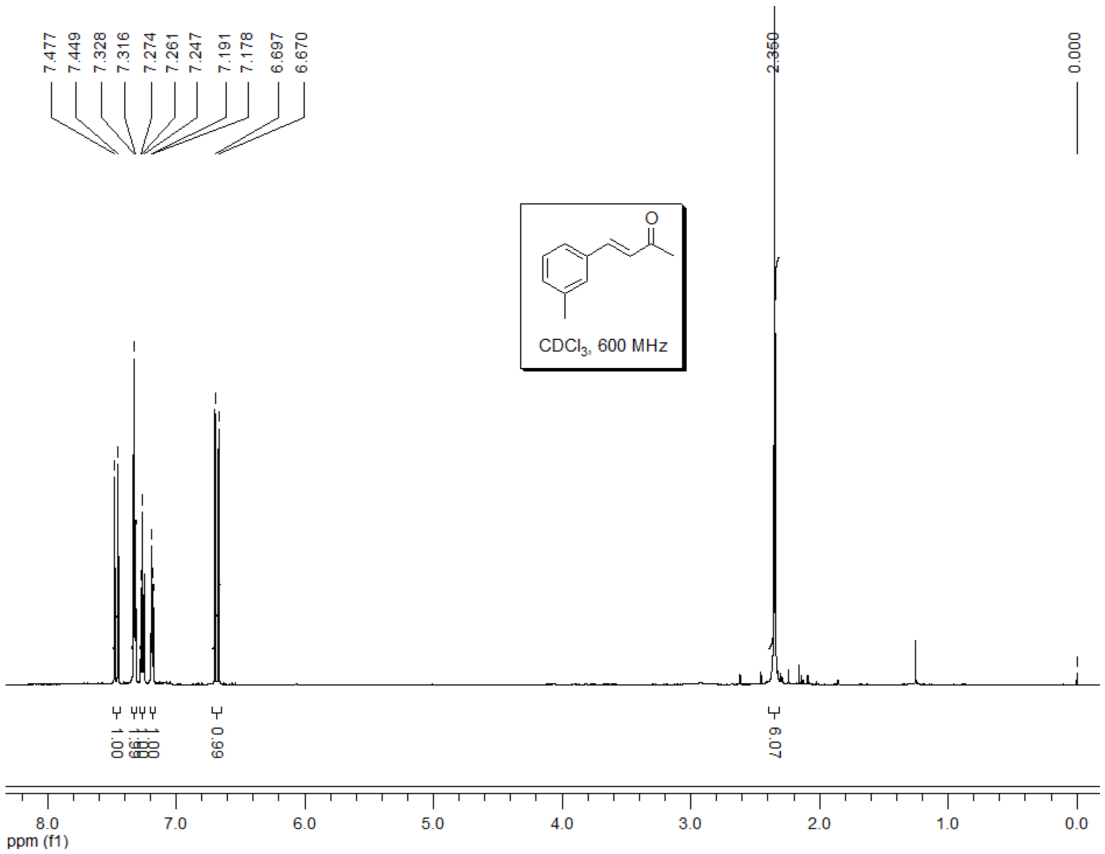
7.477
7.449
7.328
7.316
7.274
7.261
7.247
7.191
7.178
6.697
6.670
2.350
0.000
CDCl3, 600 MHz
1.00
0.99
6.07
8.0
7.0
6.0
5.0
4.0
3.0
2.0
1.0
0.0
ppm (f1)

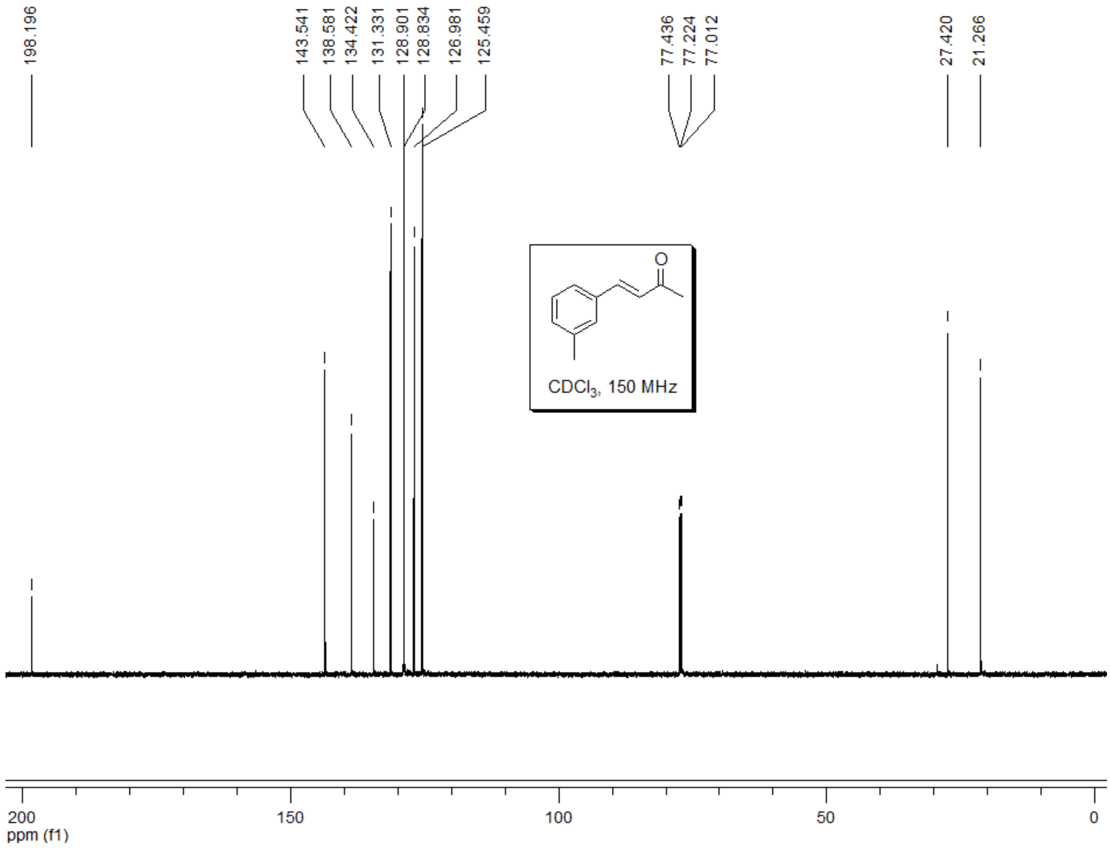
198.196
143.541
138.581
134.422
131.331
128.901
128.834
126.981
125.459
77.436
77.224
77.012
27.420
21.266
CDCl3, 150 MHz
200
150
100
50
0
ppm (f1)

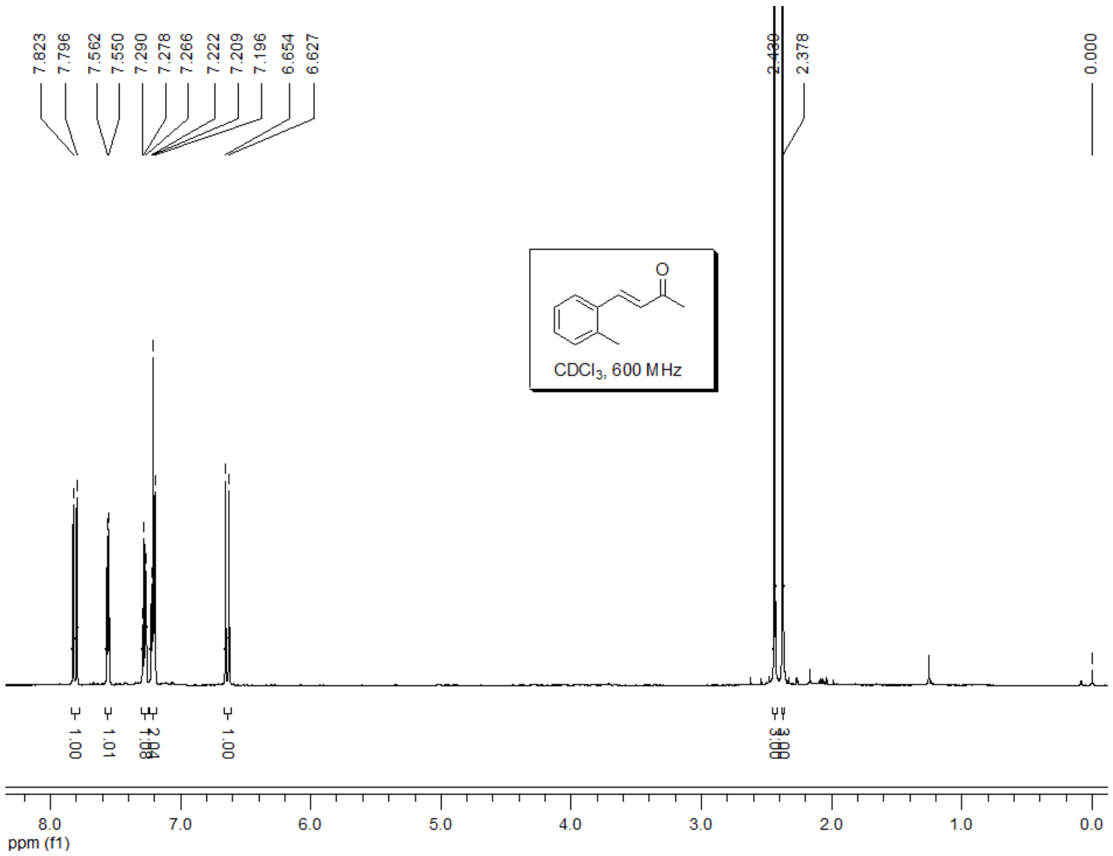
7.823
7.796
7.562
7.550
7.290
7.278
7.266
7.222
7.209
7.196
6.654
6.627
2.430
2.378
0.000
O
CDCl$_3$, 600 MHz
1.00
1.01
1.00
3.00
ppm (f1)

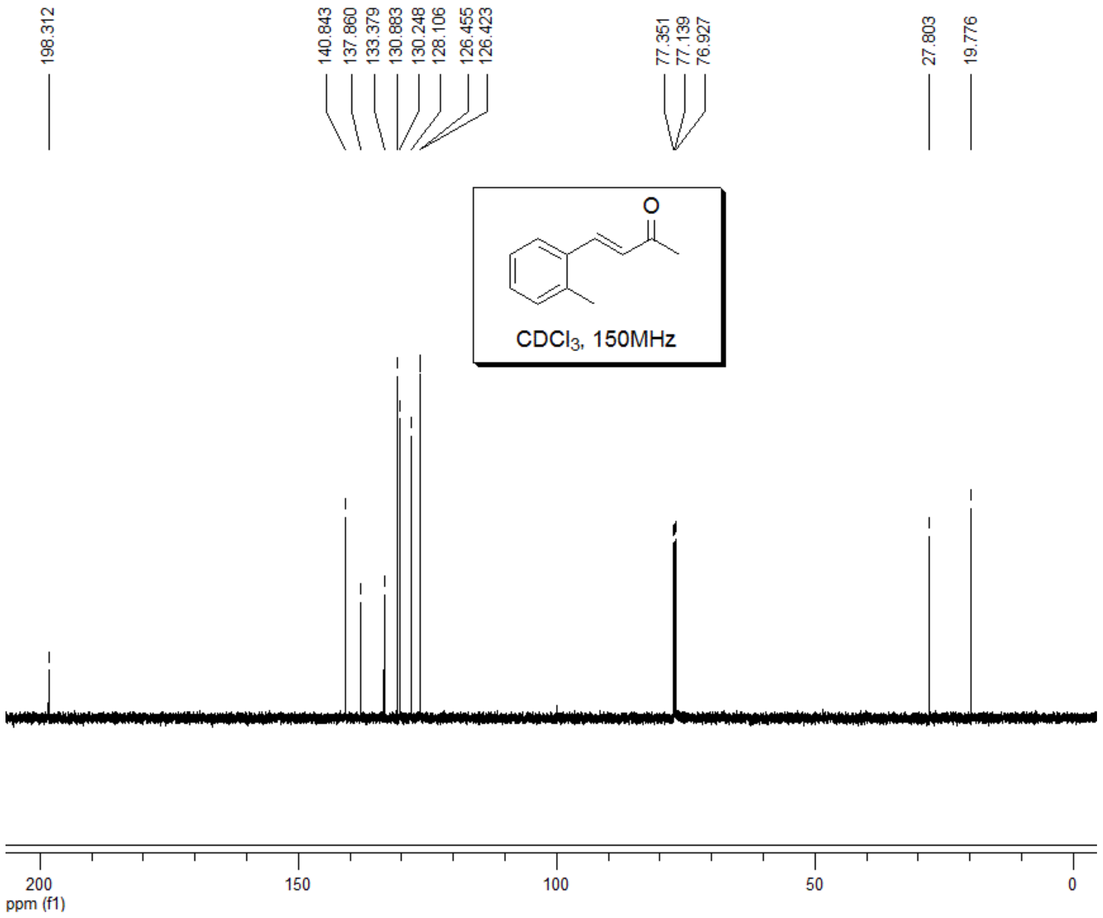
198.312
140.843
137.860
133.379
130.883
130.248
128.106
126.455
126.423
77.351
77.139
76.927
27.803
19.776
O
CDCl$_3$, 150MHz
ppm (f1)

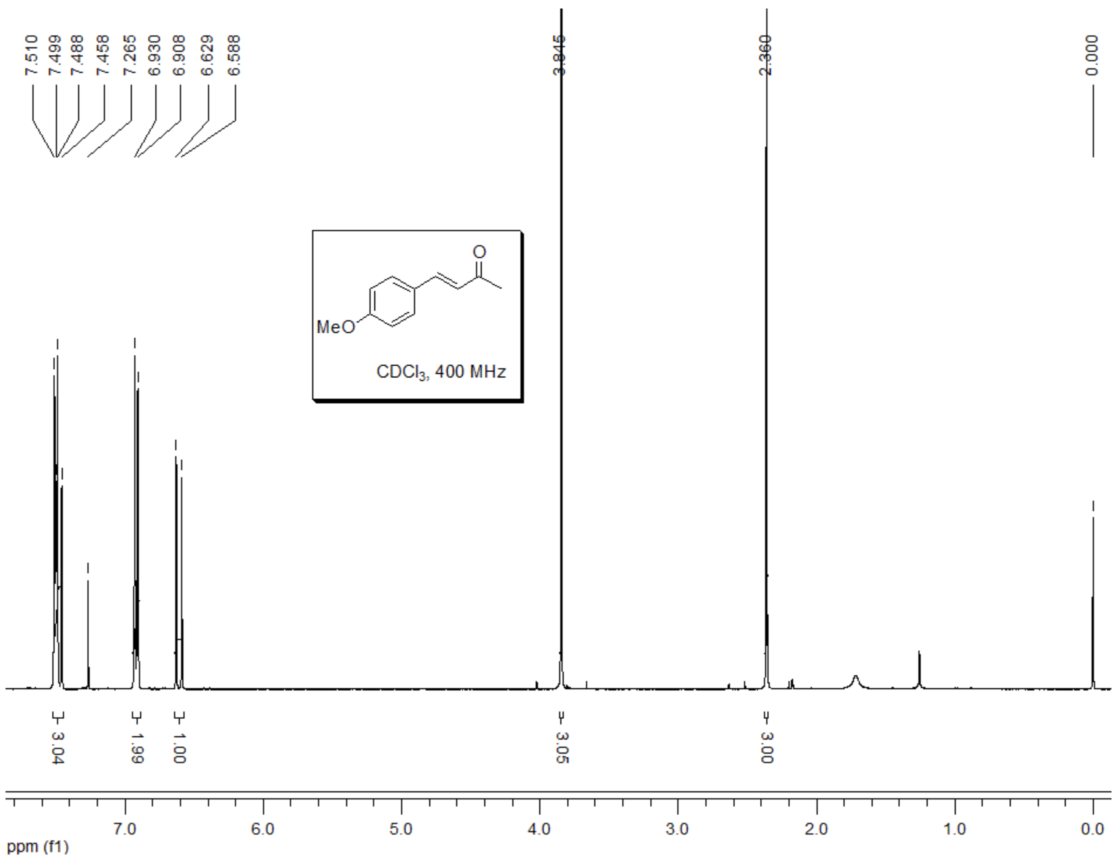
7.510
7.499
7.488
7.458
7.265
6.930
6.908
6.629
6.588
3.845
2.360
0.000
MeO
O
CDCl3, 400 MHz
3.04
1.99
1.00
3.05
3.00
7.0
6.0
5.0
4.0
3.0
2.0
1.0
0.0
ppm (f1)

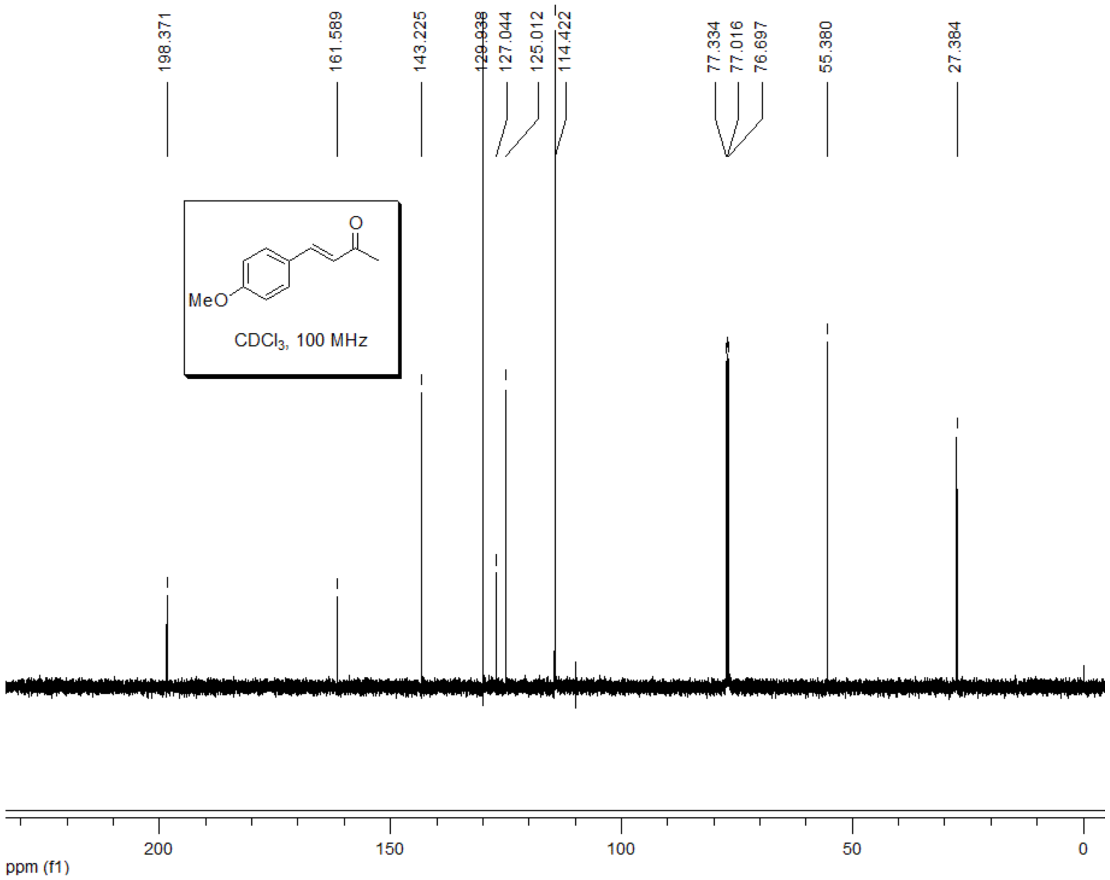
198.371
161.589
143.225
129.938
127.044
125.012
114.422
77.334
77.016
76.697
55.380
27.384
MeO
O
CDCl3, 100 MHz
200
150
100
50
0
ppm (f1)

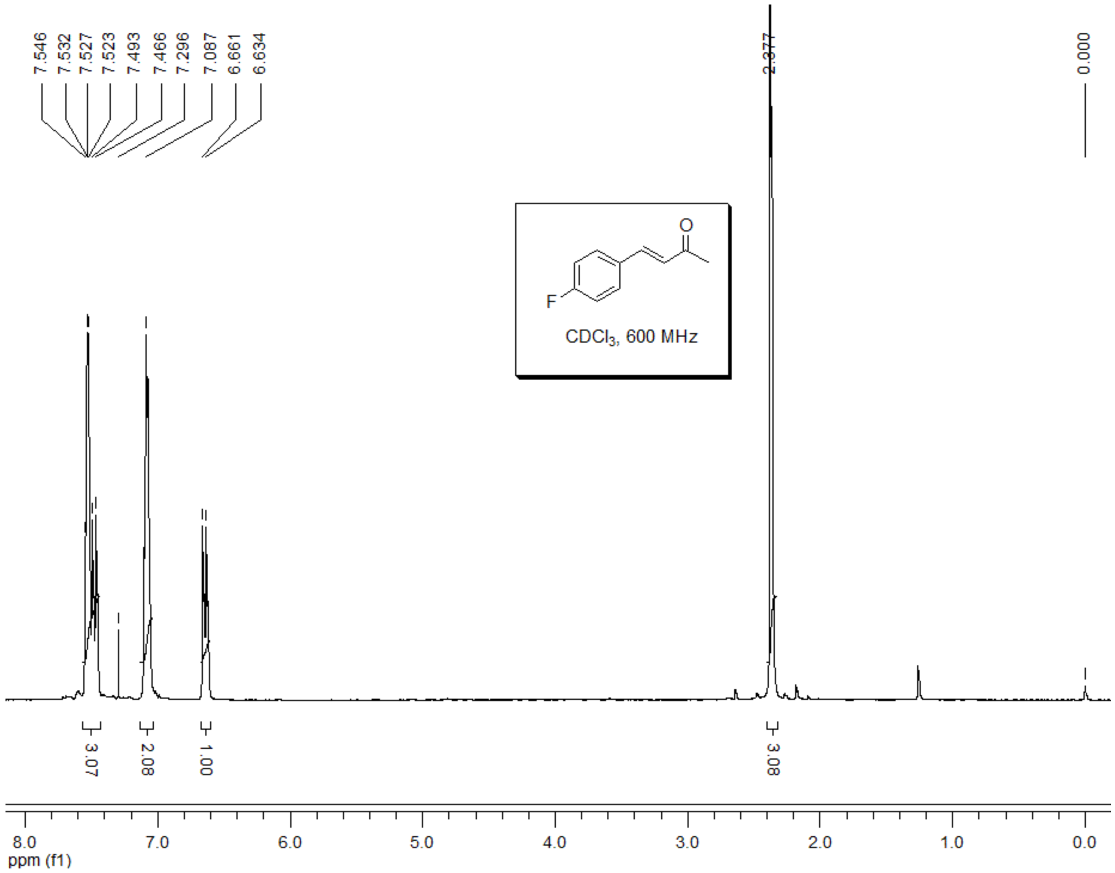
7.546
7.532
7.527
7.523
7.493
7.466
7.296
7.087
6.661
6.634
2.377
0.000
O
F
CDCl3, 600 MHz
3.07
2.08
1.00
3.08
8.0
7.0
6.0
5.0
4.0
3.0
2.0
1.0
0.0
ppm (f1)

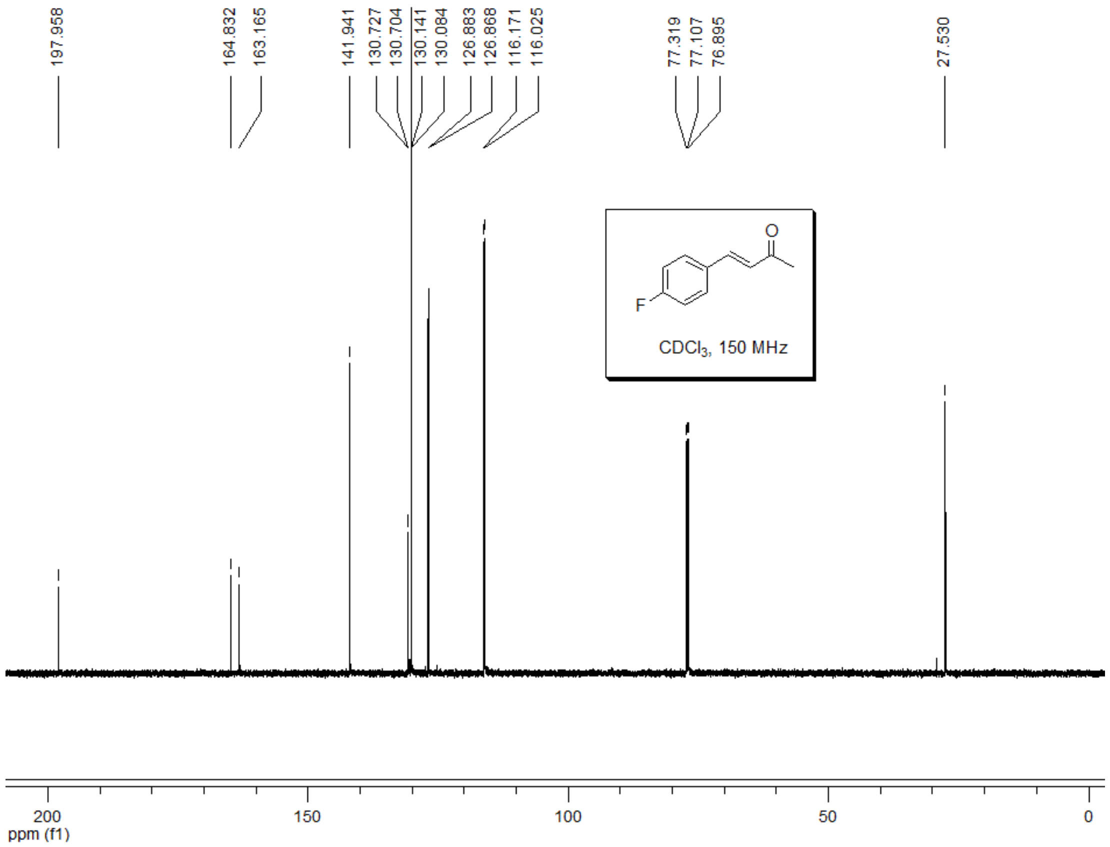
197.958
164.832
163.165
141.941
130.727
130.704
130.141
130.084
126.883
126.868
116.171
116.025
77.319
77.107
76.895
27.530
O
F
CDCl3, 150 MHz
200
150
100
50
0
ppm (f1)

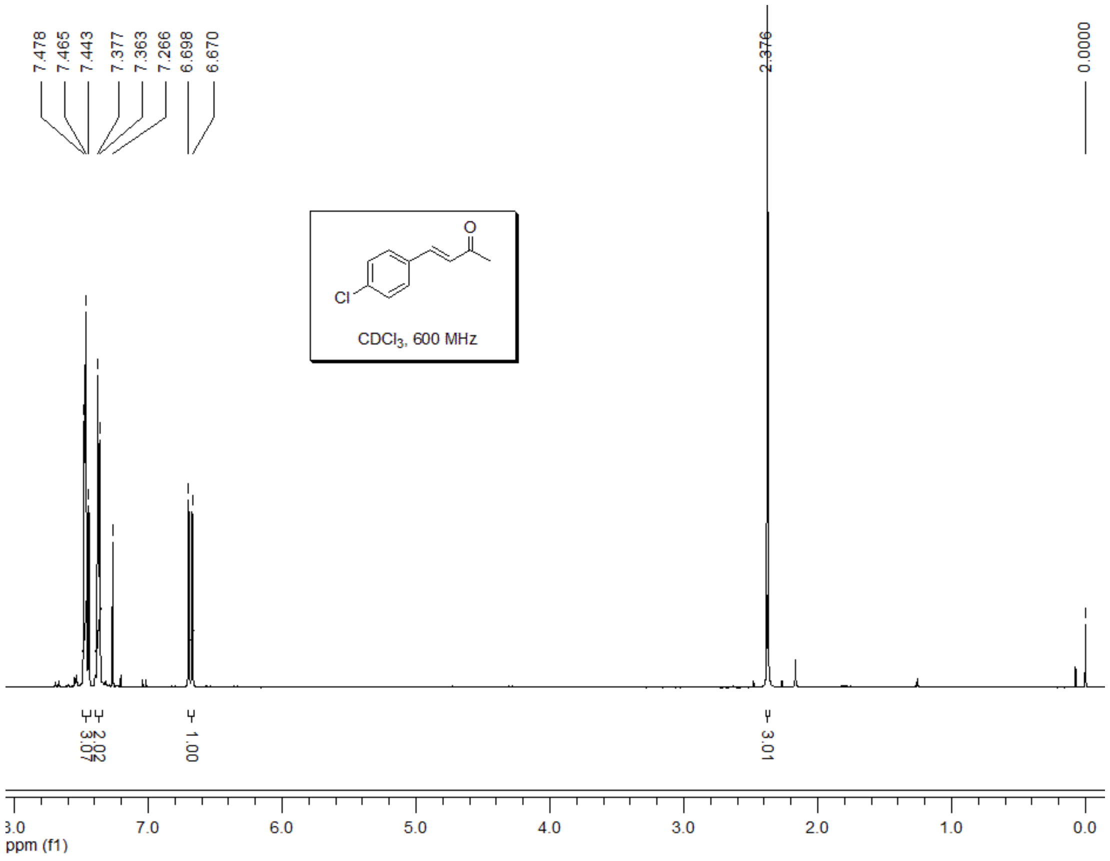
7.478
7.465
7.443
7.377
7.363
7.266
6.698
6.670
2.376
0.0000
O
Cl
CDCl3, 600 MHz
3.07
2.02
1.00
3.01
7.0
6.0
5.0
4.0
3.0
2.0
1.0
0.0
ppm (f1)

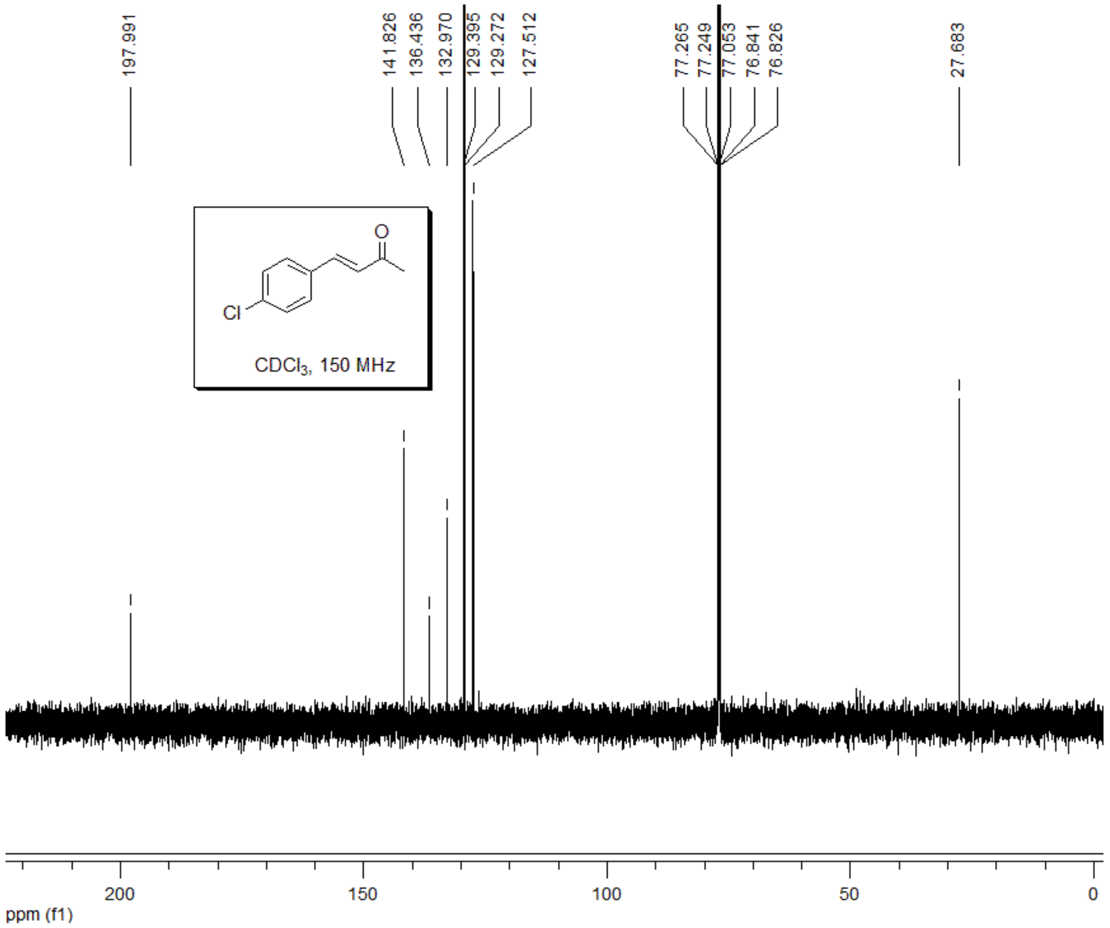
197.991
141.826
136.436
132.970
129.395
129.272
127.512
77.265
77.249
77.053
76.841
76.826
27.683
Cl
O
CDCl3, 150 MHz
200
150
100
50
0
ppm (f1)

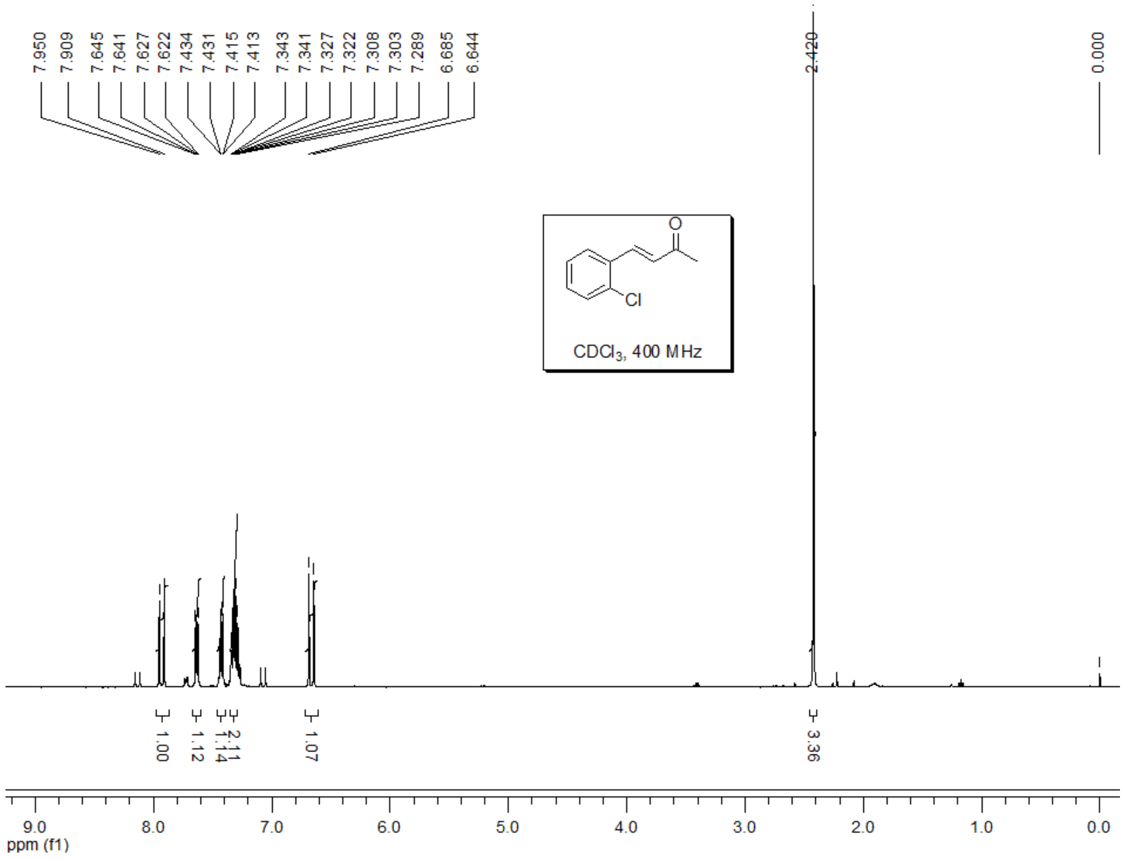
7.950
7.909
7.645
7.641
7.627
7.622
7.434
7.431
7.415
7.413
7.343
7.341
7.327
7.322
7.308
7.303
7.289
6.685
6.644
2.420
0.000
O
Cl
CDCl3, 400 MHz
1.00
1.12
1.14
2.11
1.07
3.36
9.0
8.0
7.0
6.0
5.0
4.0
3.0
2.0
1.0
0.0
ppm (f1)

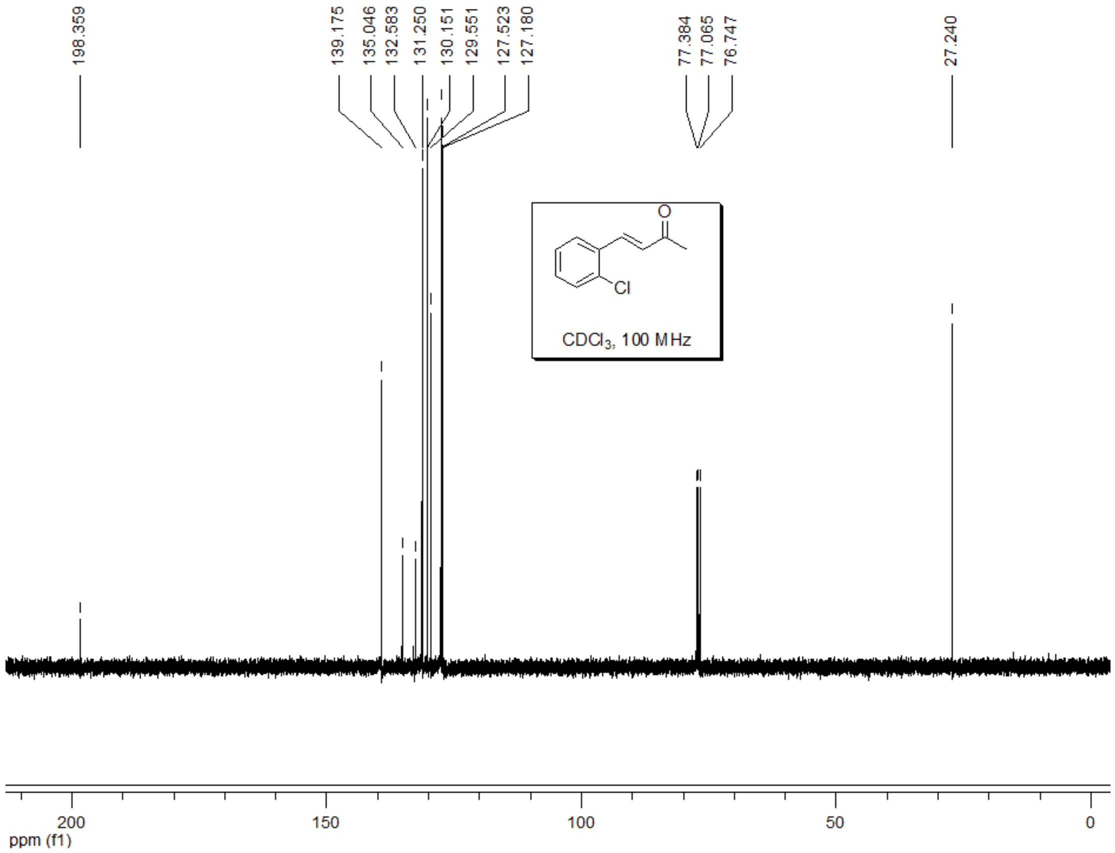
198.359
139.175
135.046
132.583
131.250
130.151
129.551
127.523
127.180
77.384
77.065
76.747
27.240
O
Cl
CDCl3, 100 MHz
200
150
100
50
0
ppm (f1)

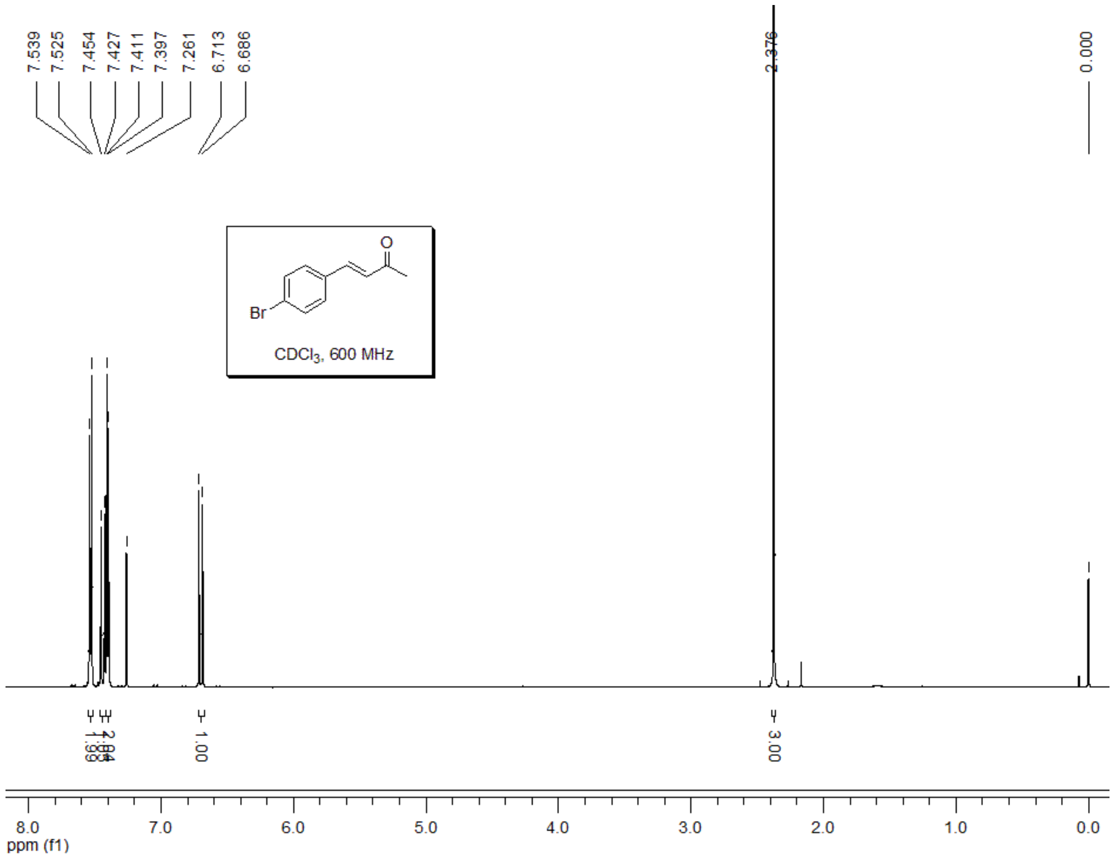
7.539
7.525
7.454
7.427
7.411
7.397
7.261
6.713
6.686
2.376
0.000
O
Br
CDCl3, 600 MHz
1.00
3.00
8.0
7.0
6.0
5.0
4.0
3.0
2.0
1.0
0.0
ppm (f1)

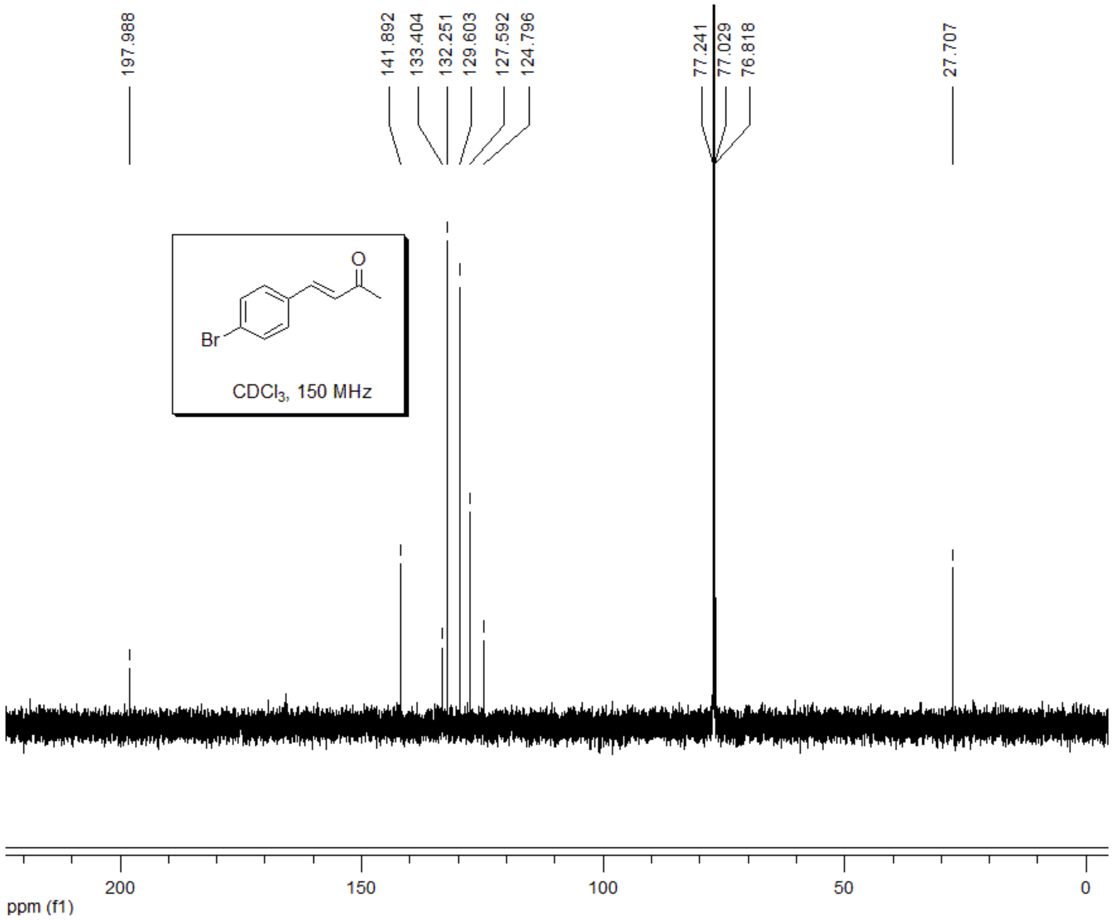
197.988
141.892
133.404
132.251
129.603
127.592
124.796
77.241
77.029
76.818
27.707
O
Br
CDCl3, 150 MHz
200
150
100
50
0
ppm (f1)

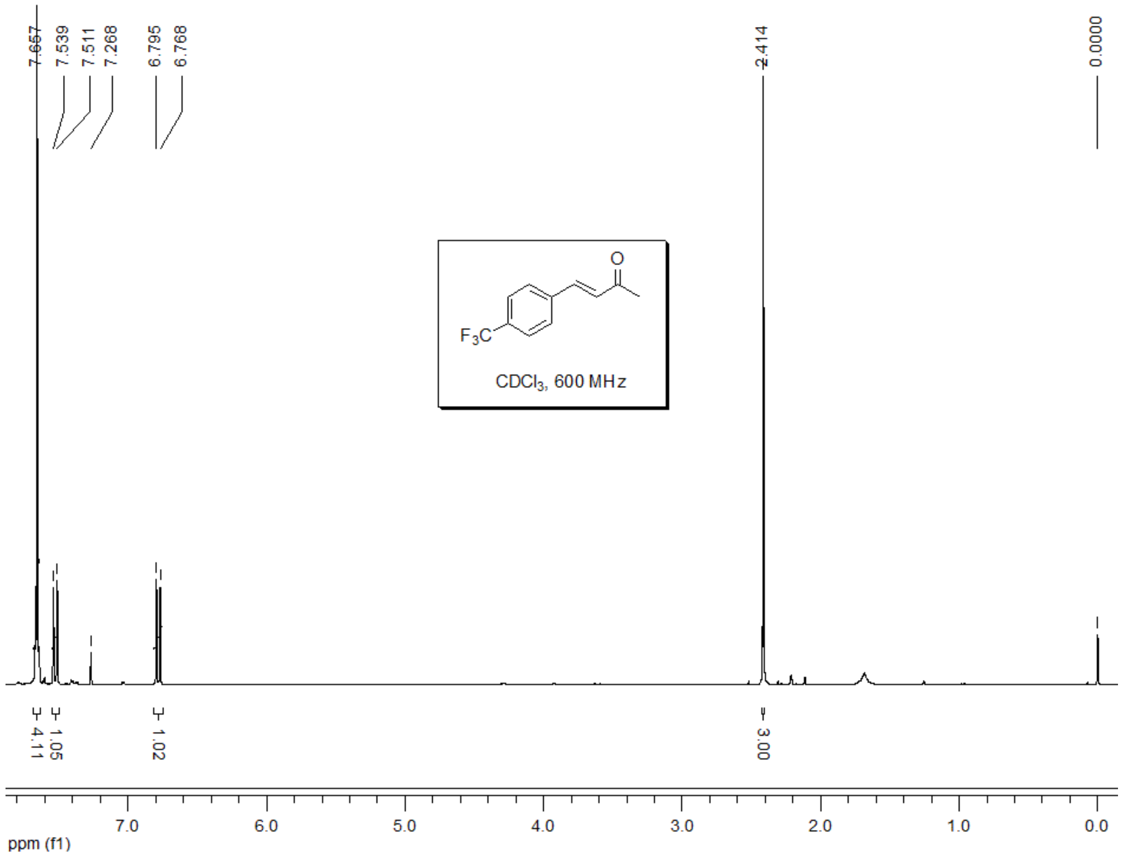
7.657
7.539
7.511
7.268
6.795
6.768
2.414
0.0000
O
F3C
CDCl3, 600 MHz
4.11
1.05
1.02
3.00
7.0
6.0
5.0
4.0
3.0
2.0
1.0
0.0
ppm (f1)

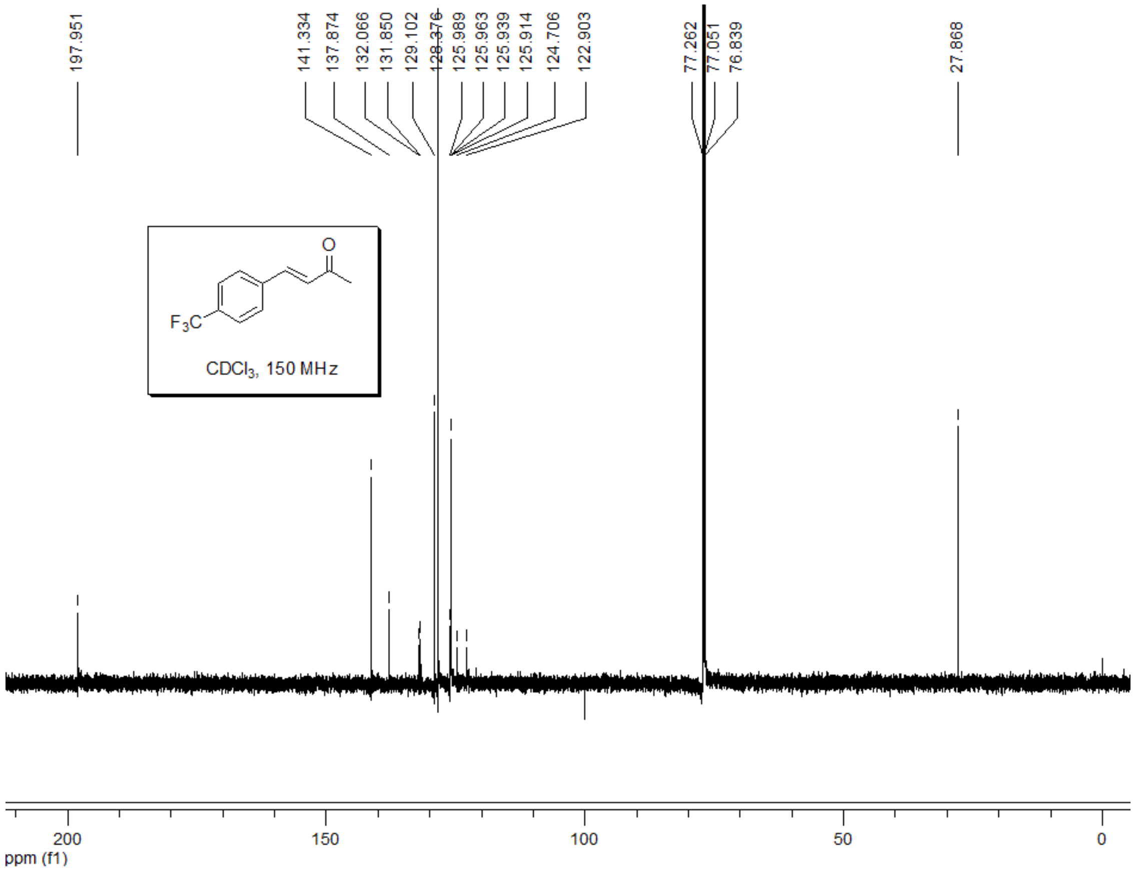
197.951
141.334
137.874
132.066
131.850
129.102
128.376
125.989
125.963
125.939
125.914
124.706
122.903
77.262
77.051
76.839
27.868
O
F3C
CDCl3, 150 MHz
200
150
100
50
0
ppm (f1)

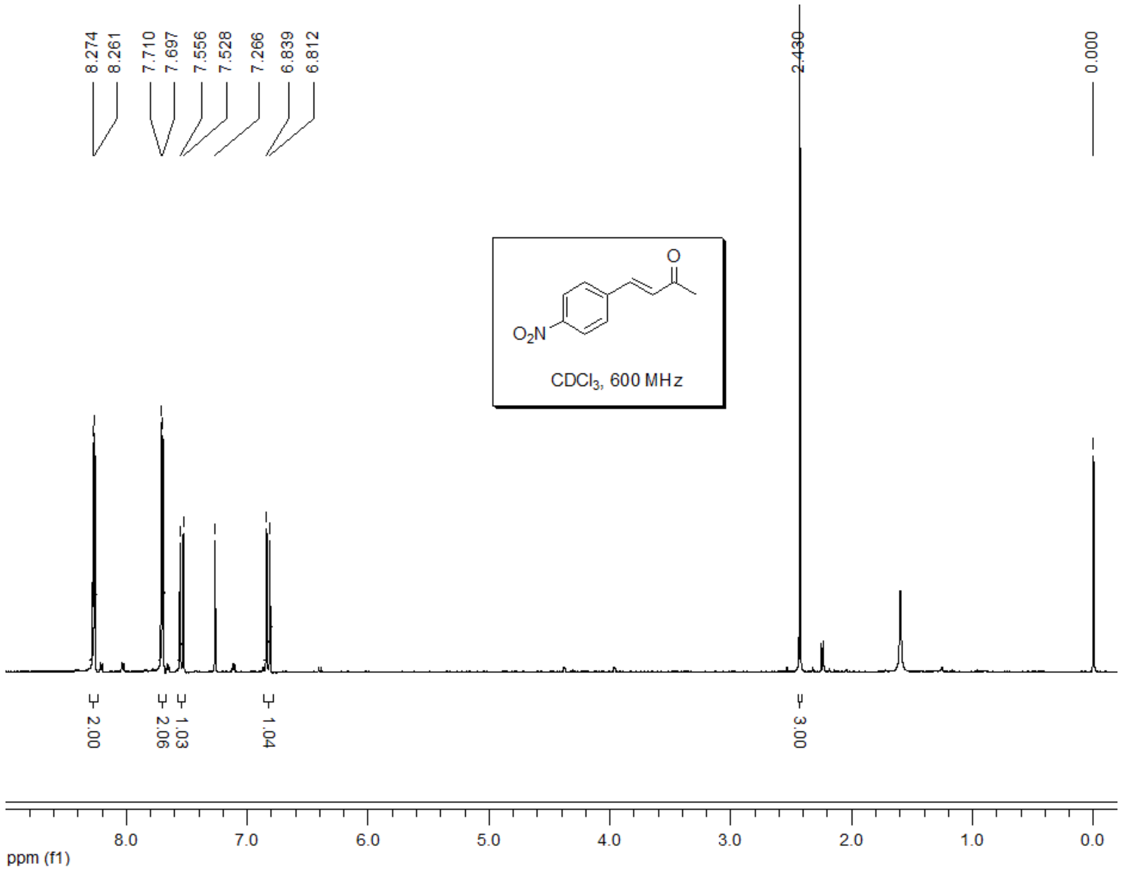
8.274
8.261
7.710
7.697
7.556
7.528
7.266
6.839
6.812
2.430
0.000
O
O2N
CDCl3, 600 MHz
2.00
2.06
1.03
1.04
3.00
8.0
7.0
6.0
5.0
4.0
3.0
2.0
1.0
0.0
ppm (f1)

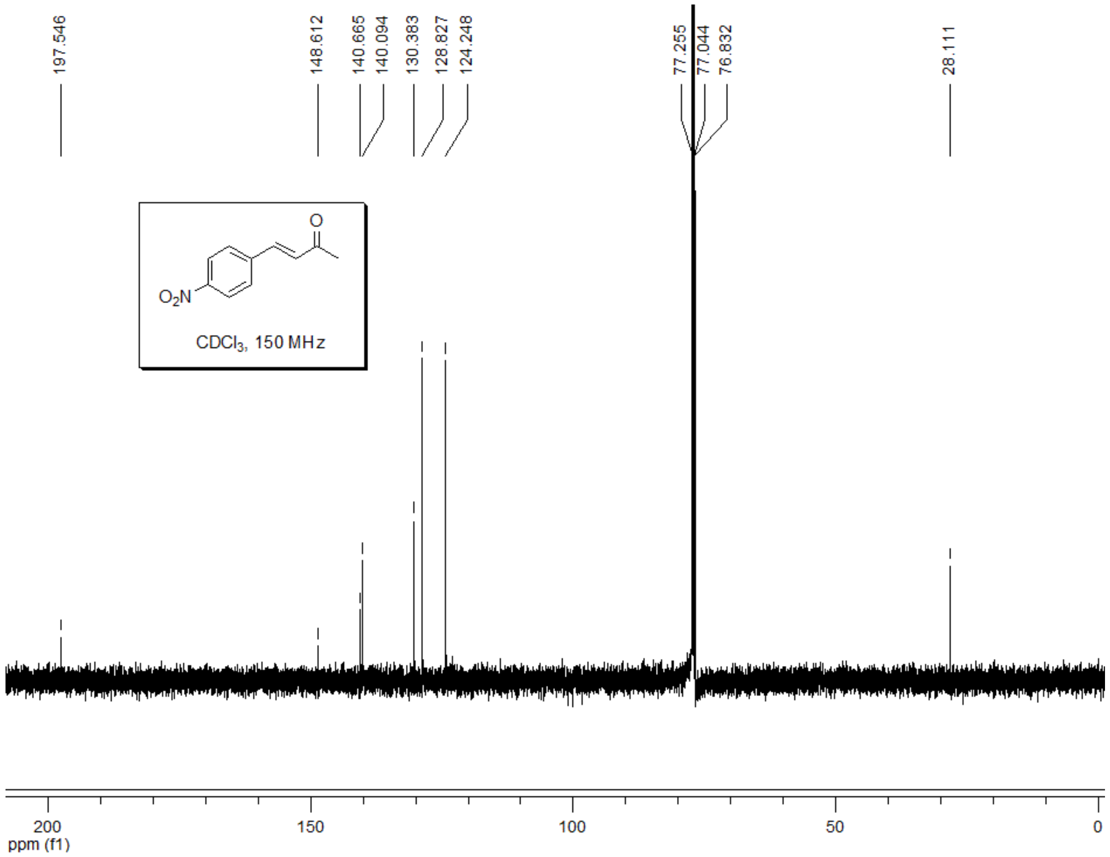
197.546
148.612
140.665
140.094
130.383
128.827
124.248
77.255
77.044
76.832
28.111
O
O2N
CDCl3, 150 MHz
200
150
100
50
0
ppm (f1)

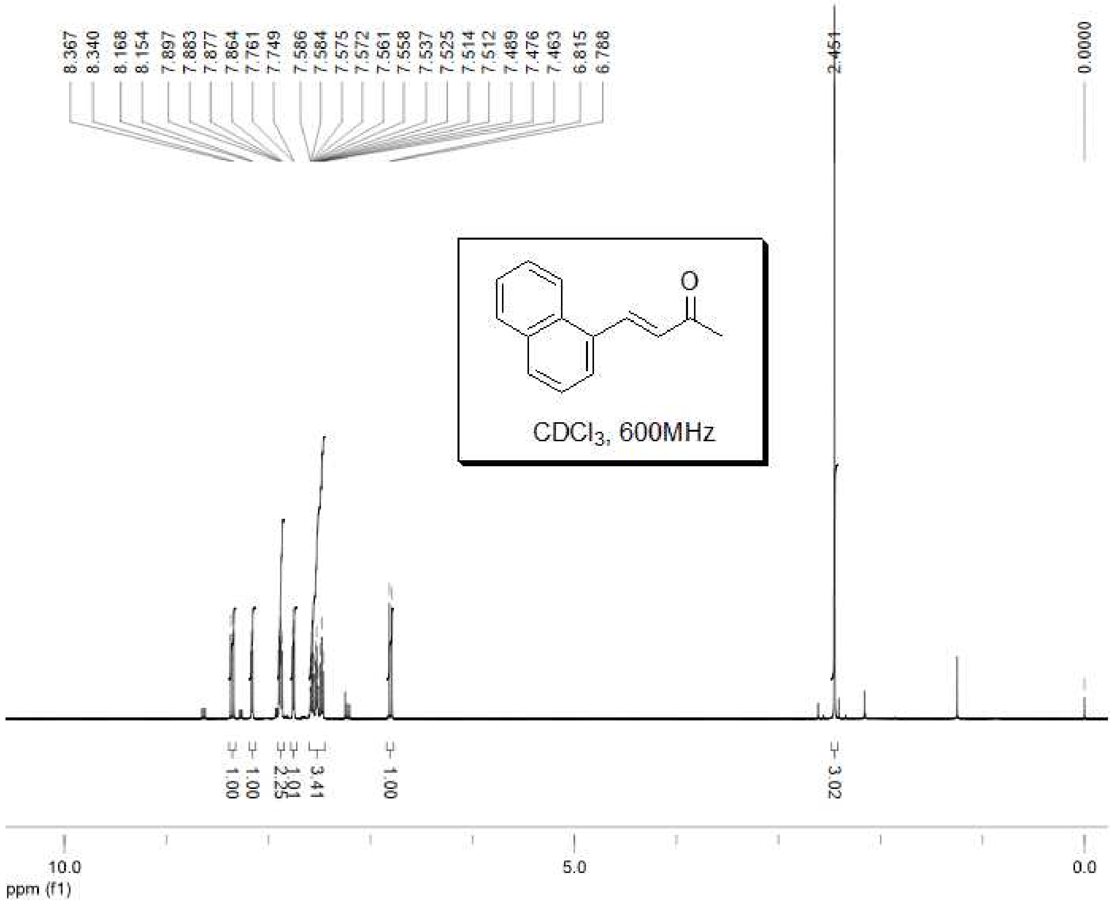
8.367
8.340
8.168
8.154
7.897
7.883
7.877
7.864
7.761
7.749
7.586
7.584
7.575
7.572
7.561
7.558
7.537
7.525
7.514
7.512
7.489
7.476
7.463
6.815
6.788
2.451
0.0000
O
CDCl3, 600MHz
1.00
1.00
2.25
1.01
3.41
1.00
3.02
10.0
5.0
0.0
ppm (f1)

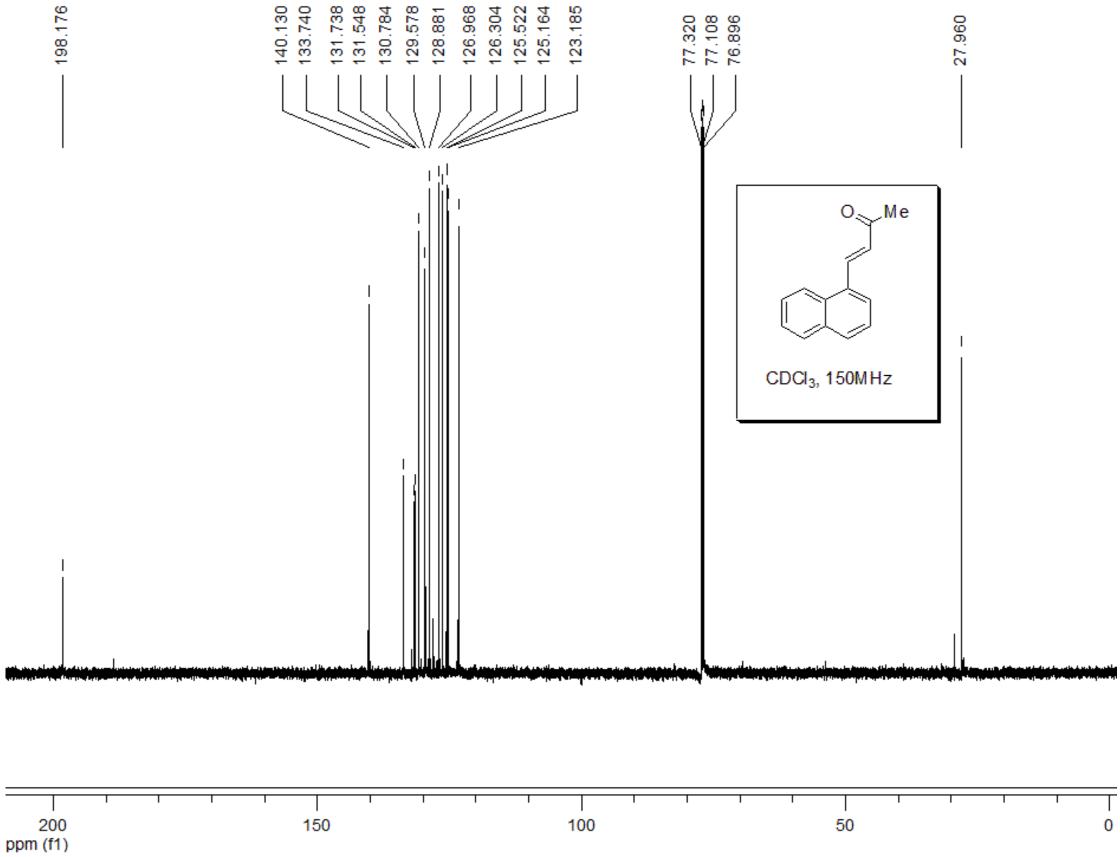
198.176
140.130
133.740
131.738
131.548
130.784
129.578
128.881
126.968
126.304
125.522
125.164
123.185
77.320
77.108
76.896
27.960
O
Me
CDCl3, 150MHz
200
150
100
50
0
ppm (f1)

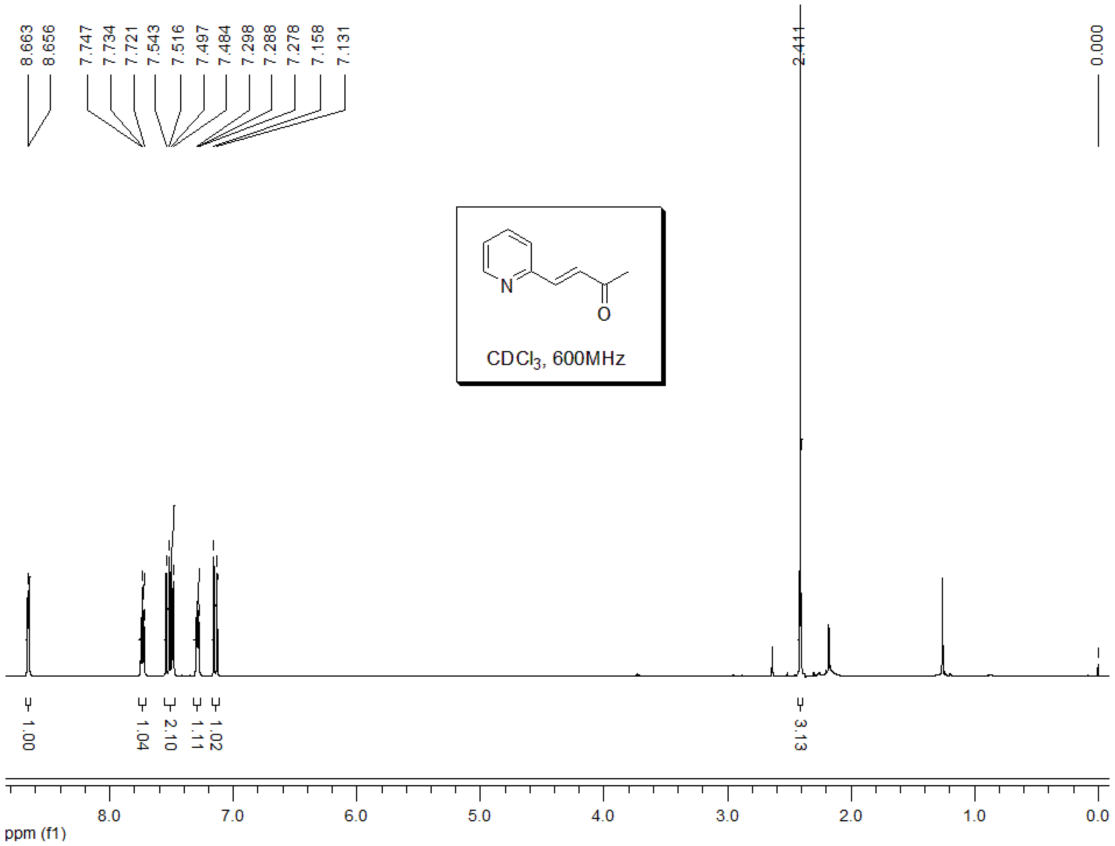
8.663
8.656
7.747
7.734
7.721
7.543
7.516
7.497
7.484
7.298
7.288
7.278
7.158
7.131
2.411
0.000
N
O
CDCl3, 600MHz
1.00
1.04
2.10
1.11
1.02
3.13
8.0
7.0
6.0
5.0
4.0
3.0
2.0
1.0
0.0
ppm (f1)

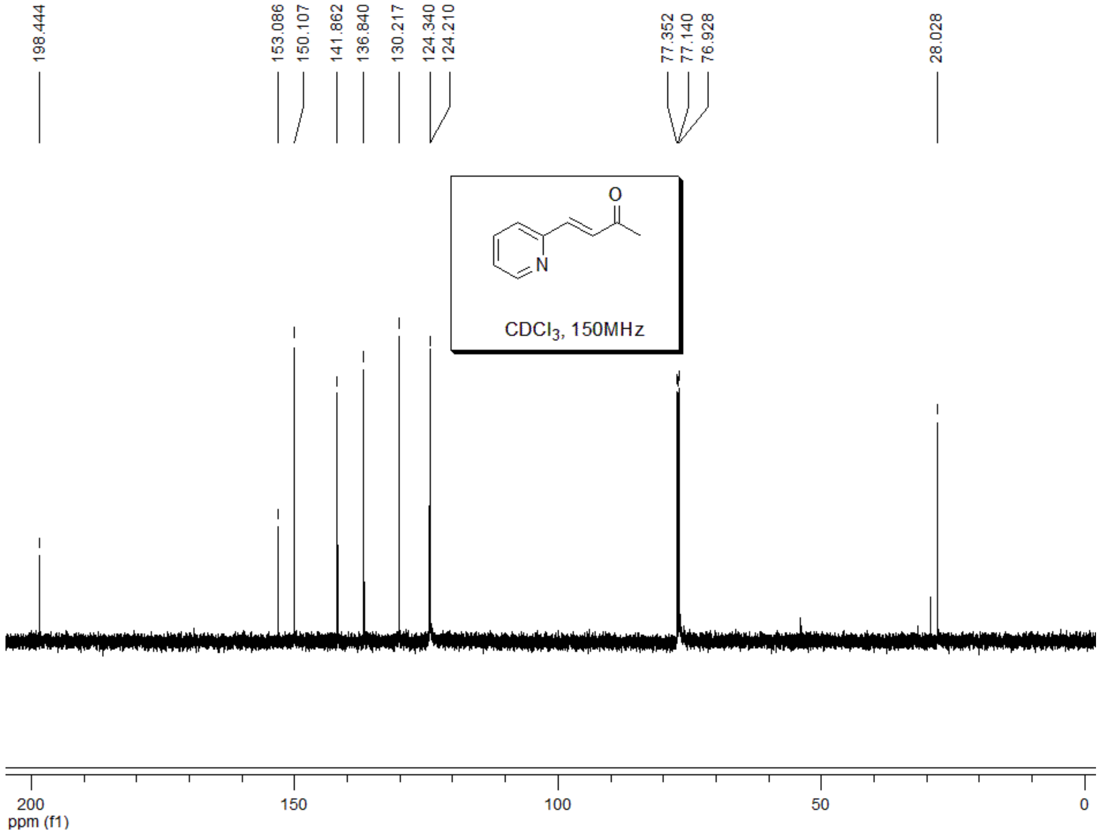
198.444
153.086
150.107
141.862
136.840
130.217
124.340
124.210
77.352
77.140
76.928
28.028
O
N
CDCl3, 150MHz
200
150
100
50
0
ppm (f1)

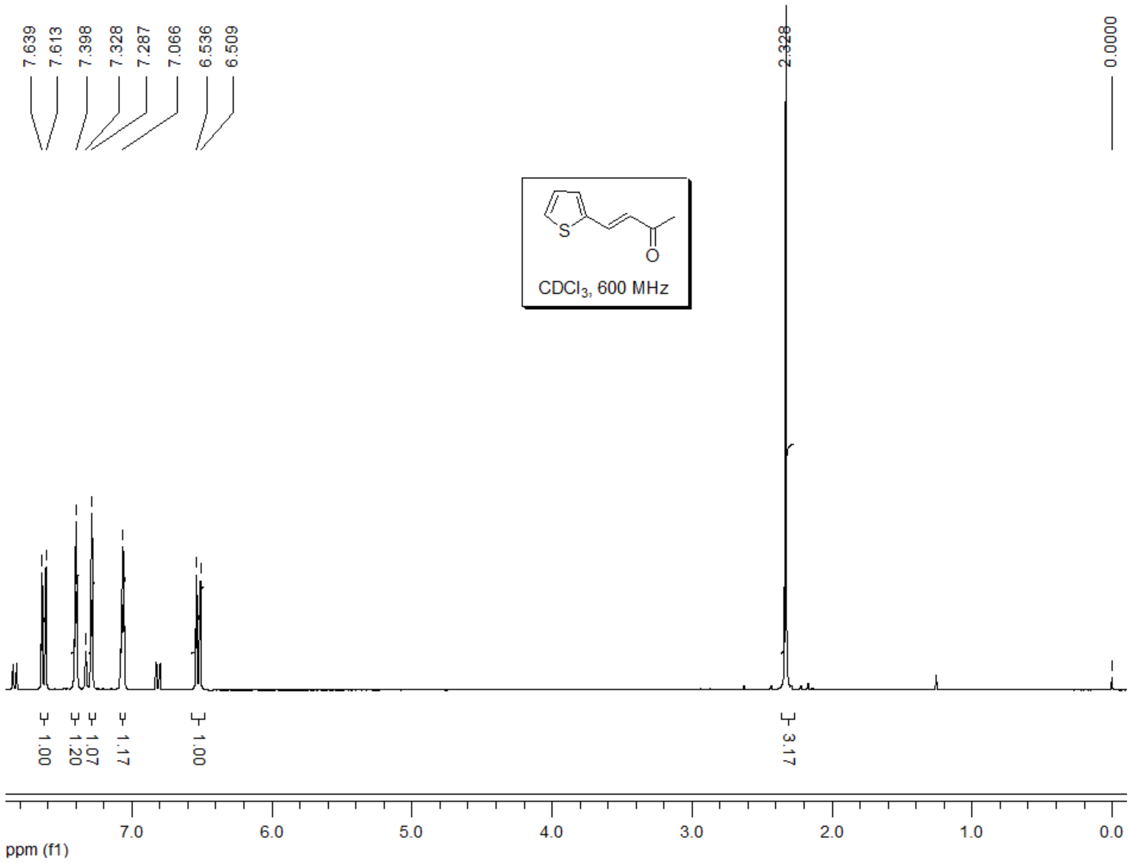
7.639
7.613
7.398
7.328
7.287
7.066
6.536
6.509
2.328
0.0000
S
O
CDCl3, 600 MHz
1.00
1.20
1.07
1.17
1.00
3.17
7.0
6.0
5.0
4.0
3.0
2.0
1.0
0.0
ppm (f1)

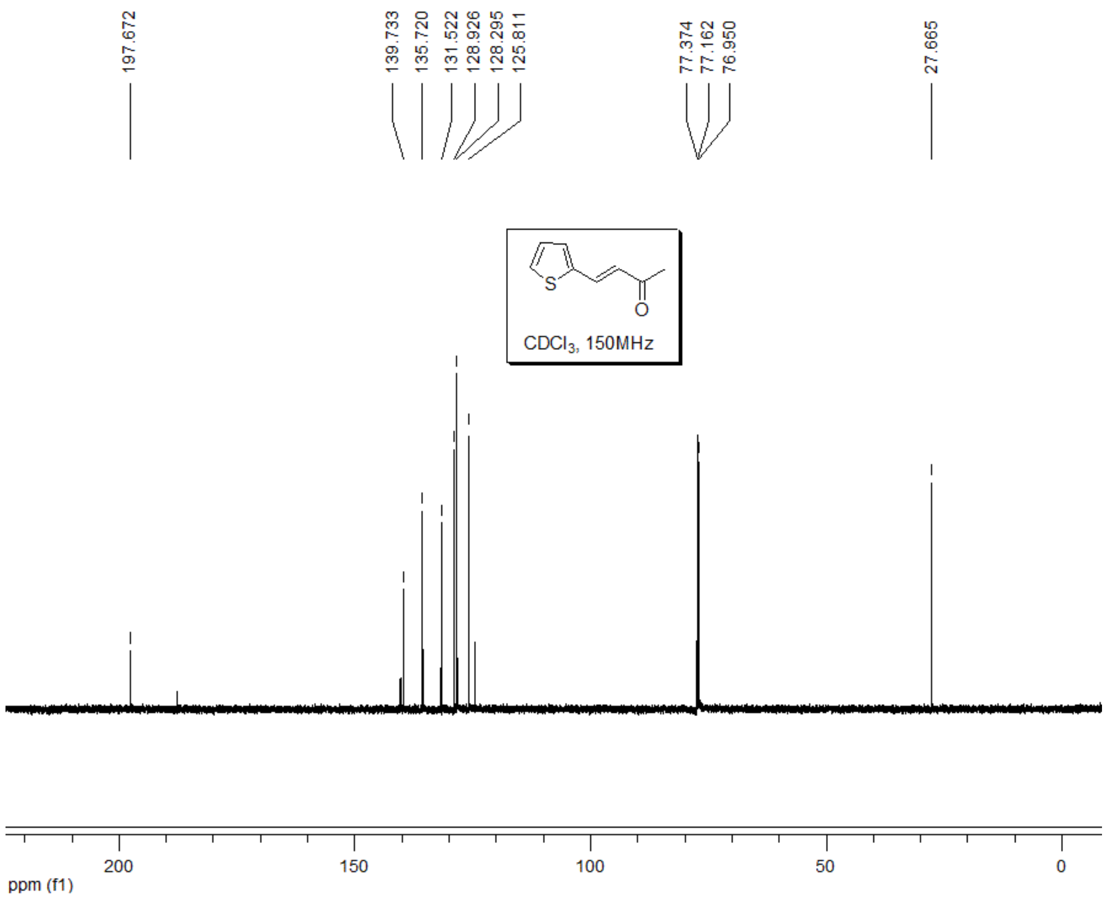
197.672
139.733
135.720
131.522
128.926
128.295
125.811
77.374
77.162
76.950
27.665
S
O
CDCl3, 150MHz
200
150
100
50
0
ppm (f1)

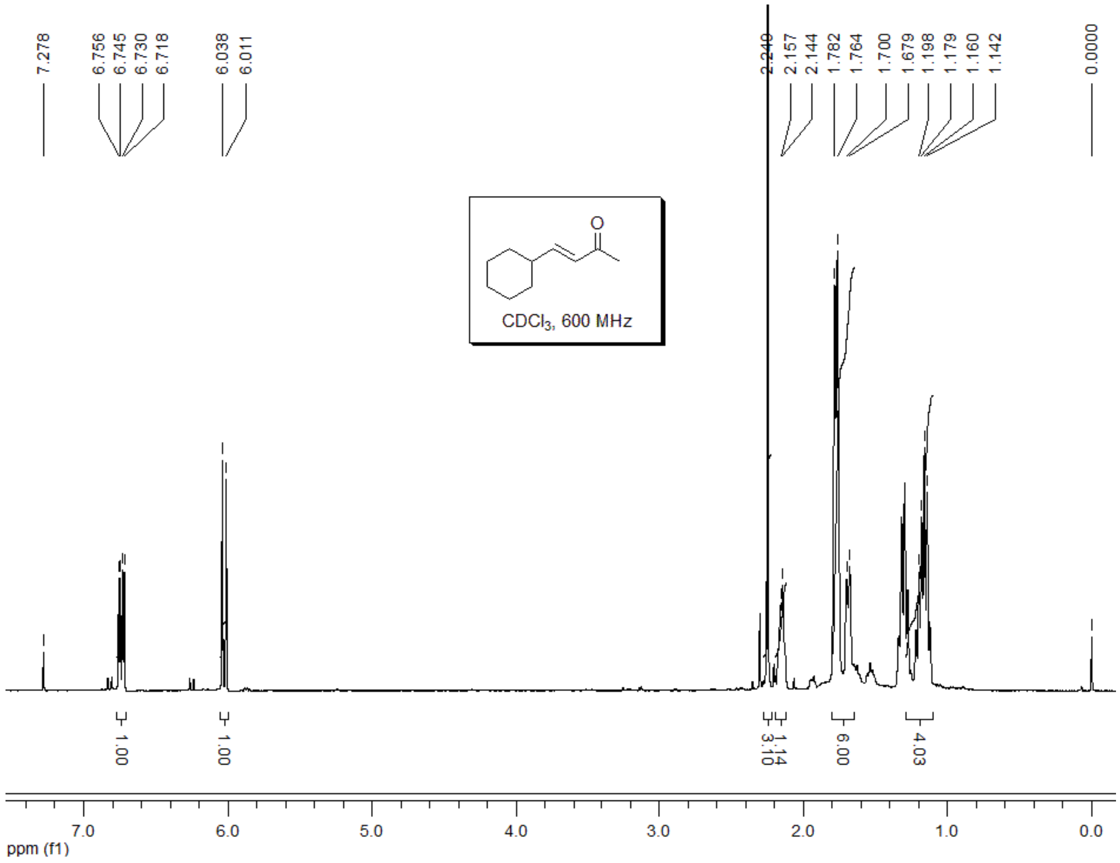
7.278
6.756
6.745
6.730
6.718
6.038
6.011
2.240
2.157
2.144
1.782
1.764
1.700
1.679
1.198
1.179
1.160
1.142
0.0000
O
CDCl3, 600 MHz
1.00
1.00
3.10
1.14
6.00
4.03
7.0
6.0
5.0
4.0
3.0
2.0
1.0
0.0
ppm (f1)

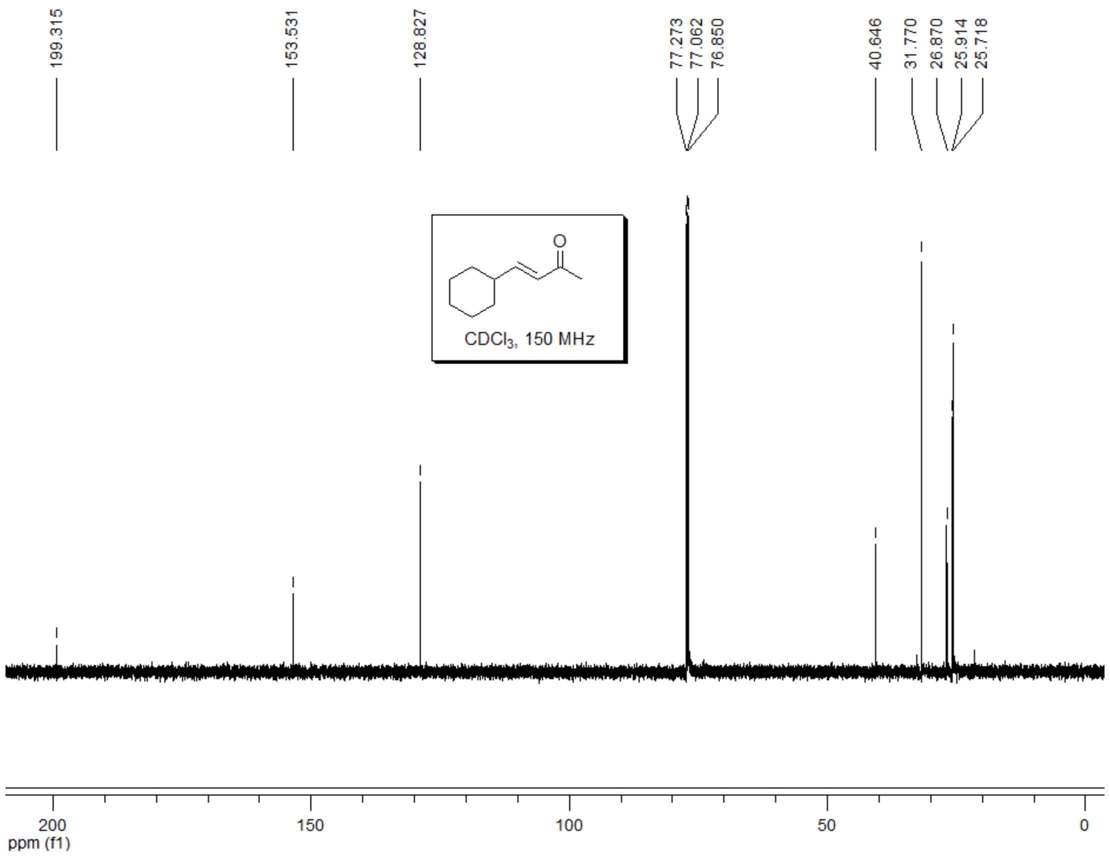
199.315
153.531
128.827
77.273
77.062
76.850
40.646
31.770
26.870
25.914
25.718
O
CDCl3, 150 MHz
200
150
100
50
0
ppm (f1)

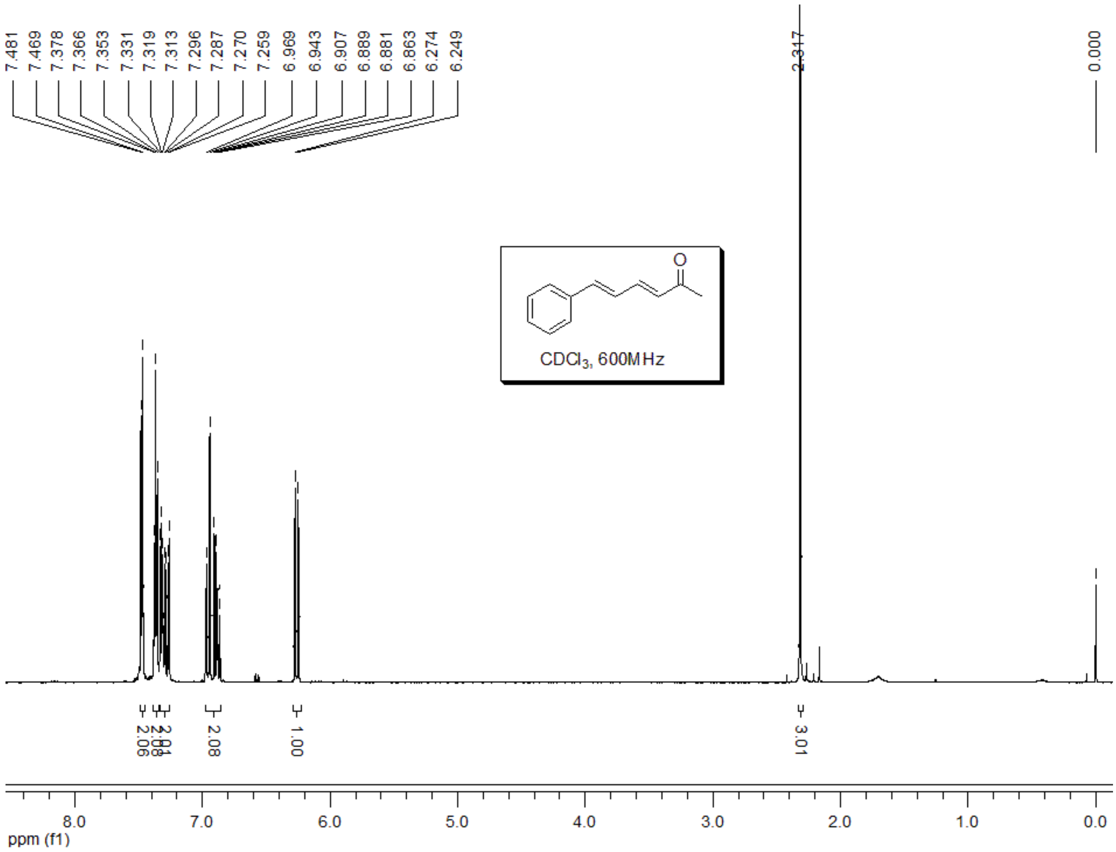
7.481
7.469
7.378
7.366
7.353
7.331
7.319
7.313
7.296
7.287
7.270
7.259
6.969
6.943
6.907
6.889
6.881
6.863
6.274
6.249
2.317
0.000
O
CDCl3, 600MHz
2.06
2.08
1.00
3.01
8.0
7.0
6.0
5.0
4.0
3.0
2.0
1.0
0.0
ppm (f1)

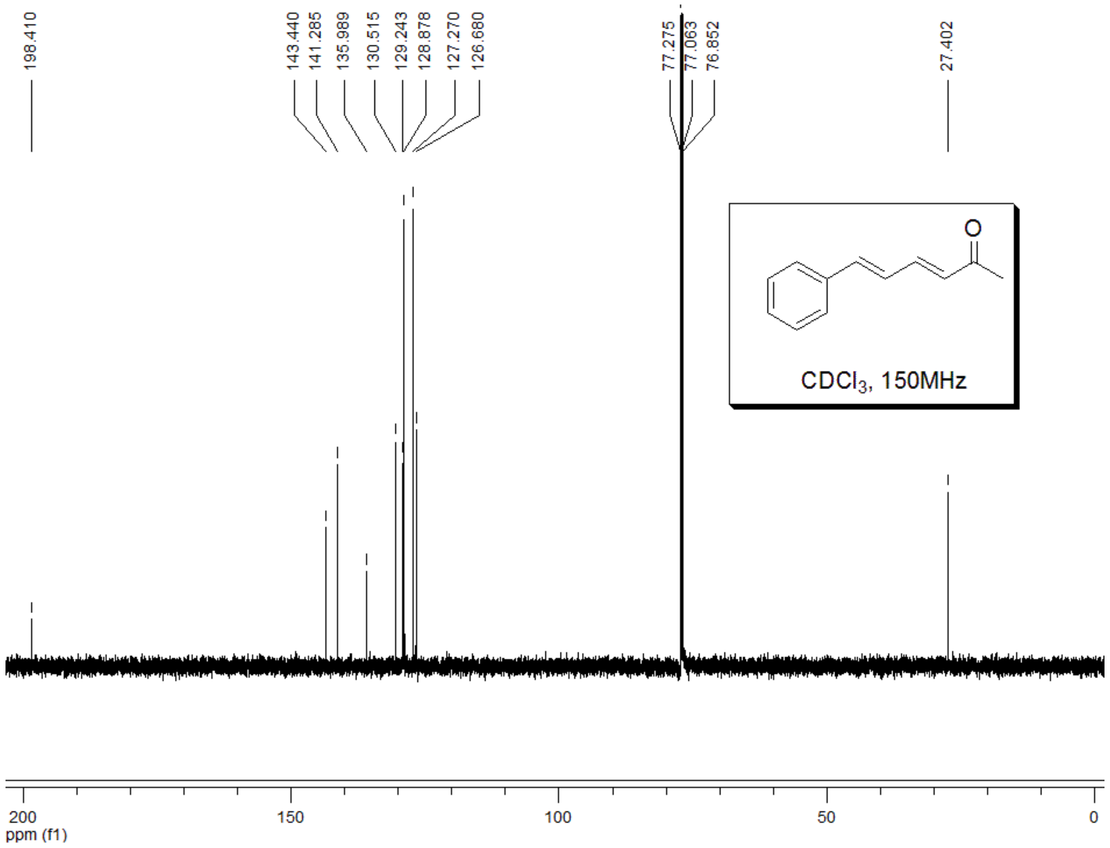
198.410
143.440
141.285
135.989
130.515
129.243
128.878
127.270
126.680
77.275
77.063
76.852
27.402
CDCl3, 150MHz
200
150
100
50
0
ppm (f1)

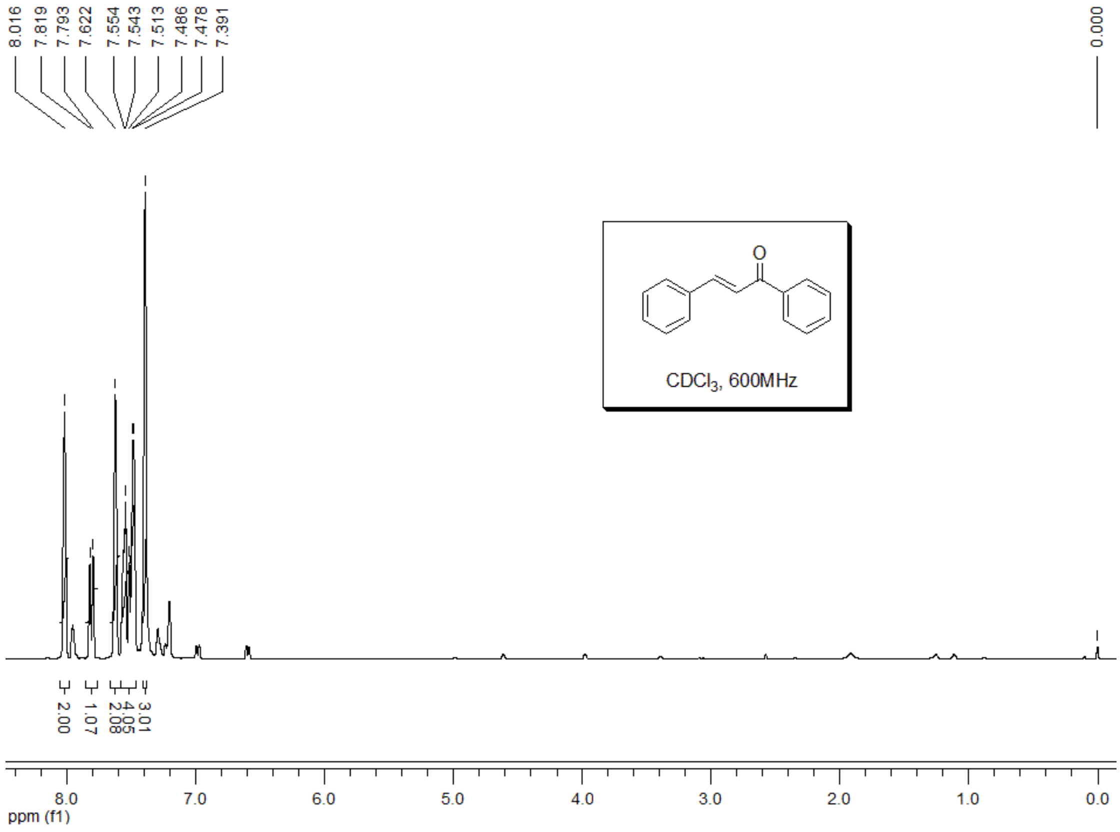
8.016
7.819
7.793
7.622
7.554
7.543
7.513
7.486
7.478
7.391
0.000
CDCl3, 600MHz
2.00
1.07
2.08
4.05
3.01
8.0
7.0
6.0
5.0
4.0
3.0
2.0
1.0
0.0
ppm (f1)

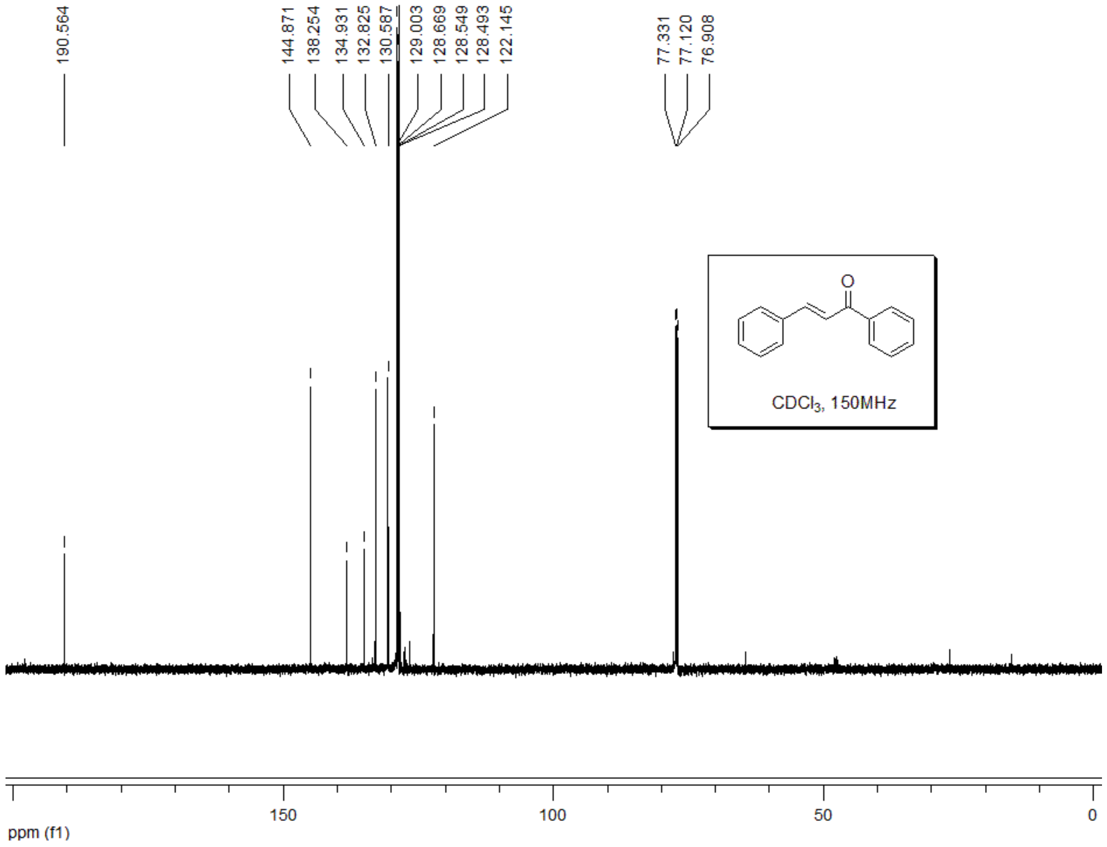
190.564
144.871
138.254
134.931
132.825
130.587
129.003
128.669
128.549
128.493
122.145
77.331
77.120
76.908
O
CDCl3, 150MHz
150
100
50
0
ppm (f1)

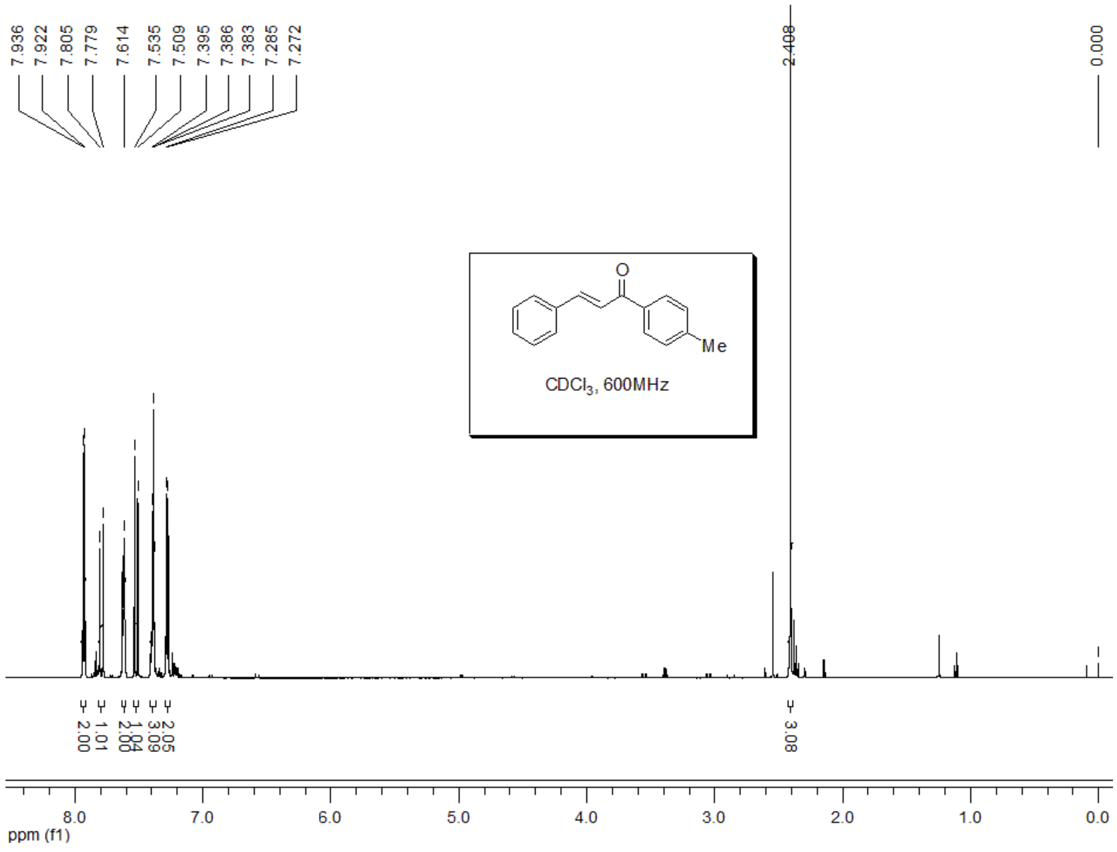
7.936
7.922
7.805
7.779
7.614
7.535
7.509
7.395
7.386
7.383
7.285
7.272
2.408
0.000
O
Me
CDCl3, 600MHz
2.00
1.01
1.04
2.00
3.09
2.05
3.08
8.0
7.0
6.0
5.0
4.0
3.0
2.0
1.0
0.0
ppm (f1)

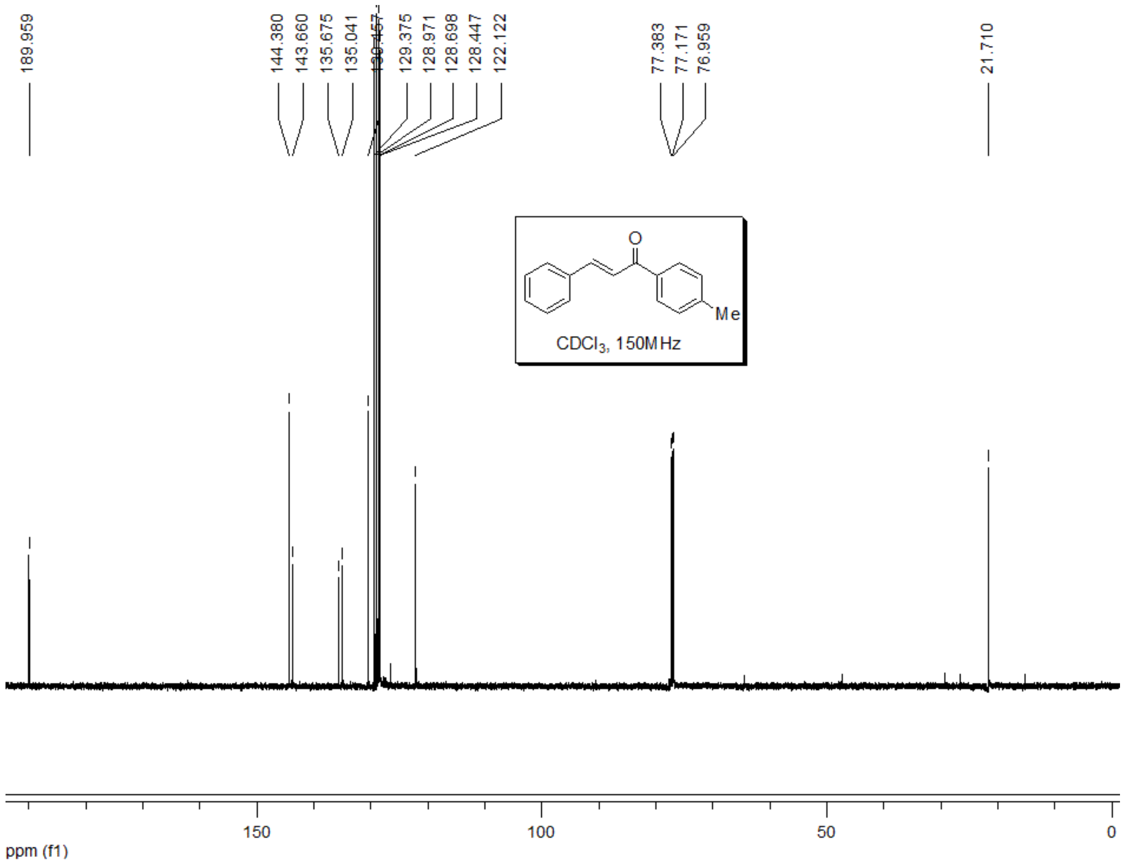
189.959
144.380
143.660
135.675
135.041
129.375
128.971
128.698
128.447
122.122
77.383
77.171
76.959
21.710
O
Me
CDCl3, 150MHz
150
100
50
0
ppm (f1)

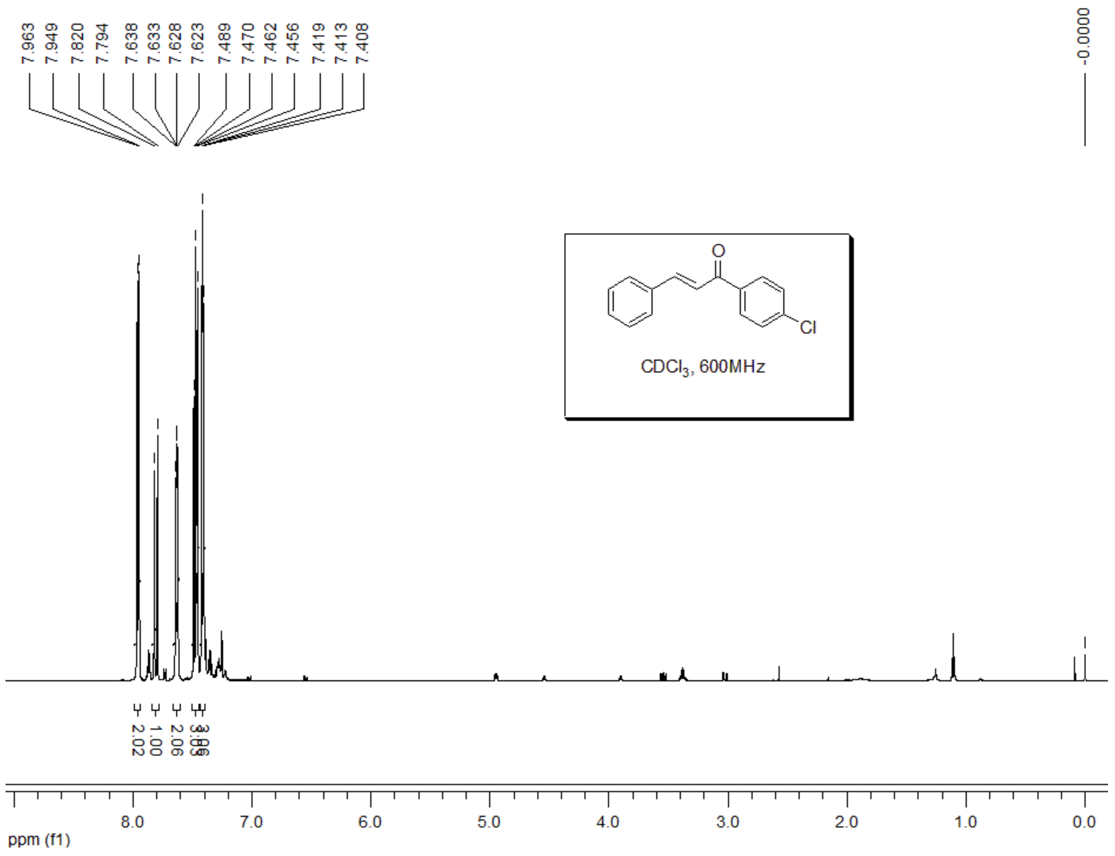
7.963
7.949
7.820
7.794
7.638
7.633
7.628
7.623
7.489
7.470
7.462
7.456
7.419
7.413
7.408
-0.0000
O
Cl
CDCl3, 600MHz
2.02
1.00
2.06
8.0
7.0
6.0
5.0
4.0
3.0
2.0
1.0
0.0
ppm (f1)

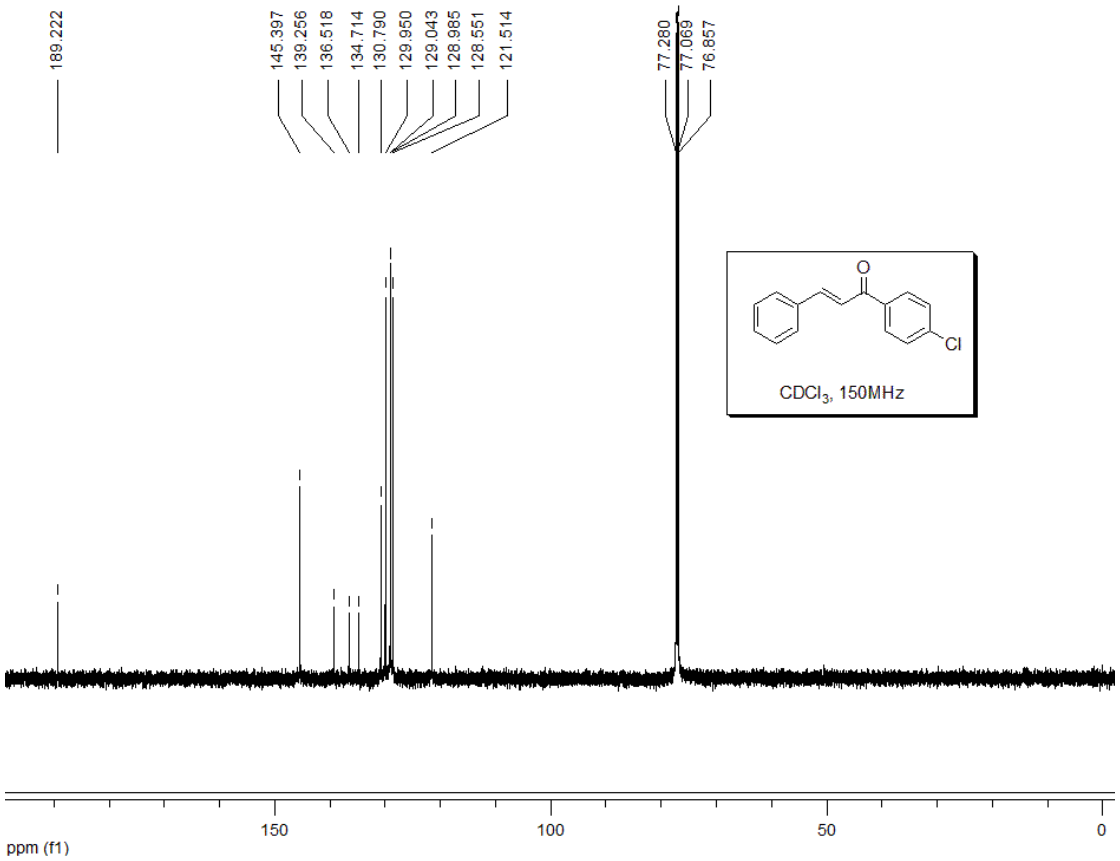
189.222
145.397
139.256
136.518
134.714
130.790
129.950
129.043
128.985
128.551
121.514
77.280
77.069
76.857
O
Cl
CDCl3, 150MHz
150
100
50
0
ppm (f1)

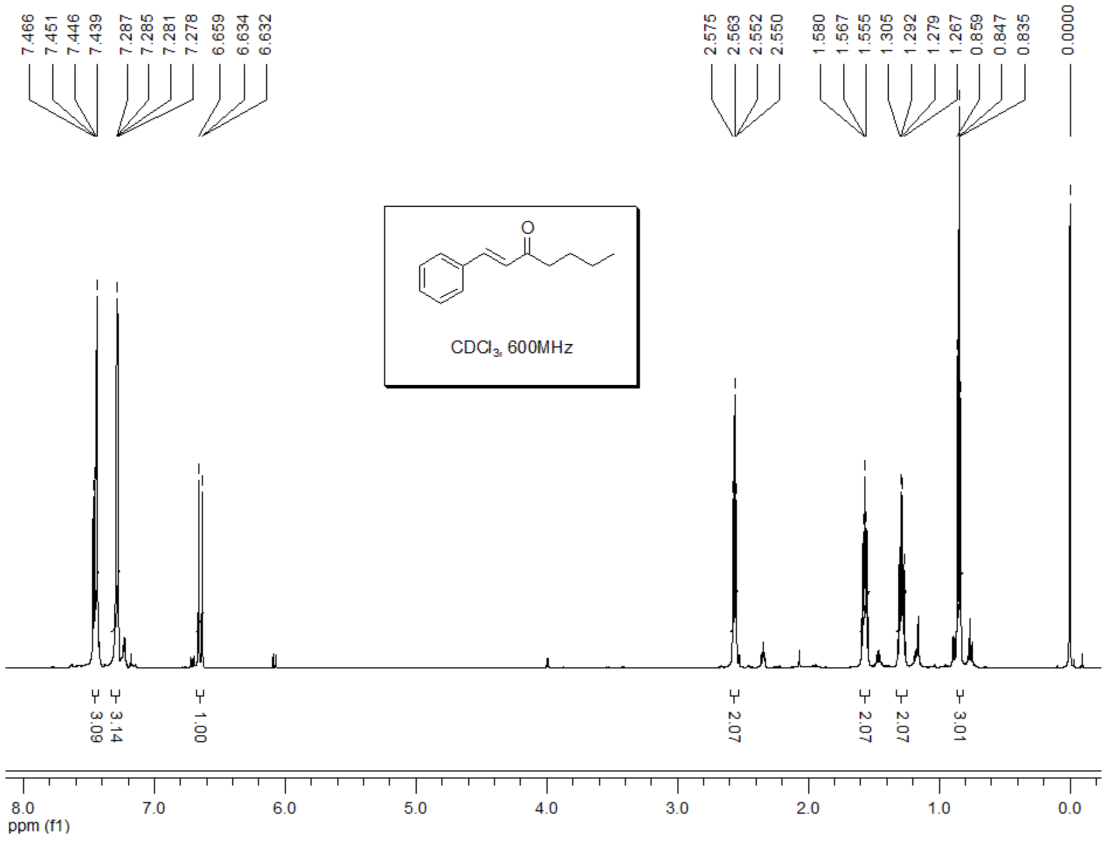
7.466
7.451
7.446
7.439
7.287
7.285
7.281
7.278
6.659
6.634
6.632
2.575
2.563
2.552
2.550
1.580
1.567
1.555
1.305
1.292
1.279
1.267
0.859
0.847
0.835
0.0000
O
CDCl3, 600MHz
3.09
3.14
1.00
2.07
2.07
2.07
3.01
8.0
7.0
6.0
5.0
4.0
3.0
2.0
1.0
0.0
ppm (f1)

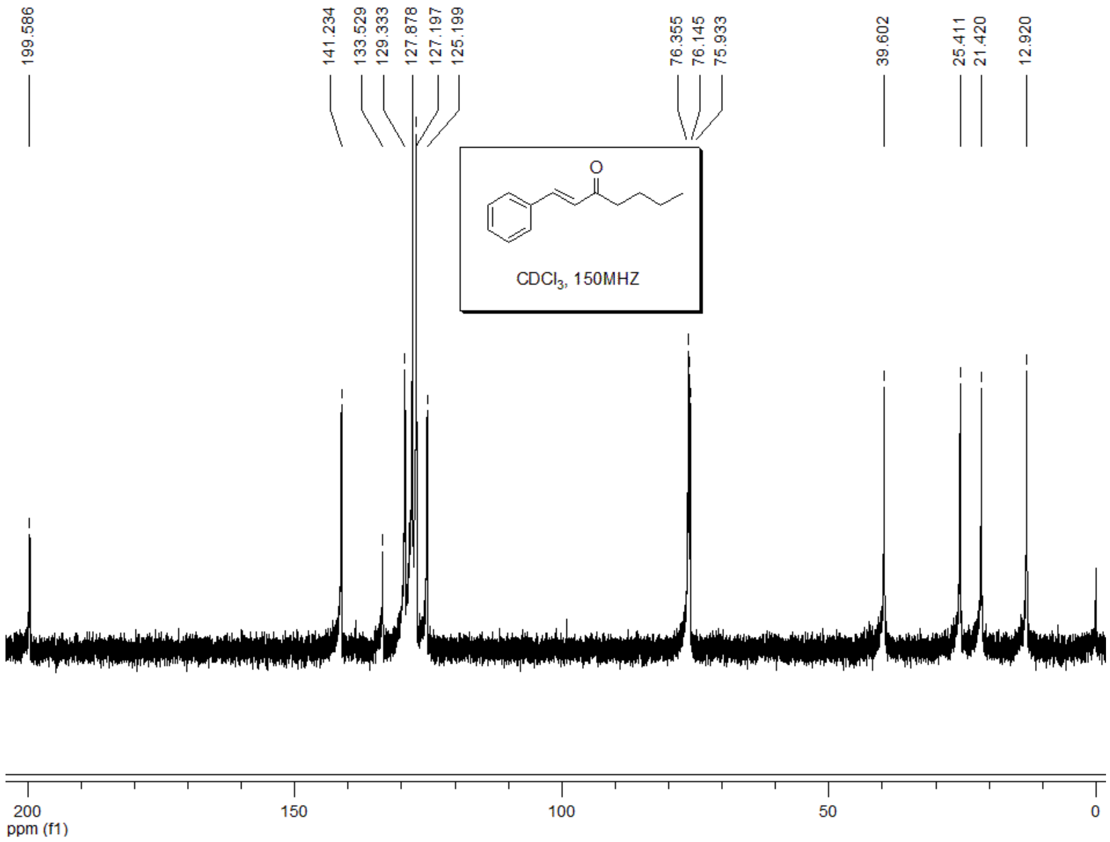
199.586
141.234
133.529
129.333
127.878
127.197
125.199
76.355
76.145
75.933
39.602
25.411
21.420
12.920
O
CDCl3, 150MHZ
200
150
100
50
0
ppm (f1)

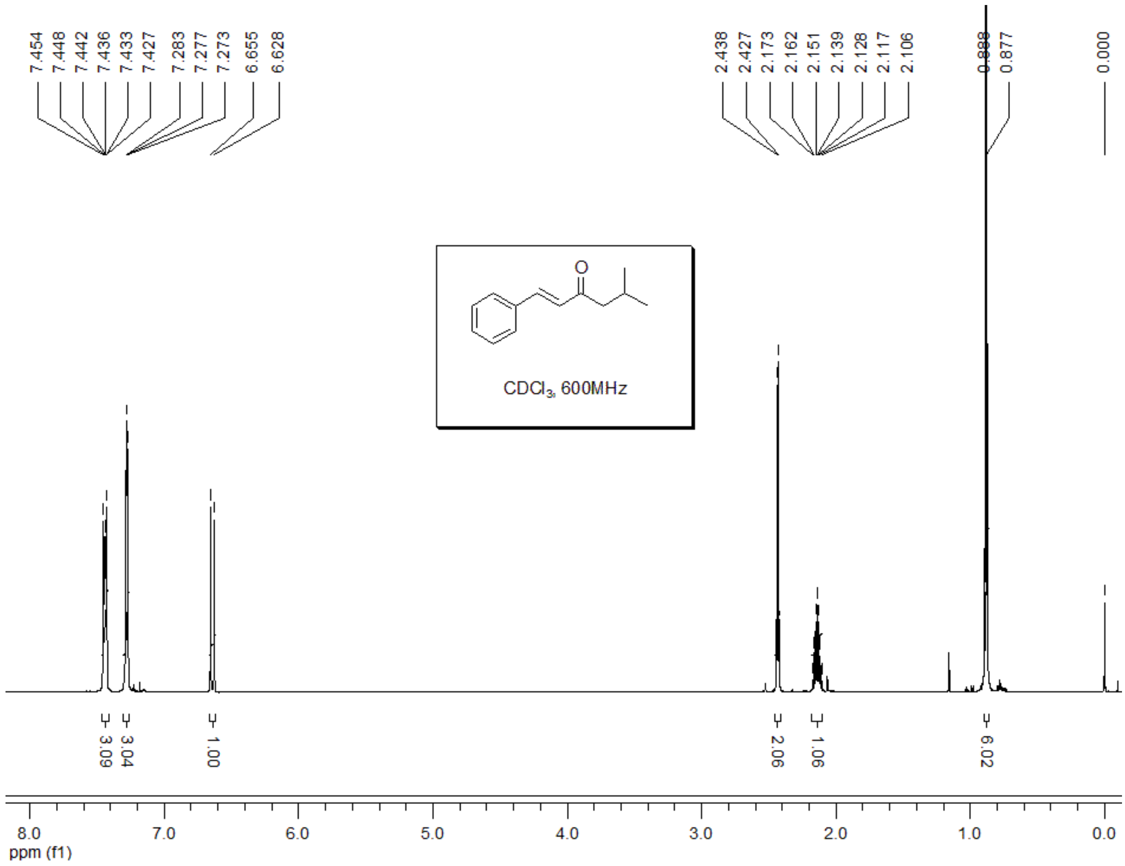
7.454
7.448
7.442
7.436
7.433
7.427
7.283
7.277
7.273
6.655
6.628
2.438
2.427
2.173
2.162
2.151
2.139
2.128
2.117
2.106
0.888
0.877
0.000
O
CDCl3, 600MHz
3.09
3.04
1.00
2.06
1.06
6.02
8.0
7.0
6.0
5.0
4.0
3.0
2.0
1.0
0.0
ppm (f1)

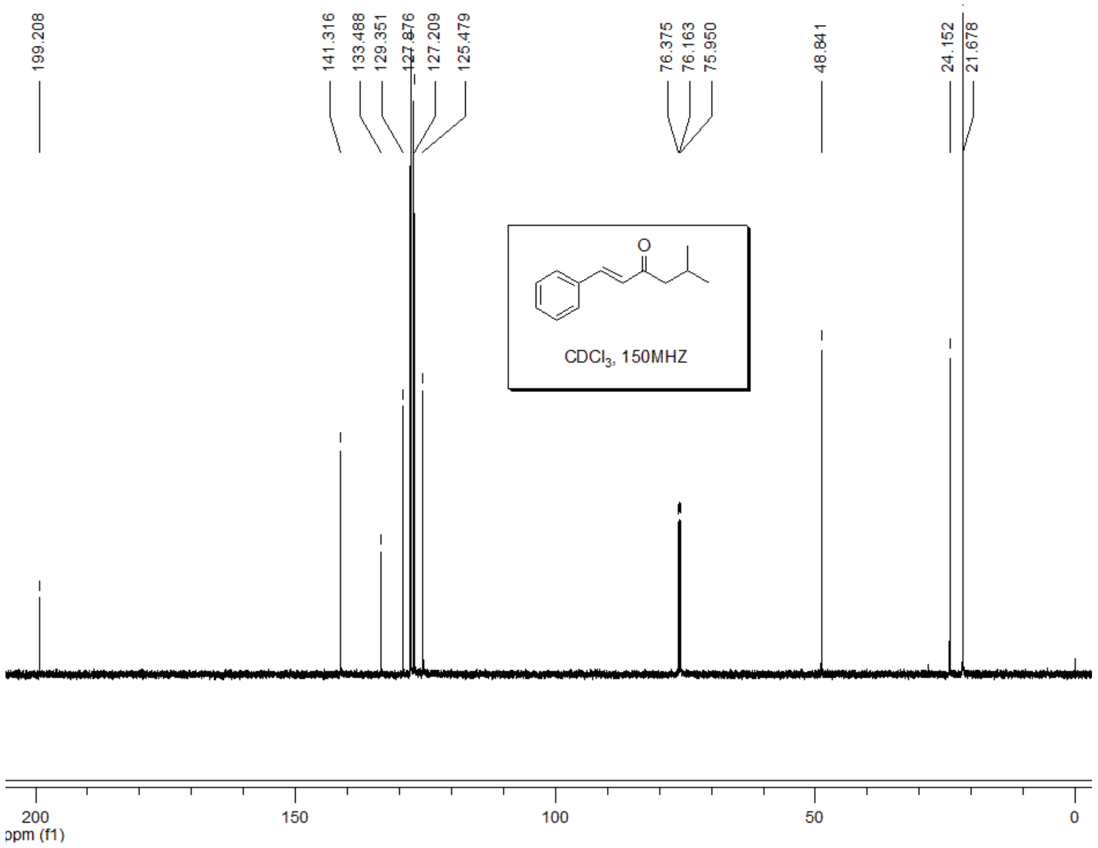
199.208
141.316
133.488
129.351
127.876
127.209
125.479
76.375
76.163
75.950
48.841
24.152
21.678
O
CDCl3, 150MHZ
200
150
100
50
0
ppm (f1)

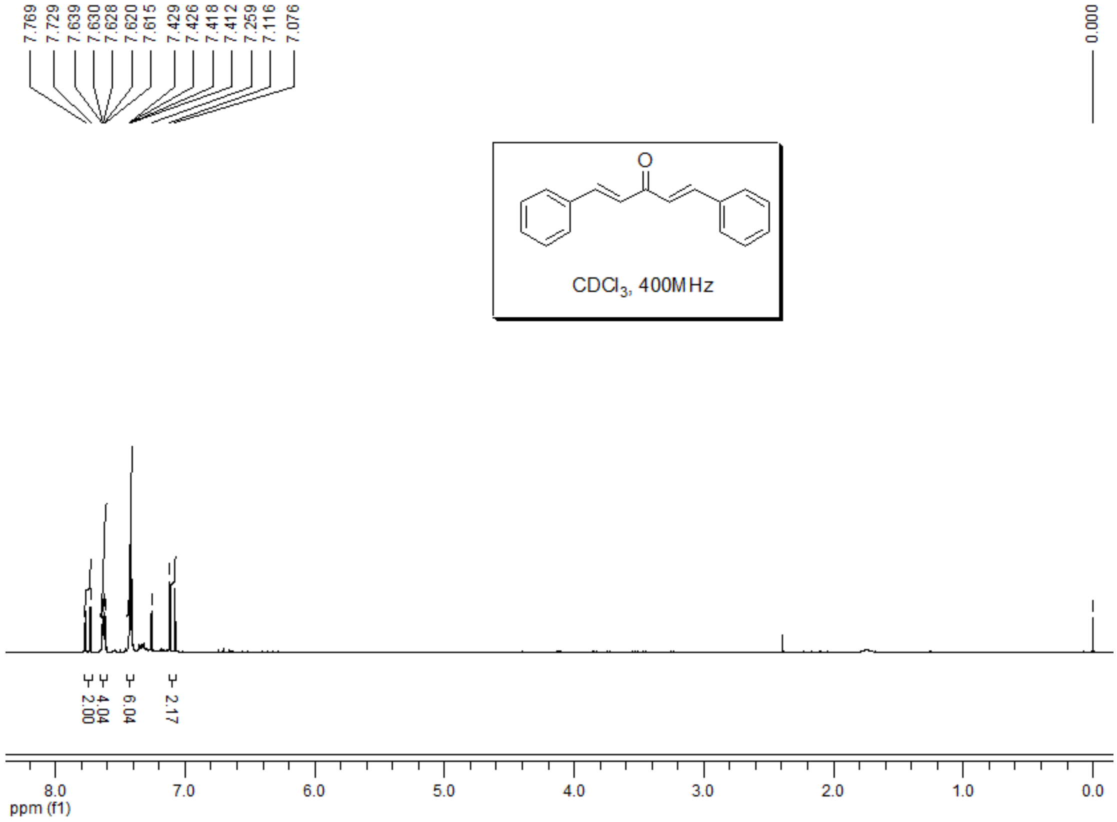
7.769
7.729
7.639
7.630
7.628
7.620
7.615
7.429
7.426
7.418
7.412
7.259
7.116
7.076
0.000
O
CDCl3, 400MHz
2.00
4.04
6.04
2.17
8.0
7.0
6.0
5.0
4.0
3.0
2.0
1.0
0.0
ppm (f1)

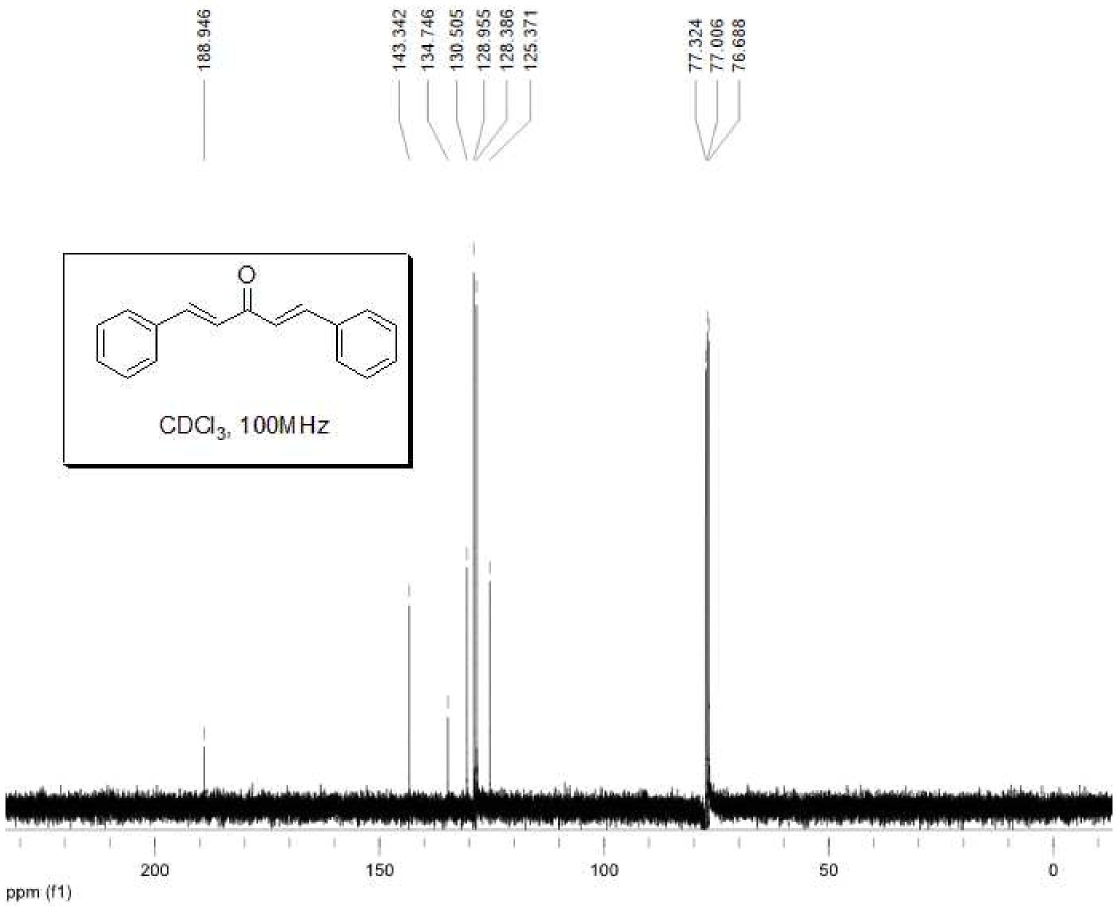
188.946
143.342
134.746
130.505
128.955
128.386
125.371
77.324
77.006
76.688
CDCl3, 100MHz
200
150
100
50
0
ppm (f1)

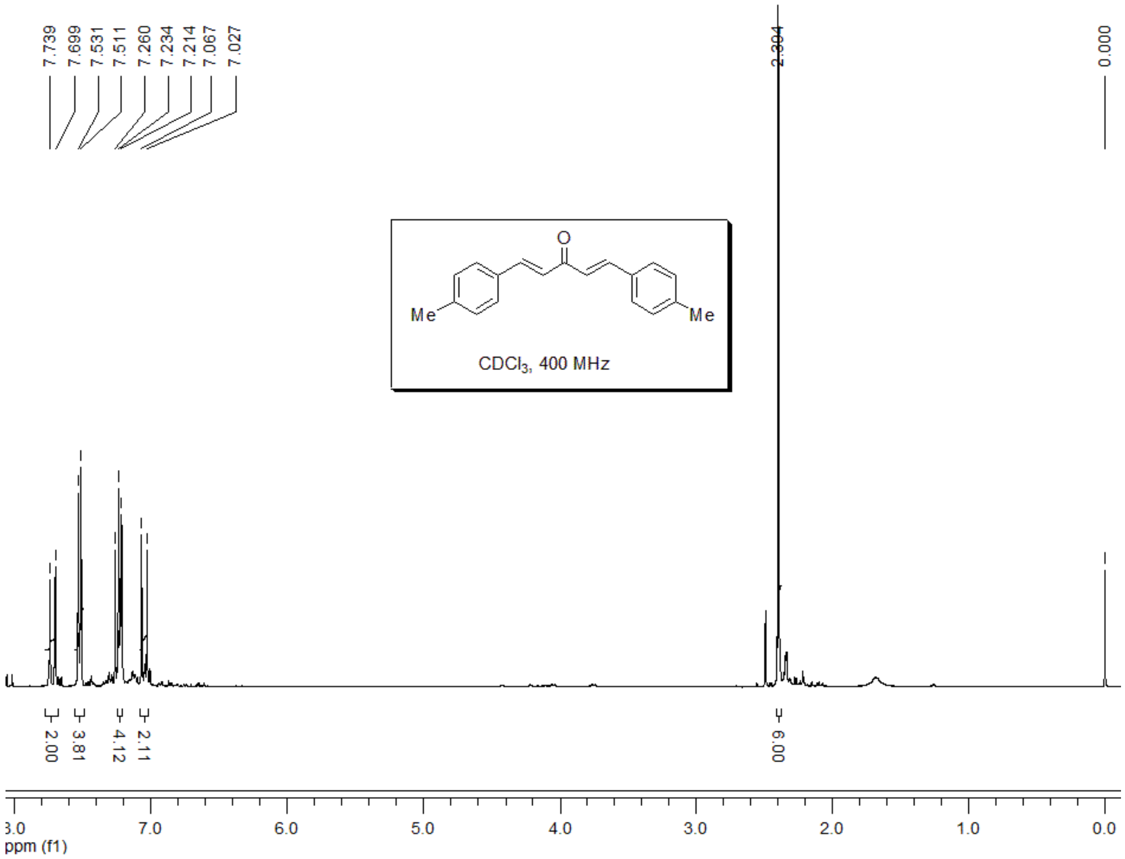
7.739
7.699
7.531
7.511
7.260
7.234
7.214
7.067
7.027
0.000
Me
Me
CDCl3, 400 MHz
2.00
3.81
4.12
2.11
6.00
7.0
6.0
5.0
4.0
3.0
2.0
1.0
0.0
ppm (f1)

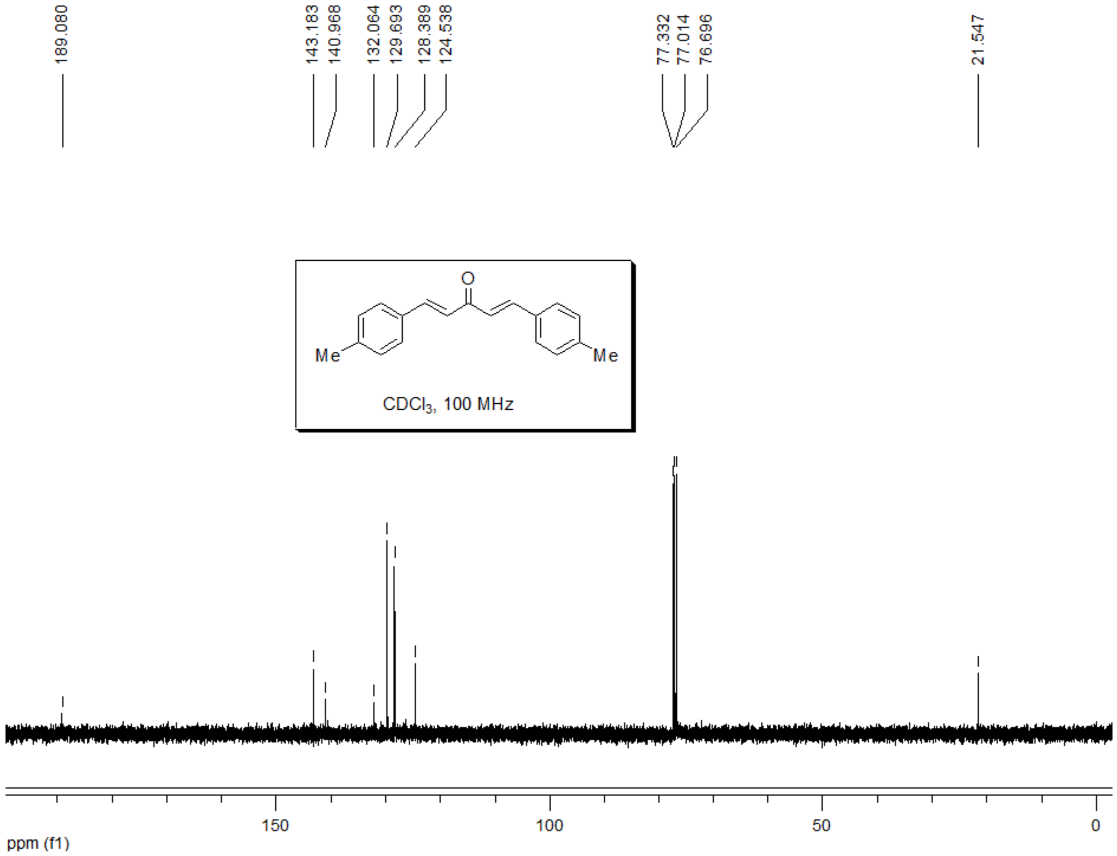

189.080
143.183
140.968
132.064
129.693
128.389
124.538
77.332
77.014
76.696
21.547
O
Me
Me
CDCl3, 100 MHz
150
100
50
0
ppm (f1)

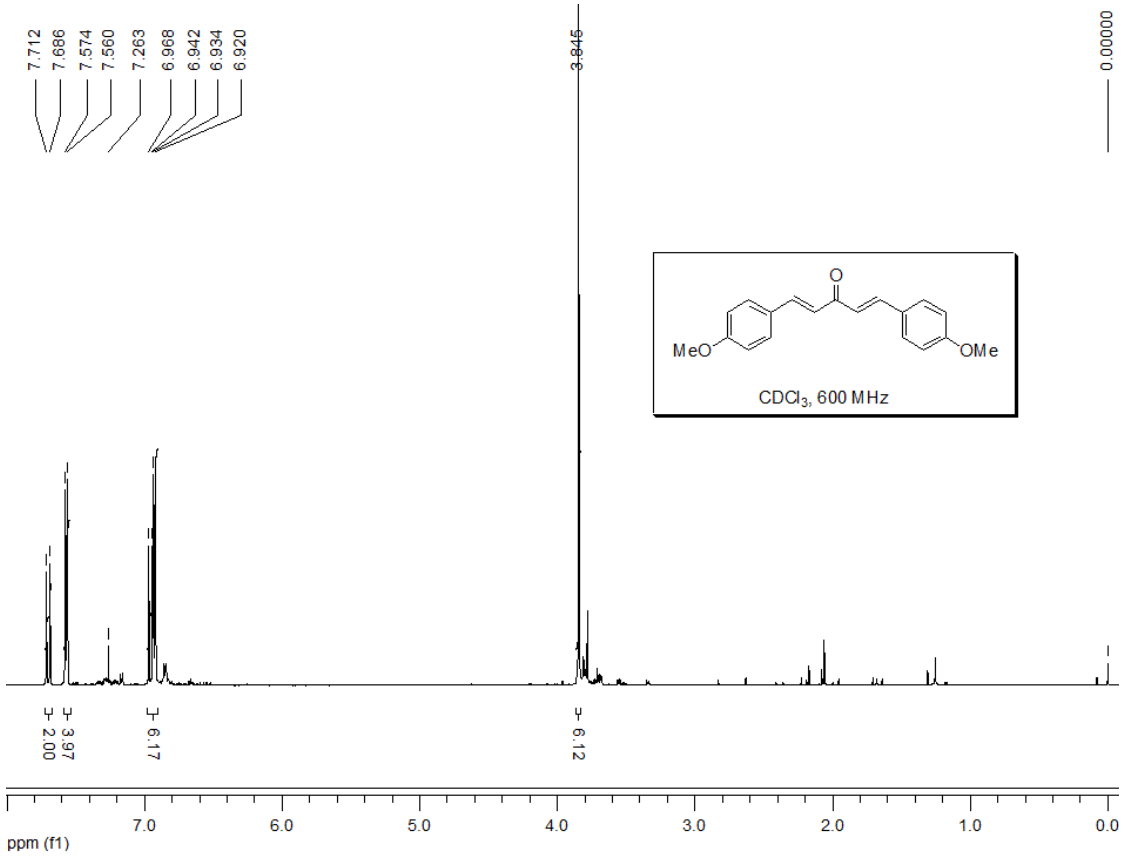

7.712
7.686
7.574
7.560
7.263
6.968
6.942
6.934
6.920
3.845
0.00000
O
MeO
OMe
CDCl3, 600 MHz
2.00
3.97
6.17
6.12
7.0
6.0
5.0
4.0
3.0
2.0
1.0
0.0
ppm (f1)

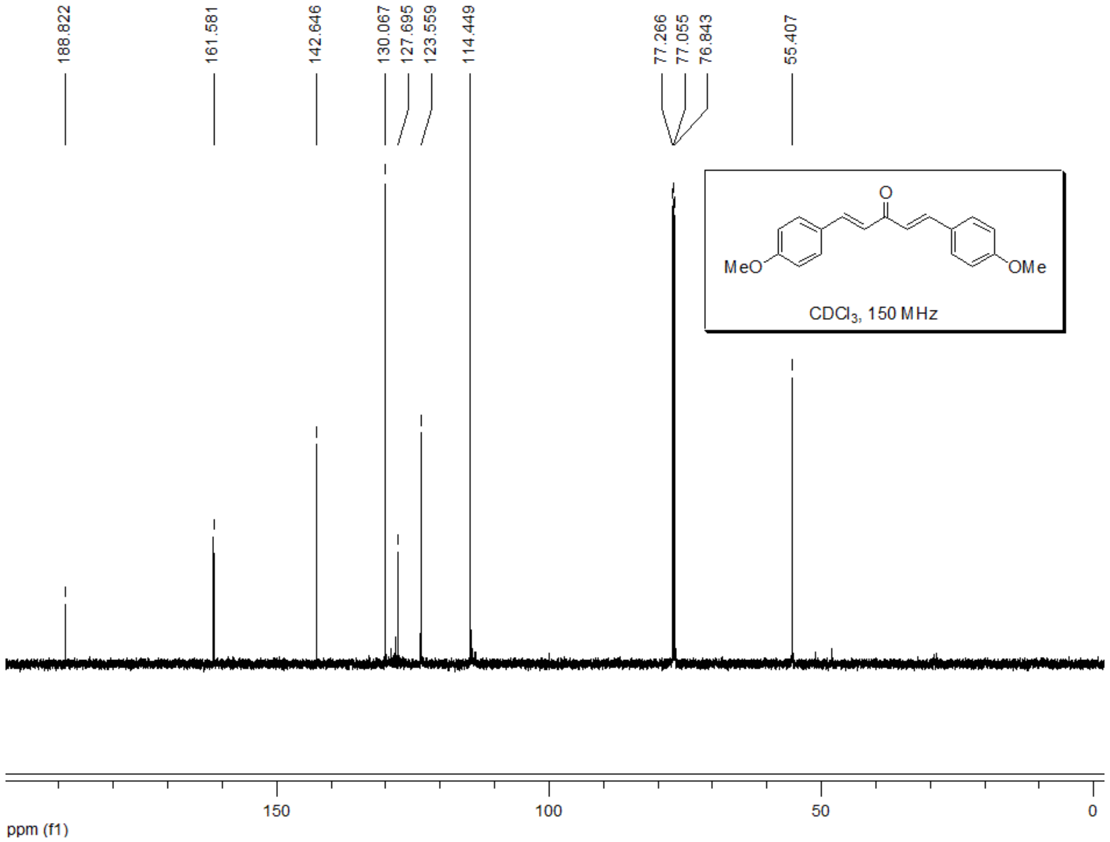
188.822
161.581
142.646
130.067
127.695
123.559
114.449
77.266
77.055
76.843
55.407
O
MeO
OMe
CDCl3, 150 MHz
150
100
50
0
ppm (f1)

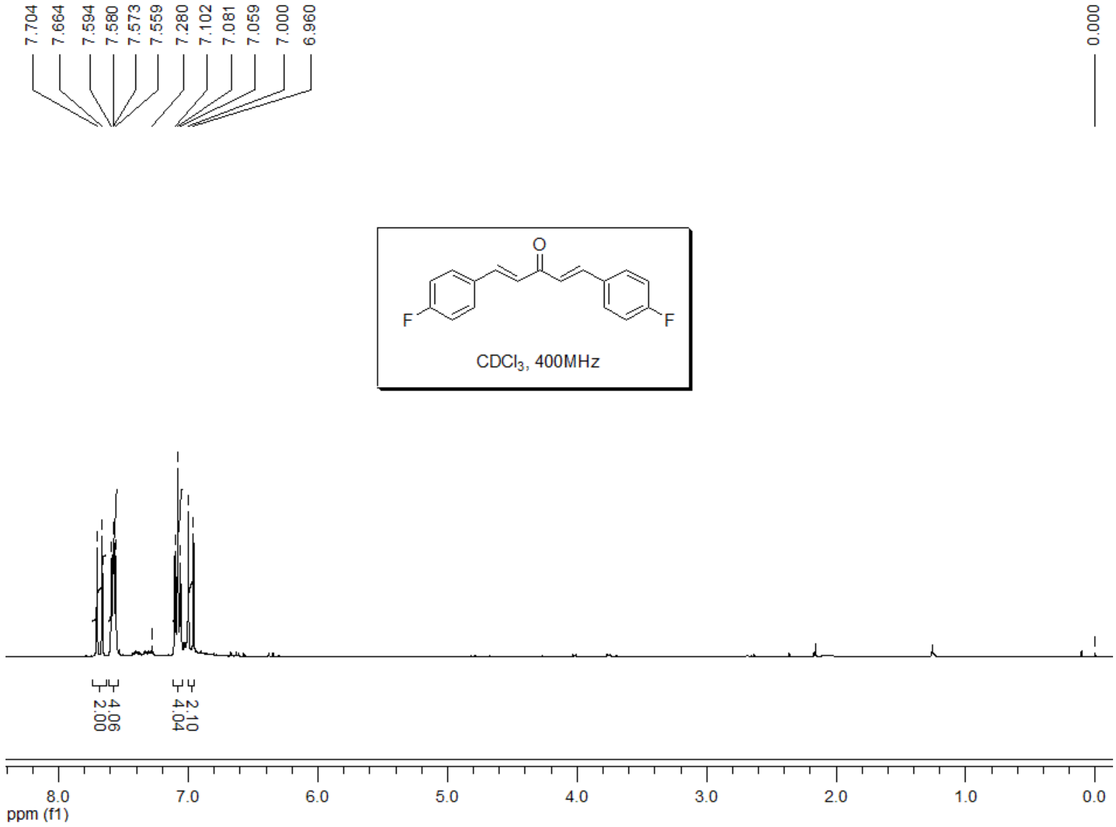
7.704
7.664
7.594
7.580
7.573
7.559
7.280
7.102
7.081
7.059
7.000
6.960
0.000
O
F
F
CDCl3, 400MHz
2.00
4.06
4.04
2.10
8.0
7.0
6.0
5.0
4.0
3.0
2.0
1.0
0.0
ppm (f1)

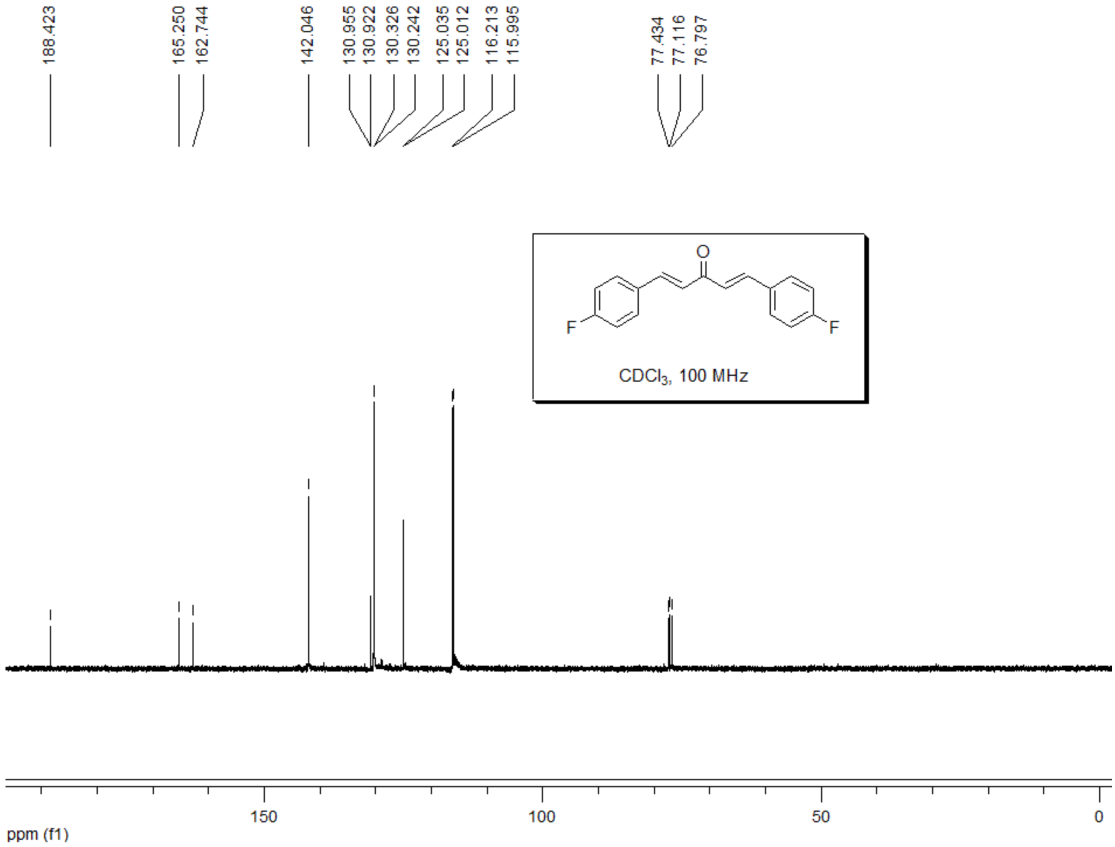
188.423
165.250
162.744
142.046
130.955
130.922
130.326
130.242
125.035
125.012
116.213
115.995
77.434
77.116
76.797
CDCl$_3$, 100 MHz
ppm (f1)
150
100
50
0

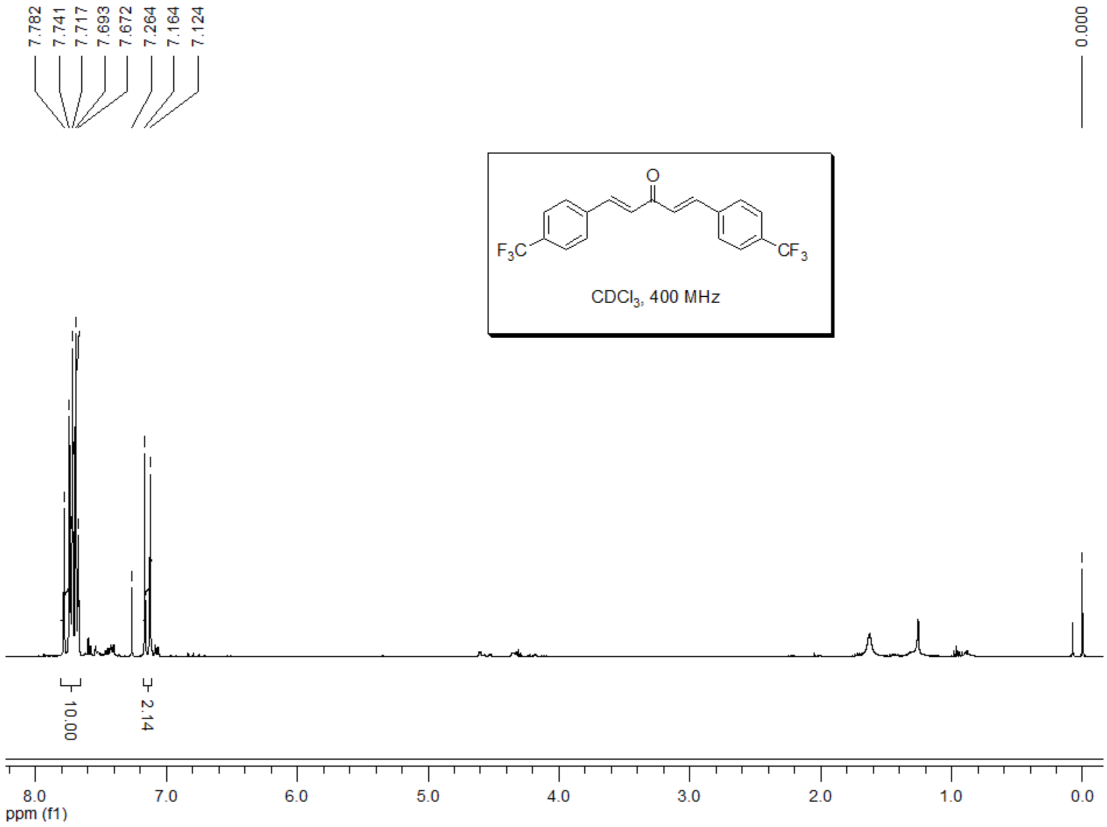
7.782
7.741
7.717
7.693
7.672
7.264
7.164
7.124
0.000
CDCl$_3$, 400 MHz
10.00
2.14
8.0
7.0
6.0
5.0
4.0
3.0
2.0
1.0
0.0
ppm (f1)

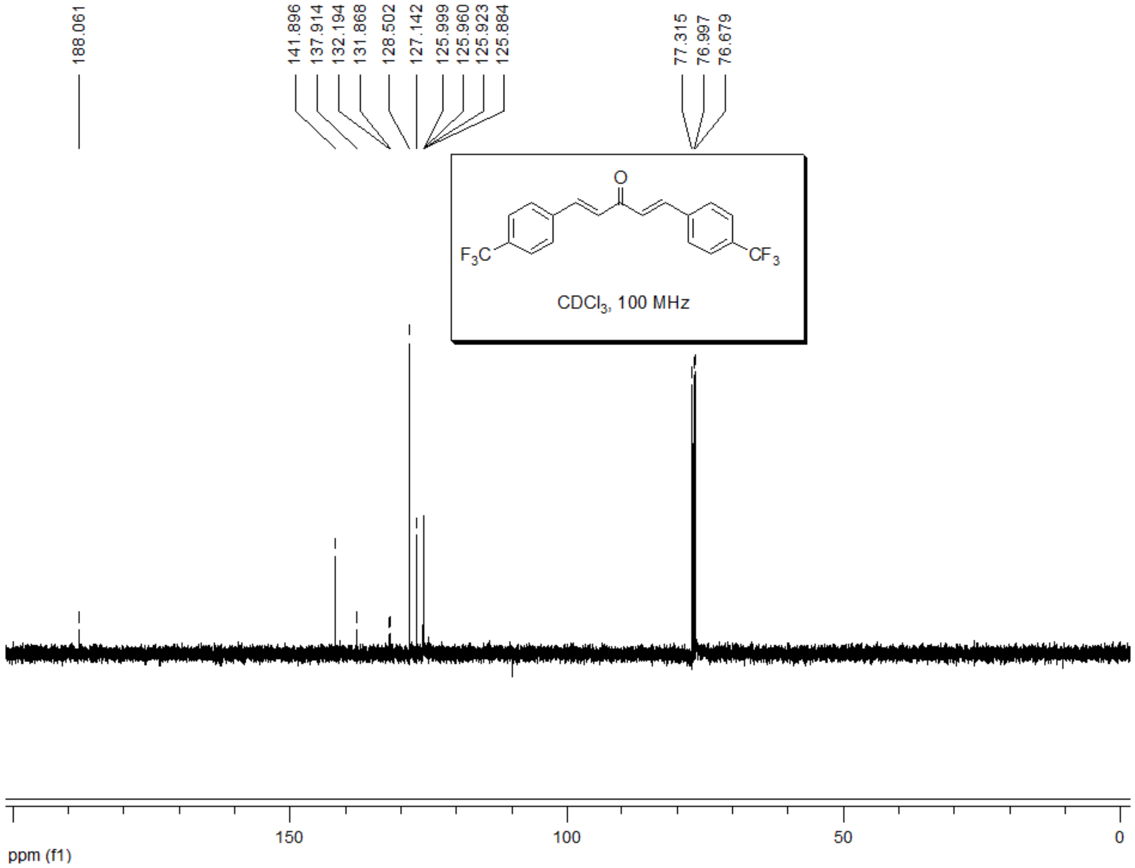
188.061
141.896
137.914
132.194
131.868
128.502
127.142
125.999
125.960
125.923
125.884
77.315
76.997
76.679
O
F_3C
CF_3
$CDCl_3$, 100 MHz
150
100
50
0
ppm (f1)

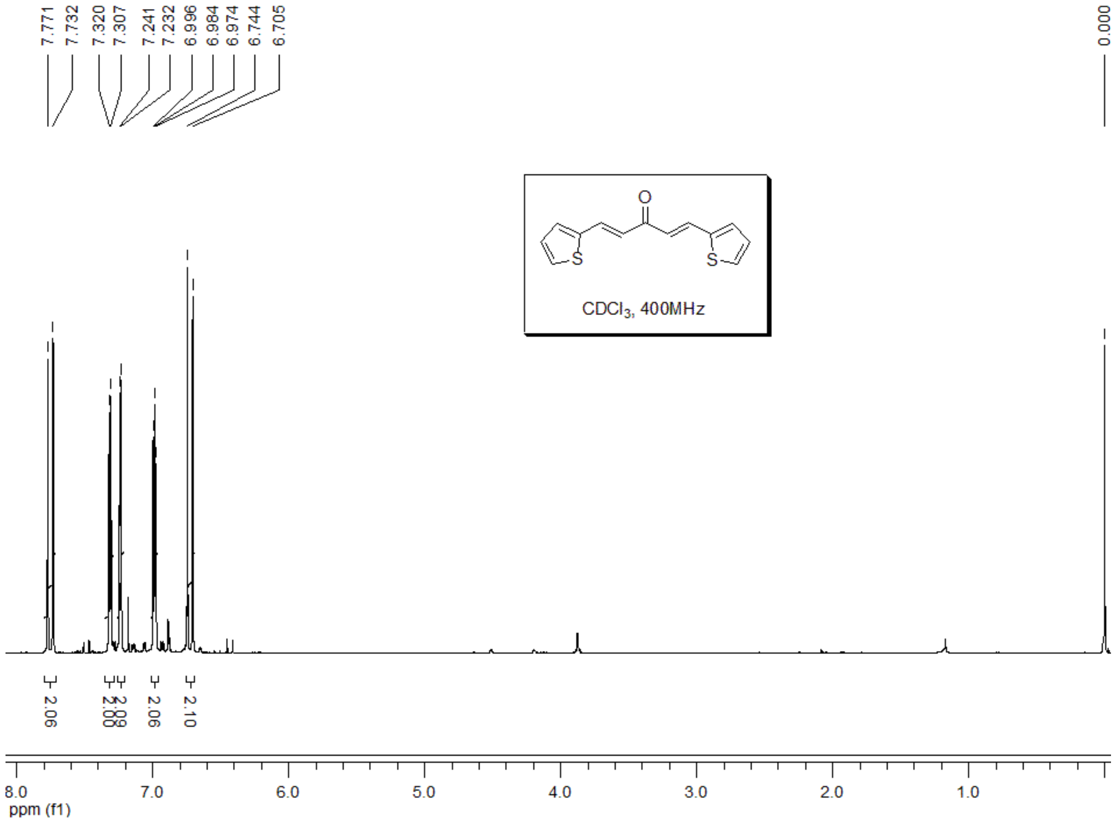
7.771
7.732
7.320
7.307
7.241
7.232
6.996
6.984
6.974
6.744
6.705
0.000
O
S
S
$CDCl_3$, 400MHz
2.06
2.06
2.10
8.0
7.0
6.0
5.0
4.0
3.0
2.0
1.0
ppm (f1)

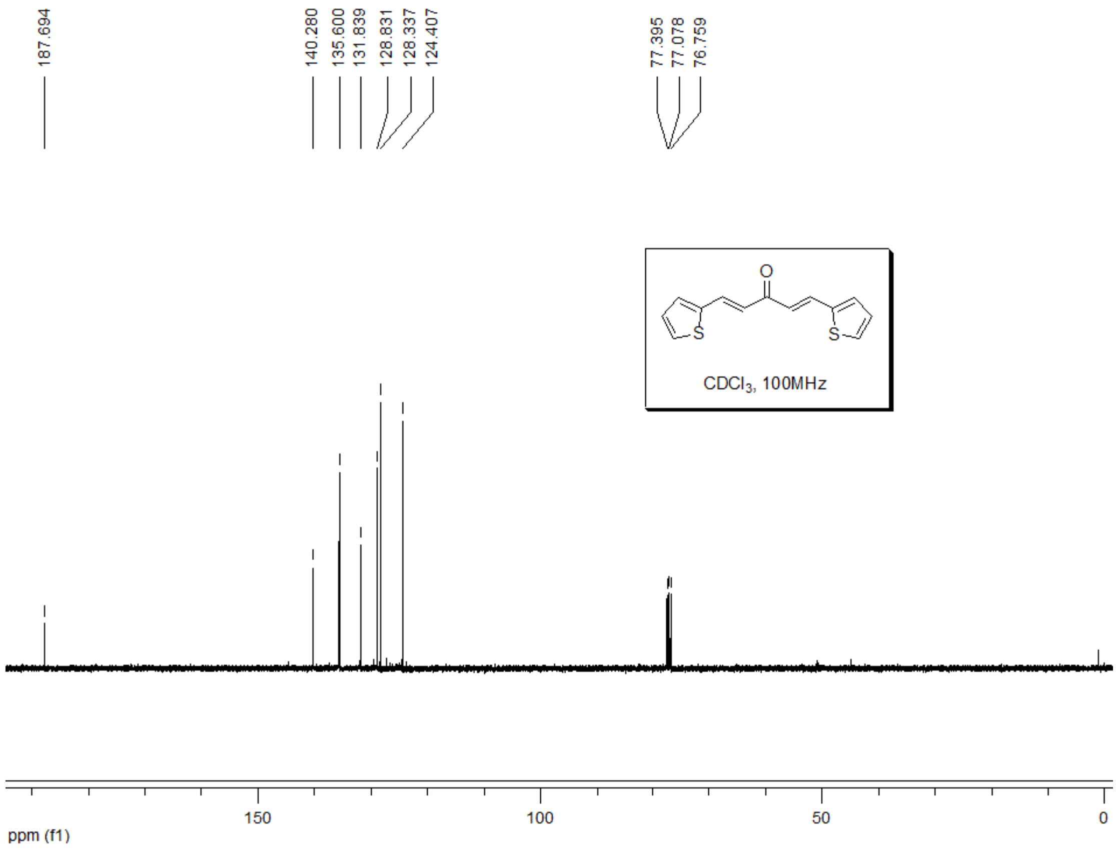
187.694
140.280
135.600
131.839
128.831
128.337
124.407
77.395
77.078
76.759
O
S
S
CDCl3, 100MHz
150
100
50
0
ppm (f1)

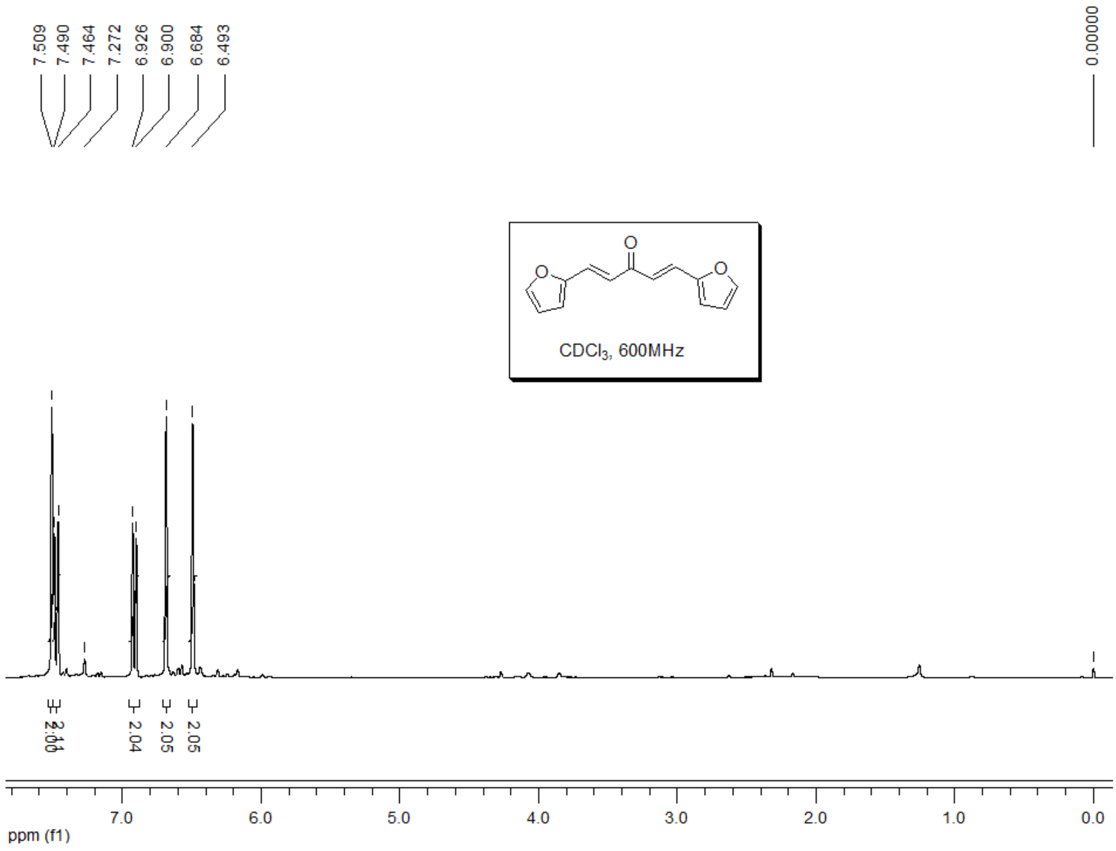
7.509
7.490
7.464
7.272
6.926
6.900
6.684
6.493
0.00000
O
O
O
CDCl3, 600MHz
2.11
2.00
2.04
2.05
2.05
7.0
6.0
5.0
4.0
3.0
2.0
1.0
0.0
ppm (f1)

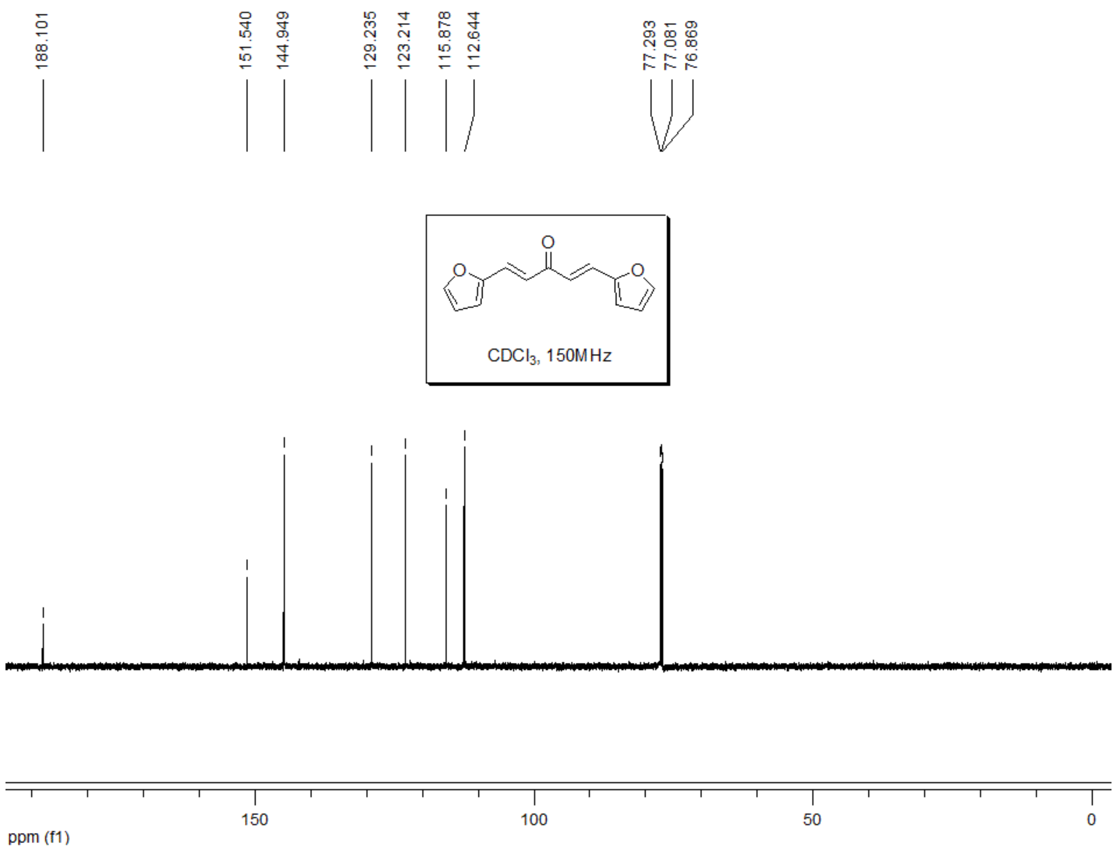
188.101
151.540
144.949
129.235
123.214
115.878
112.644
77.293
77.081
76.869
O
O
O
CDCl3, 150MHz
150
100
50
0
ppm (f1)

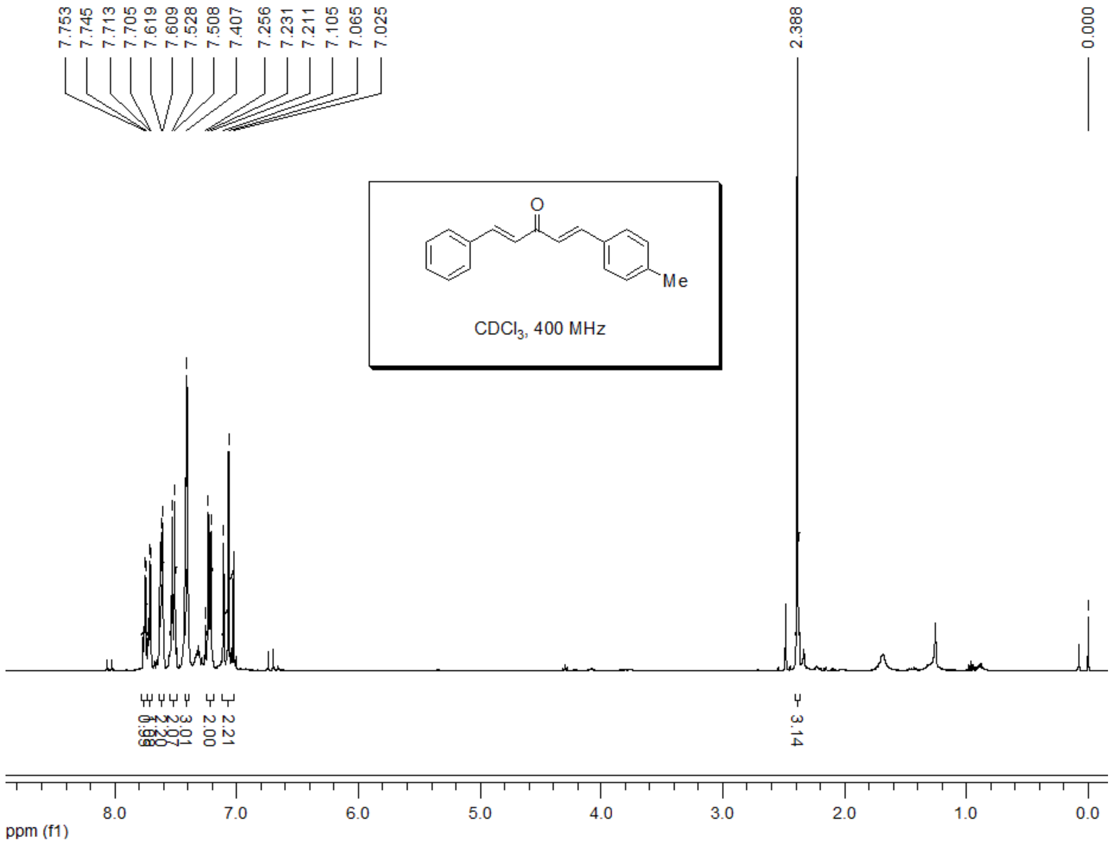
7.753
7.745
7.713
7.705
7.619
7.609
7.528
7.508
7.407
7.256
7.231
7.211
7.105
7.065
7.025
2.388
0.000
O
Me
CDCl3, 400 MHz
3.01
2.00
2.21
3.14
8.0
7.0
6.0
5.0
4.0
3.0
2.0
1.0
0.0
ppm (f1)

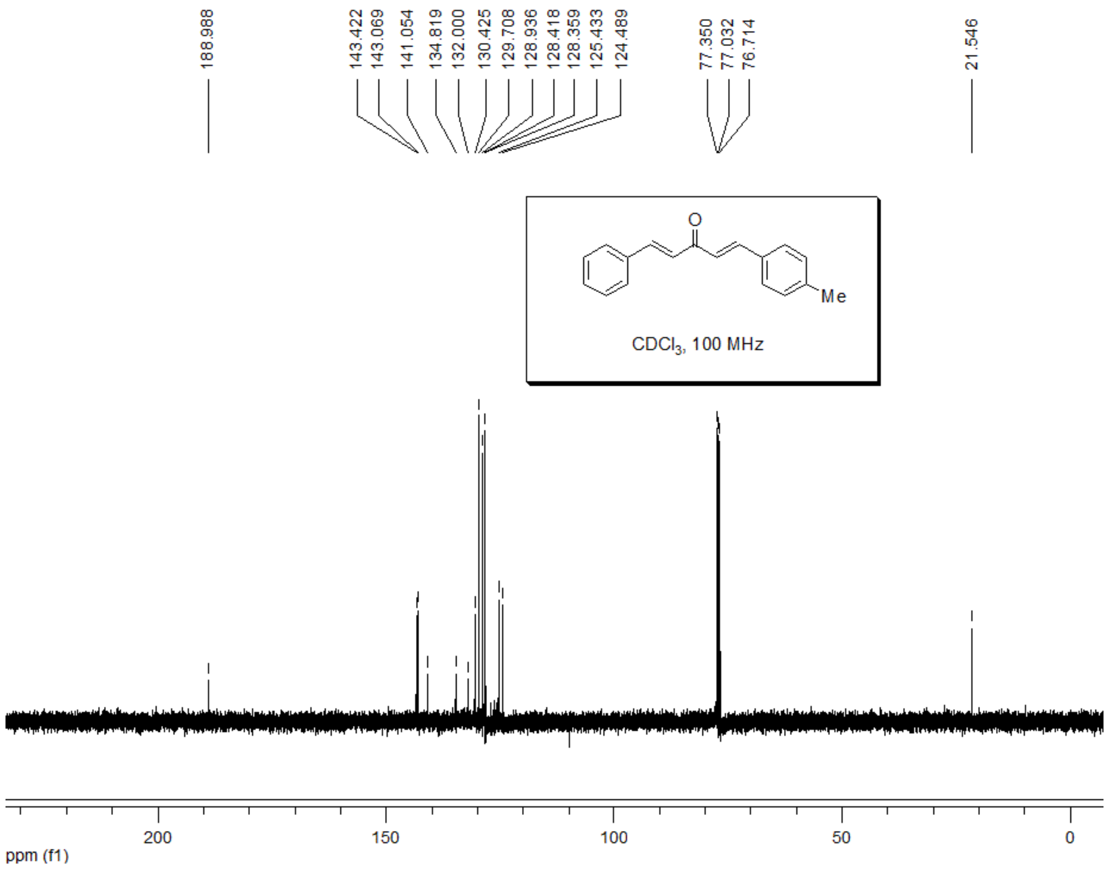
188.988
143.422
143.069
141.054
134.819
132.000
130.425
129.708
128.936
128.418
128.359
125.433
124.489
77.350
77.032
76.714
21.546
O
Me
CDCl3, 100 MHz
200
150
100
50
0
ppm (f1)

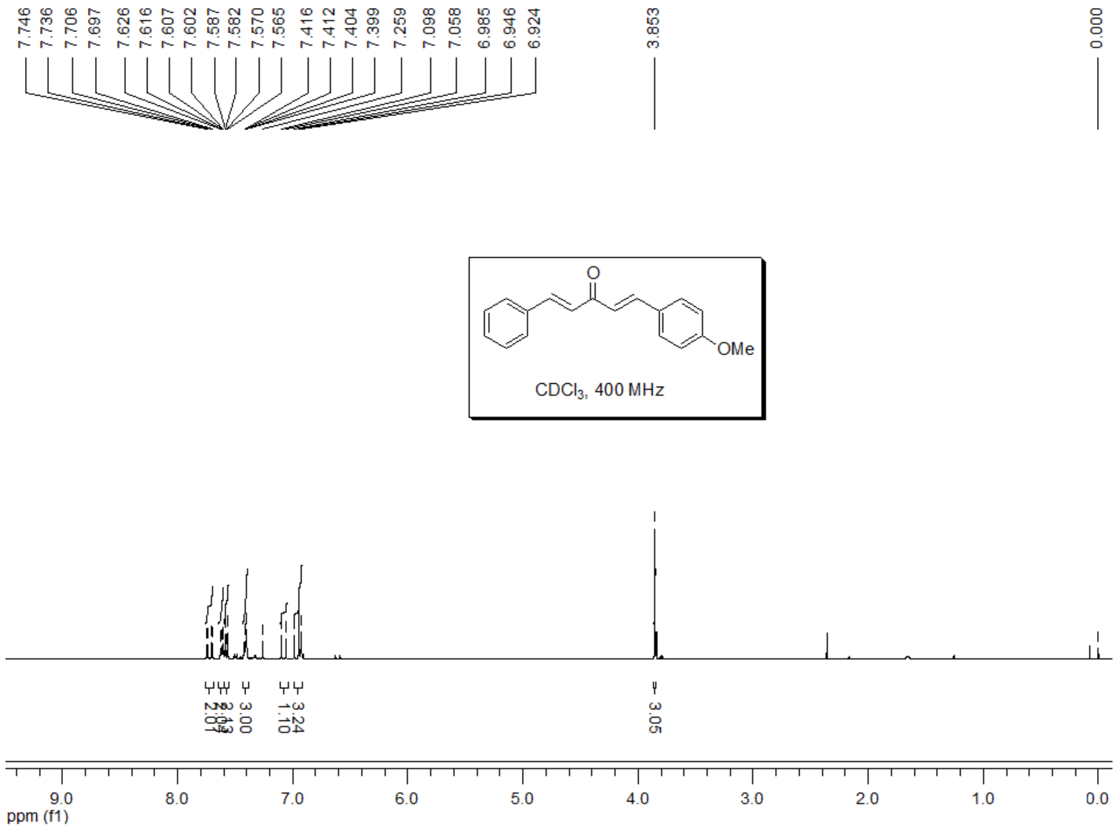
7.746
7.736
7.706
7.697
7.626
7.616
7.607
7.602
7.587
7.582
7.570
7.565
7.416
7.412
7.404
7.399
7.259
7.098
7.058
6.985
6.946
6.924
3.853
0.000
O
OMe
CDCl3, 400 MHz
3.00
1.10
3.24
3.05
9.0
8.0
7.0
6.0
5.0
4.0
3.0
2.0
1.0
0.0
ppm (f1)

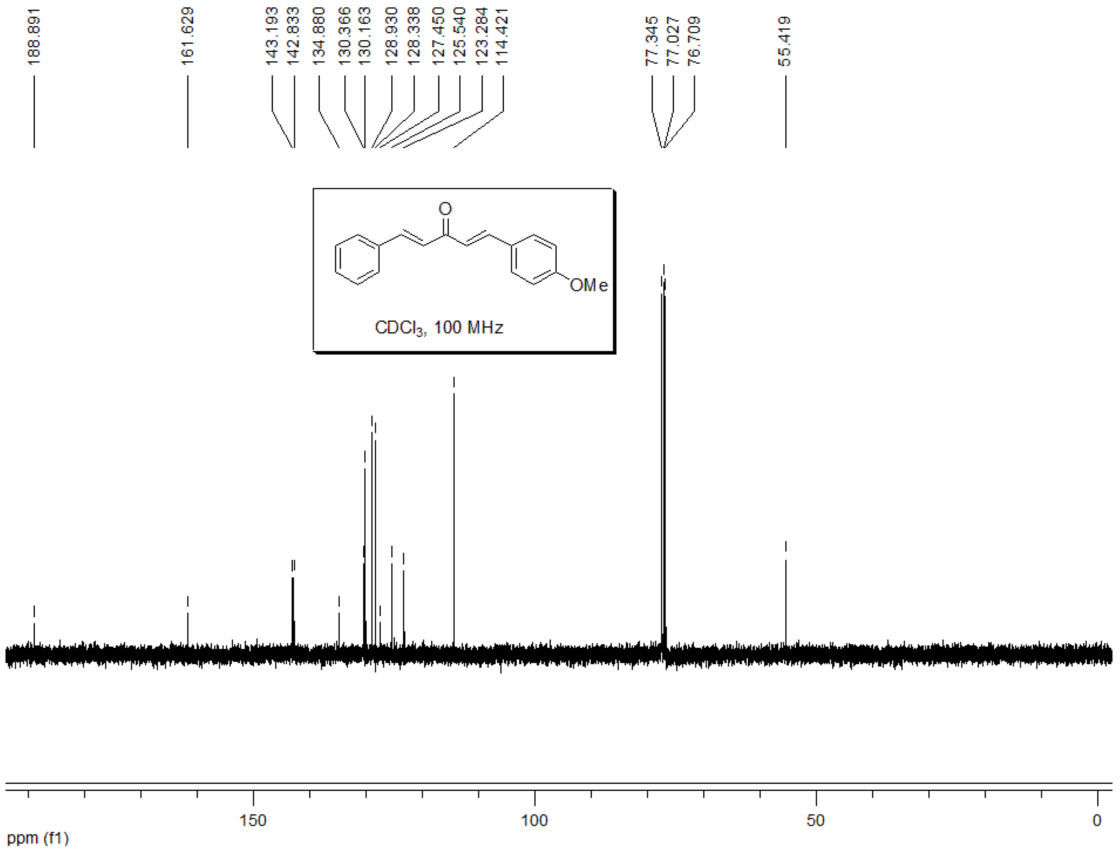
188.891
161.629
143.193
142.833
134.880
130.366
130.163
128.930
128.338
127.450
125.540
123.284
114.421
77.345
77.027
76.709
55.419
O
OMe
CDCl3, 100 MHz
150
100
50
0
ppm (f1)

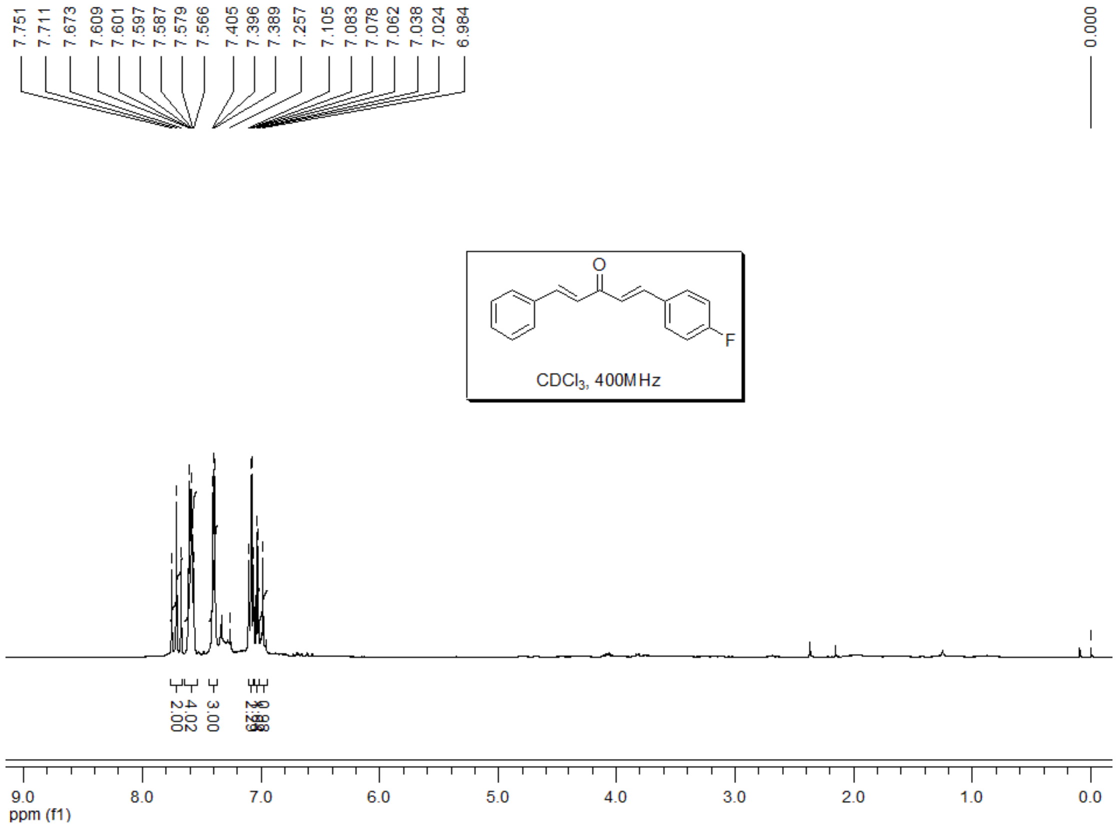
7.751
7.711
7.673
7.609
7.601
7.597
7.587
7.579
7.566
7.405
7.396
7.389
7.257
7.105
7.083
7.078
7.062
7.038
7.024
6.984
0.000
O
F
CDCl3, 400MHz
2.00
4.02
3.00
9.0
8.0
7.0
6.0
5.0
4.0
3.0
2.0
1.0
0.0
ppm (f1)

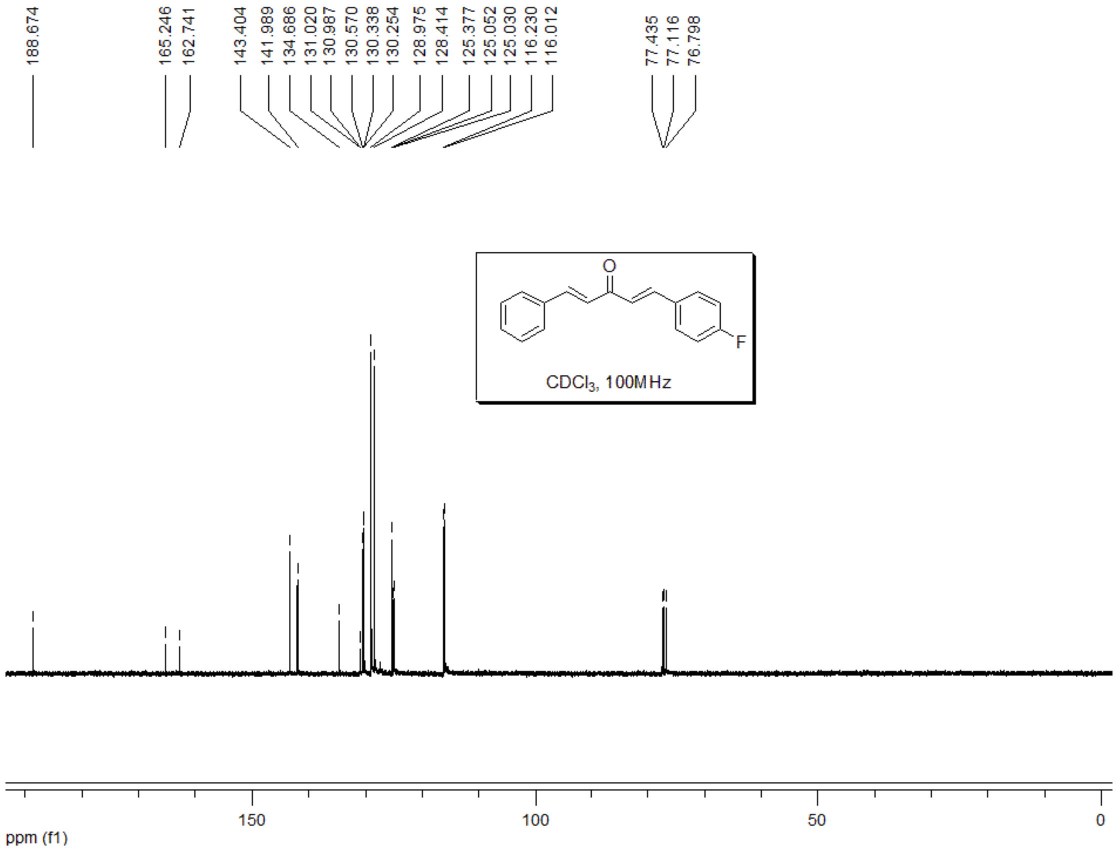
188.674
165.246
162.741
143.404
141.989
134.686
131.020
130.987
130.570
130.338
130.254
128.975
128.414
125.377
125.052
125.030
116.230
116.012
77.435
77.116
76.798
O
F
CDCl3, 100MHz
150
100
50
0
ppm (f1)

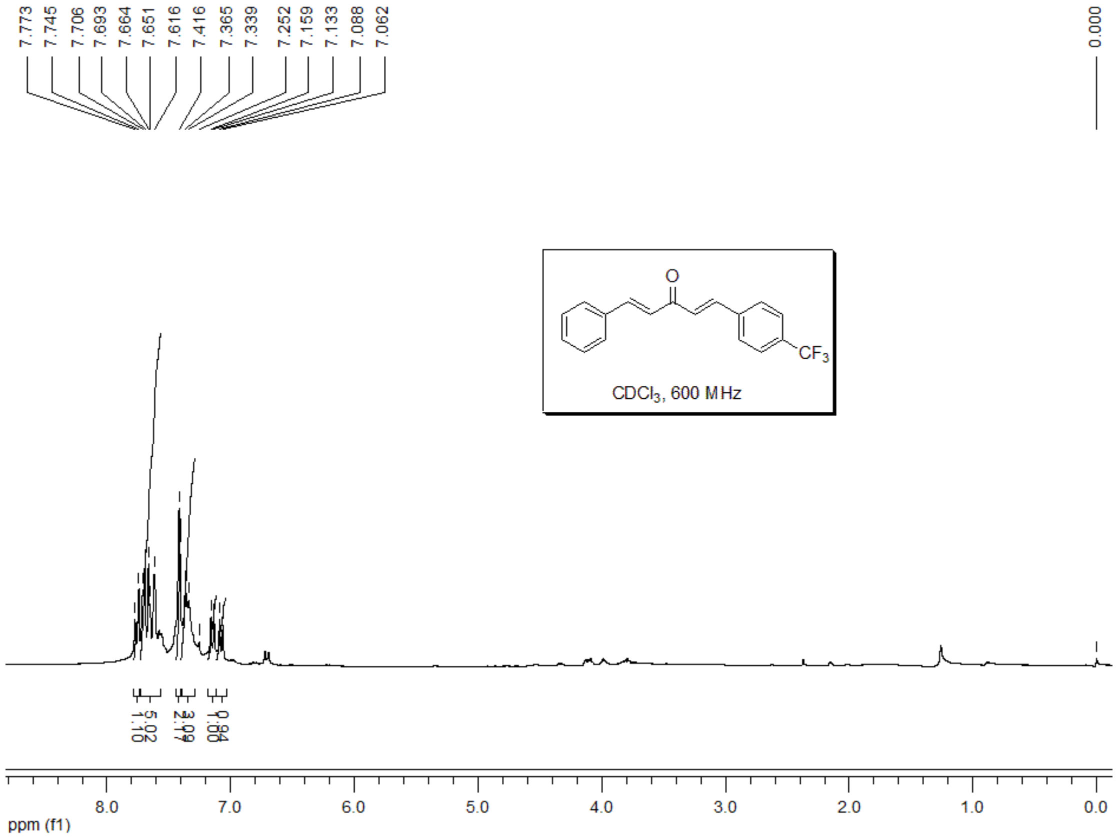
7.773
7.745
7.706
7.693
7.664
7.651
7.616
7.416
7.365
7.339
7.252
7.159
7.133
7.088
7.062
0.000
O
CF3
CDCl3, 600 MHz
1.10
5.02
2.17
3.09
1.00
0.94
8.0
7.0
6.0
5.0
4.0
3.0
2.0
1.0
0.0
ppm (f1)

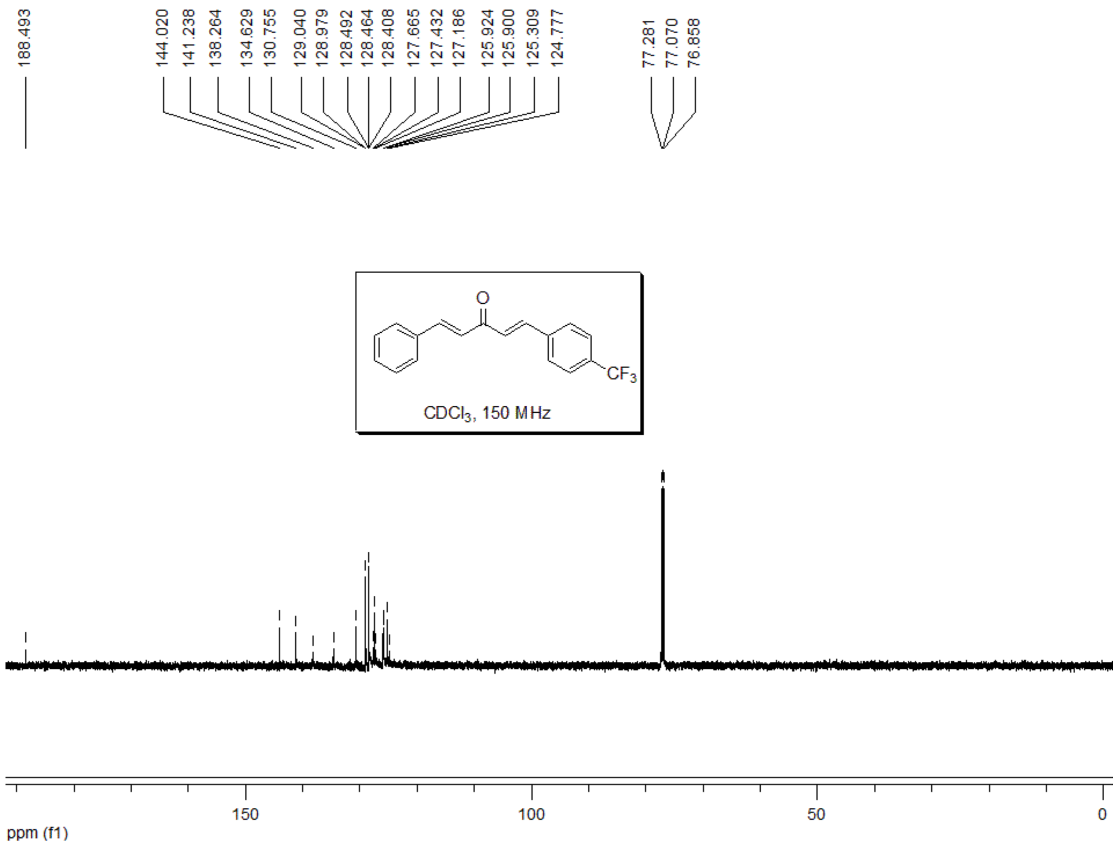
188.493
144.020
141.238
138.264
134.629
130.755
129.040
128.979
128.492
128.464
128.408
127.665
127.432
127.186
125.924
125.900
125.309
124.777
77.281
77.070
76.858
O
CF3
CDCl3, 150 MHz
150
100
50
0
ppm (f1)

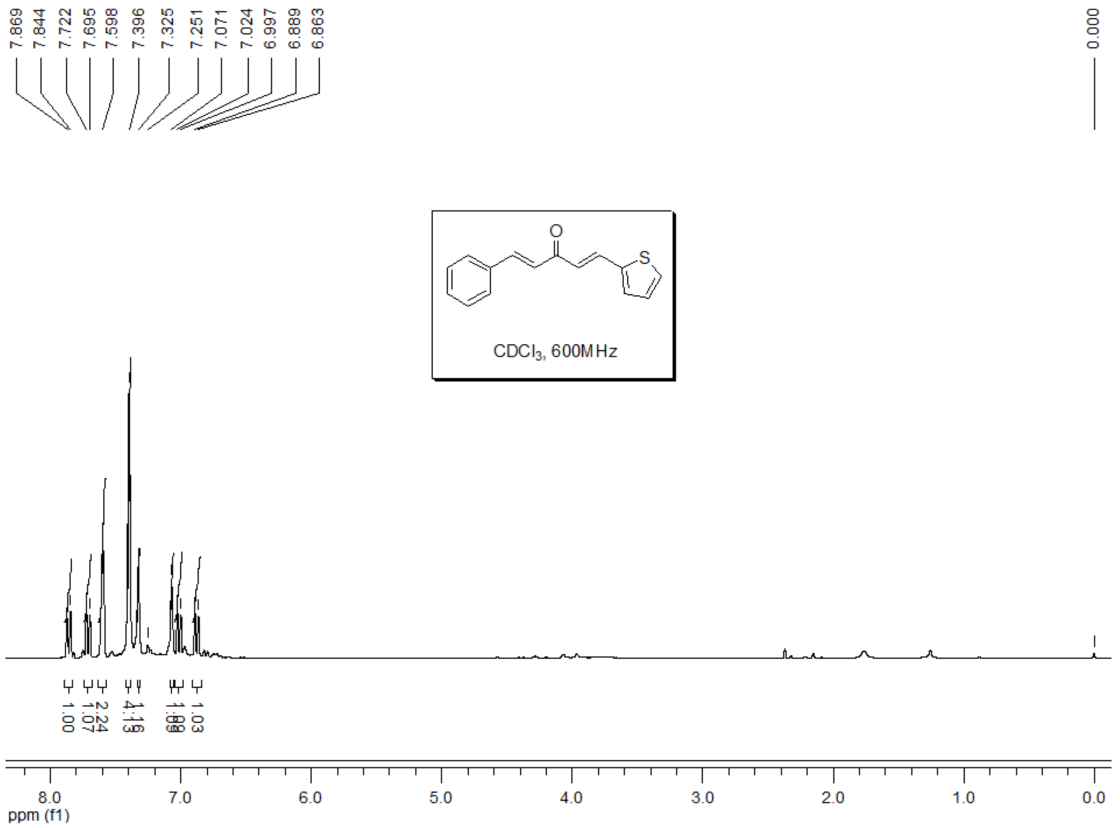
7.869
7.844
7.722
7.695
7.598
7.396
7.325
7.251
7.071
7.024
6.997
6.889
6.863
0.000
O
S
CDCl3, 600MHz
1.00
1.07
2.24
4.13
1.16
1.09
1.08
1.03
8.0
7.0
6.0
5.0
4.0
3.0
2.0
1.0
0.0
ppm (f1)

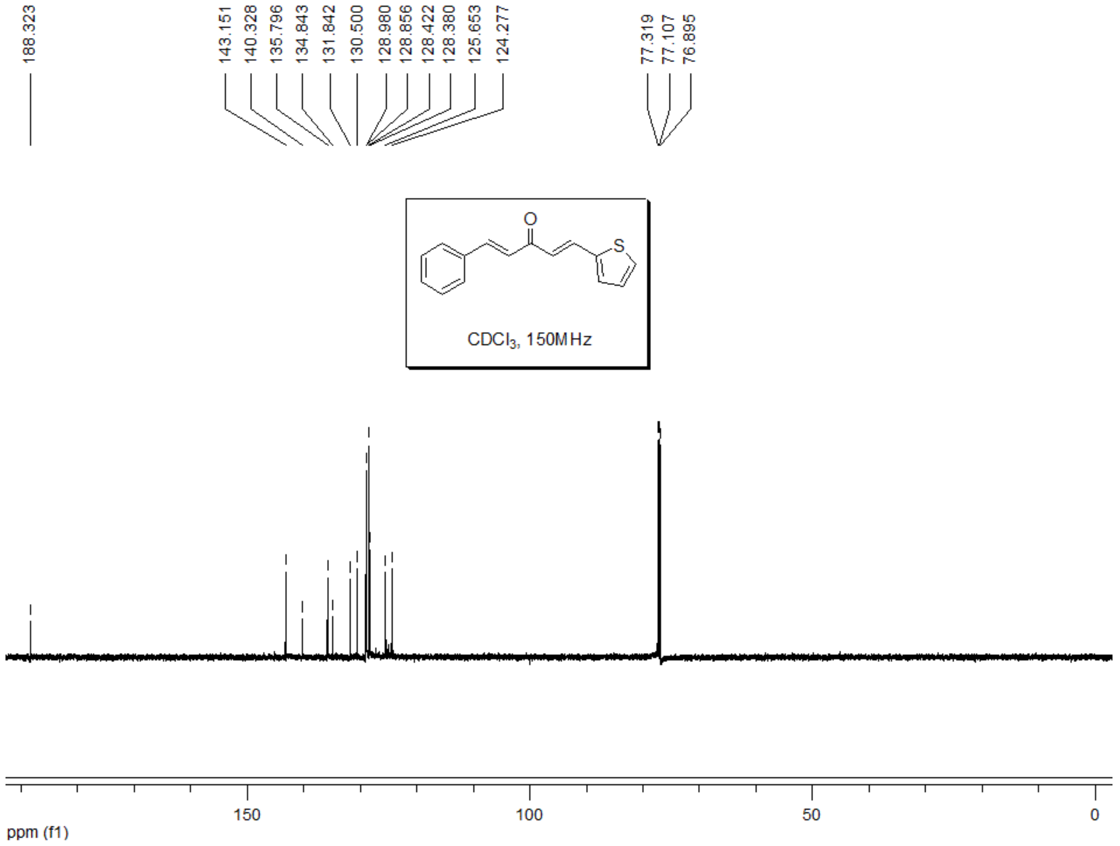
188.323
143.151
140.328
135.796
134.843
131.842
130.500
128.980
128.856
128.422
128.380
125.653
124.277
77.319
77.107
76.895
O
S
CDCl3, 150MHz
150
100
50
0
ppm (f1)

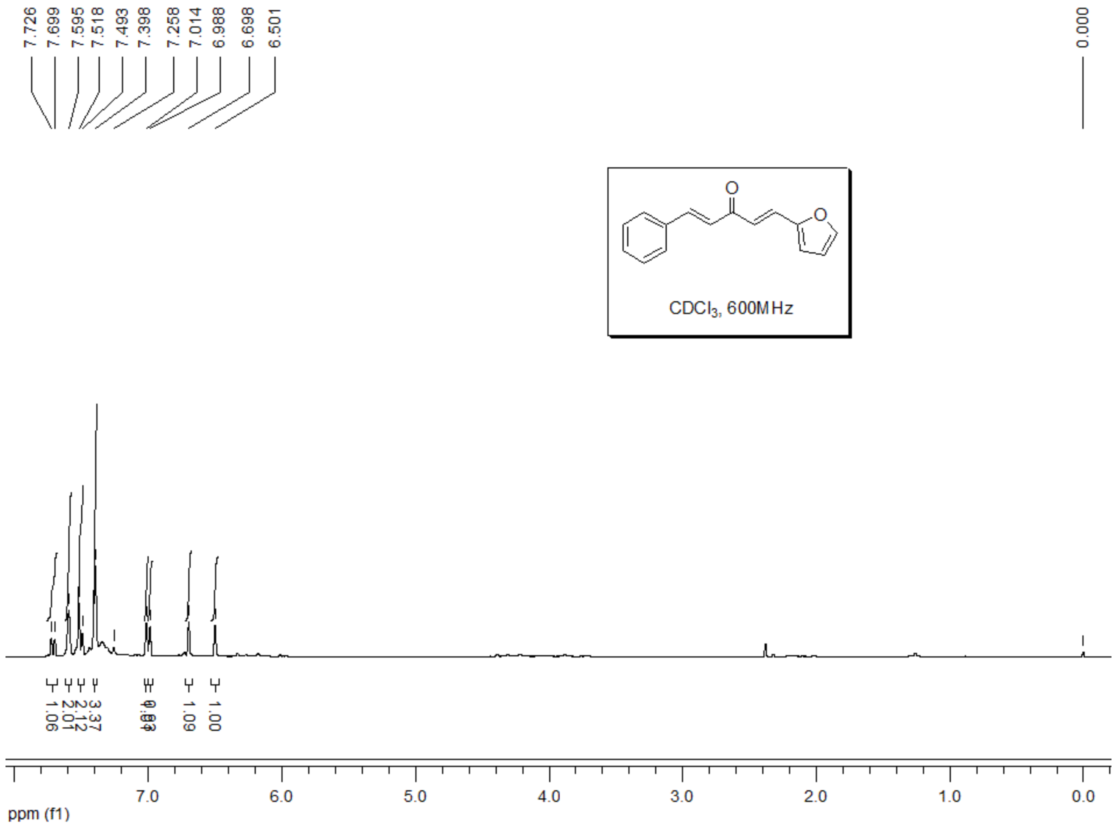
7.726
7.699
7.595
7.518
7.493
7.398
7.258
7.014
6.988
6.698
6.501
0.000
CDCl3, 600MHz
1.06
2.01
2.12
3.37
1.09
1.00
7.0
6.0
5.0
4.0
3.0
2.0
1.0
0.0
ppm (f1)

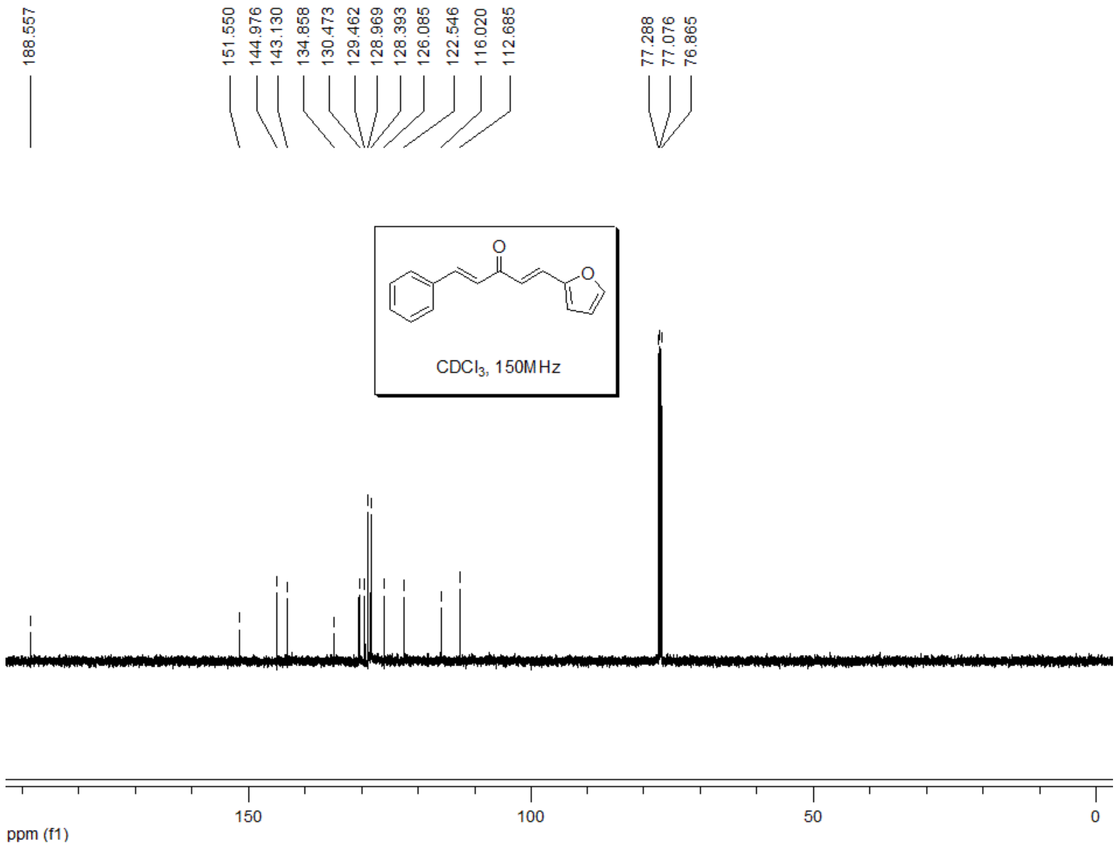
188.557
151.550
144.976
143.130
134.858
130.473
129.462
128.969
128.393
126.085
122.546
116.020
112.685
77.288
77.076
76.865
CDCl3, 150MHz
150
100
50
0
ppm (f1)

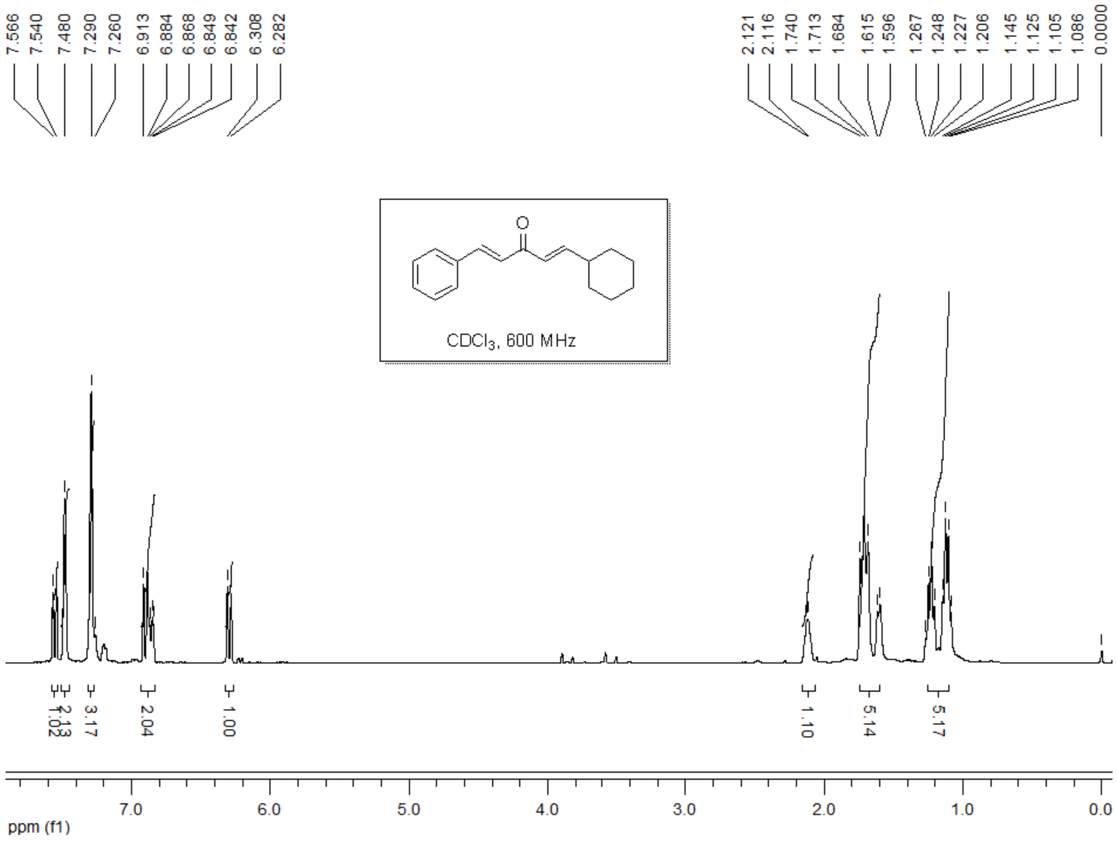
7.566
7.540
7.480
7.290
7.260
6.913
6.884
6.868
6.849
6.842
6.308
6.282
2.121
2.116
1.740
1.713
1.684
1.615
1.596
1.267
1.248
1.227
1.206
1.145
1.125
1.105
1.086
0.0000
O
$CDCl_3$, 600 MHz
1.02
2.13
3.17
2.04
1.00
1.10
5.14
5.17
7.0
6.0
5.0
4.0
3.0
2.0
1.0
0.0
ppm (f1)

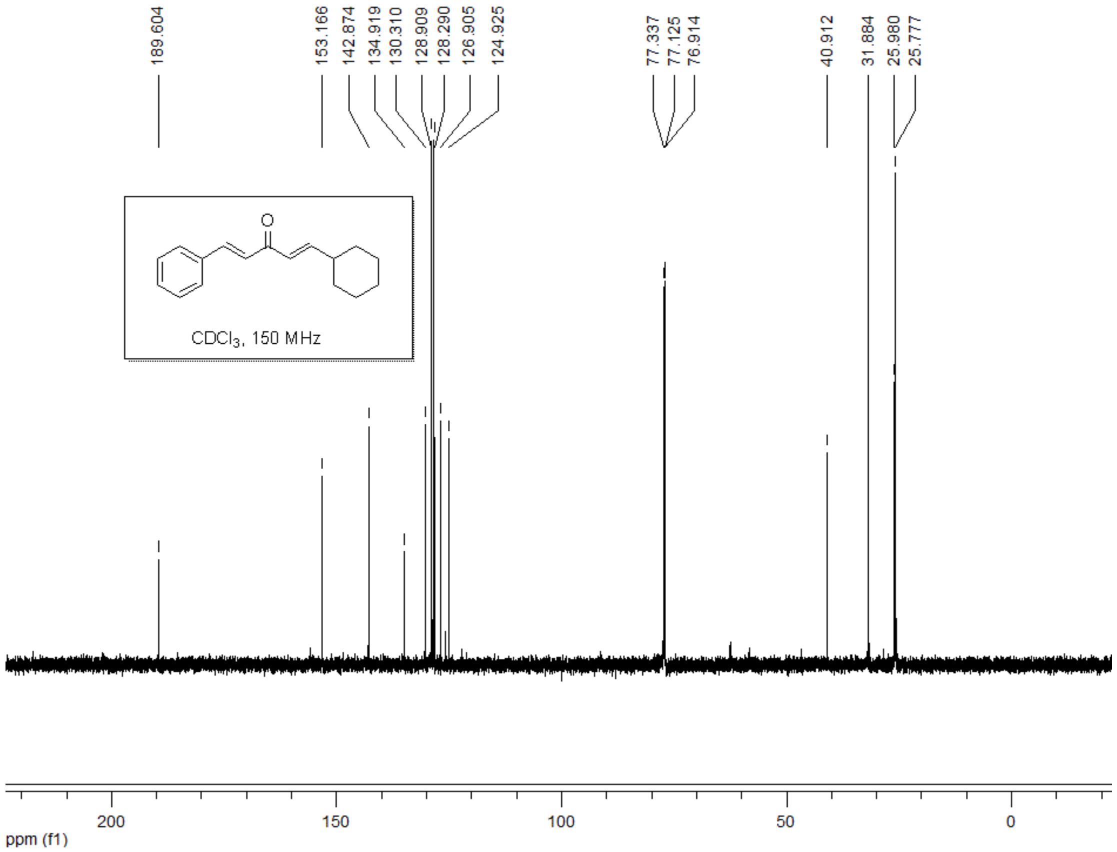
189.604
153.166
142.874
134.919
130.310
128.909
128.290
126.905
124.925
77.337
77.125
76.914
40.912
31.884
25.980
25.777
O
$CDCl_3$, 150 MHz
200
150
100
50
0
ppm (f1)
